# Antibacterial activity and mechanism of chelerythrine against *Streptococcus agalactiae*

**DOI:** 10.3389/fvets.2024.1408376

**Published:** 2024-06-14

**Authors:** Jige Xin, Qiqi Pu, Ruiying Wang, Yeqing Gu, Lin He, Xuan Du, Guowen Tang, Diangang Han

**Affiliations:** ^1^College of Veterinary Medicine, Yunnan Agricultural University, Kunming, China; ^2^College of Plant Protection, Yunnan Agricultural University, Kunming, China; ^3^Technology Center of Kunming Customs, Kunming, China

**Keywords:** antibacterial activity, antibacterial mechanism, chelerythrine, chelerythrine chloride, *Streptococcus agalactiae*

## Abstract

*Streptococcus agalactiae* (*S.agalactiae*), also known as group B *Streptococcus* (GBS), is a highly infectious pathogen. Prolonged antibiotic usage leads to significant issues of antibiotic residue and resistance. Chelerythrine (CHE) is a naturally occurring benzophenidine alkaloid and chelerythrine chloride (CHEC) is its hydrochloride form with diverse biological and pharmacological activities. However, the antibacterial mechanism of CHEC against GBS remains unclear. Thus, this study aims to investigate the *in vitro* antibacterial activity of CHEC on GBS and elucidate its underlying mechanism. The antibacterial effect of CHEC on GBS was assessed using inhibitory zone, minimum inhibitory concentration (MIC), and minimum bactericidal concentration (MBC) assays, as well as by constructing a time-kill curve. The antibacterial mechanism of CHEC was investigated through techniques such as scanning electron microscopy (SEM) and transmission electron microscopy (TEM), measurement of alkaline phosphatase (AKP) activity, determination of Na^+^ K^+^, Ca^2+^ Mg^2+^—adenosine triphosphate (ATP) activity, observation of membrane permeability, and analysis of intracellular reactive oxygen species (ROS) and mRNA expression levels of key virulence genes. The results demonstrated that the inhibition zone diameters of CHEC against GBS were 14.32 mm, 12.67 mm, and 10.76 mm at concentrations of 2 mg/mL, 1 mg/mL, and 0.5 mg/mL, respectively. The MIC and MBC values were determined as 256 μg/mL and 512 μg/mL correspondingly. In the time-kill curve, 8 × MIC, 4 × MIC and 2 × MIC CHEC could completely kill GBS within 24 h. SEM and TEM analyses revealed significant morphological alterations in GBS cells treated with CHEC including shrinkage, collapse, and leakage of cellular fluids. Furthermore, the antibacterial mechanism underlying CHEC’s efficacy against GBS was attributed to its disruption of cell wall integrity as well as membrane permeability resulting in extracellular release of intracellular ATP, AKP, Na^+^ K^+^, Ca^2+^ Mg^2+^. Additionally CHEC could increase the ROS production leading to oxidative damage and downregulating mRNA expression levels of key virulence genes in GBS cells. In conclusion, CHEC holds potential as an antimicrobial agent against GBS and further investigations are necessary to elucidate additional molecular mechanisms.

## 1 Introduction

*Streptococcus agalactiae (S.agalactiae)*, also known as Group B Streptococcus (GBS), is a gram-positive facultative anaerobe, commonly observed in chains or diplococci formations when viewed under the microscope (1, 2). GBS showed a broad spectrum of host infectivity. It is a commonly found in humans, livestock and aquatic products and can be widely spread among various animals, such as cattle and fish. The pathogen has the potential to induce meningoencephalitis in fish, mastitis in dairy cows, endometritis in women, as well as sepsis, meningitis, and pneumonia in newborns (3). The findings of various studies have demonstrated a remarkable similarity in the dominant serotypes of GBS isolated from humans, cattle, and fish, indicating a high degree of genetic correlation and homology. These results suggest the potential transmission of GBS among humans, animals, and fish (4). Dairy cow mastitis is a leading cause of economic losses in the dairy industry due to its negative impact on milk yield, fresh milk quality, cow longevity, treatment and feeding costs, as well as increased culling rates. These factors can impede the development of dairy farming and elevate breeding expenses (5, 6). The etiology of mastitis in dairy cows is complex, and pathogenic bacteria are the most important pathogenic factors. GBS is a common clinical and subclinical pathogen in cattle. In veterinary clinics, antibiotics are commonly utilized for the treatment of mastitis, particularly in cases of acute and subacute mastitis. Favorable outcomes can be achieved through intravenous administration of antibiotics, with drugs also being infused post-milking in the affected area. The use of penicillins, quinolones, and other drugs is prevalent in veterinary clinics for the treatment of dairy cow mastitis. However, there has been an observed increasing trend in the resistance rate of GBS to antibiotics over the years (7). Tian et al. (8) conducted a study in which they tested 735 raw milk samples of dairy cows collected from 11 provinces in China, and the streptococcal infection status, distribution and drug resistance of isolates and the presence of drug resistance genes and virulence genes were evaluated. *Streptococcal* isolates have been found to have high antimicrobial resistance and carry various virulence genes that may be harmful to both cows and humans. Isolates from 305 milk samples of goats with mastitis collected by Shi et al. (9) in China were subjected to quinolone drug sensitivity testing and genetic testing, and the results showed significant resistance to quinolone drugs.

*Macleaya cordata* (MC) belongs to the *Papaveraceae* family and is extensively utilized in Chinese herbal medicine worldwide (10). Numerous studies have demonstrated that MC and its extracts are employed in animal feed as a substitute for antibiotics, exhibiting remarkable antibacterial, antioxidant, immune-modulating, anti-inflammatory properties while reducing livestock and poultry diseases and enhancing their performance and product quality (11). MC exhibits numerous advantages, including a broad antibacterial spectrum, absence of drug resistance and residue, excellent safety profile, and wide distribution. Moreover, MC is abundant in alkaloids and other bioactive compounds that hold significant potential for further development. Its main functional active ingredients are chelerythrine (CHE), sanguinarine (SA), protopine (PRO), allocryptopine (ALL) and other alkaloids (12). As an alkaloid widely present in natural plants, significant advancements has been achieved in the research of CHE, which exhibits a diverse range of pharmacological activities including anti-tumor, antibacterial, and anti-inflammatory effects. These bioactivities are widely applied in animal husbandry, agriculture, aquaculture, and related domains. CHE has demonstrated a broad spectrum of antibacterial activities, effectively inhibiting various pathogenic bacteria including gram-positive bacteria and *Escherichia coli*. Furthermore, CHE and its derivatives exhibit varying degrees of inhibition against parasite (13). Qian et al. (14) observed that CHE exhibited significant inhibitory effects on *Staphylococcus aureus* and *Staphylococcus lugdunensis*, with notable biofilm clearance properties. Additionally, CHE possesses diverse biological functions such as anti-inflammatory, antioxidative, and antitumour activities in the field of medicine, showcasing promising prospects for drug development and application (15). Studies have shown that CHE can prevent excessive inflammatory immune response by regulating the key signaling pathways involved in SARS-CoV-2 infection, and can fight SARS-CoV-2 through a variety of mechanisms, making it very potential to become one of the candidate drugs for the treatment of novel coronaviruses with high development value (16). Since SA and CHE themselves are positively charged and unstable in aqueous solution, they are typically converted into bisulfate or hydrochloride forms for increased stability during practical use. CHE can be obtained by volatile natural deep eutectic solvents (17). Chelerythrine chloride (CHEC), also known as a quaternary ammonium base, Qian et al. (18) found that CHE inhibited the growth of carbapenem-resistant *Serratia marcescens* through disrupting cell membrane integrity and cell morphology. This provides a research basis for further study on antimicrobial activity of CHE.

Currently, several studies have been reported on the inhibitory and bactericidal effects of Chinese herbs against GBS. For instance, herbal mixtures (*Astragalus*, *Angelica sinensis*, and *Hubei hawthorn*) improved survival of tilapia free of *Streptococcus lactis* infection (19). Berberine effectively suppresses the *in vitro* proliferation of GBS by compromising cellular integrity and inhibit synthesis of protein and DNA to achieve antibacterial efficacy (20). SA disrupts the cell membrane integrity and permeability of GBS, resulting in cell lysis and slight intracellular substance leakage for antibacterial effects (21). CHE can inhibit a variety of bacteria or fungi, but its antibacterial effect and mechanism against GBS remains unclear. Although Chinese herbal medicine shows potential as an alternative to antibiotics, there are still numerous challenges that need to be addressed, including understanding the effects and mechanisms of bioactive substances in Chinese herbal medicine additives. Therefore, CHEC, extracted from Chinese herbal *Macleaya cordata* was selected as the research subject in this study. The primary focus of this investigation is to explore the antibacterial efficacy of CHEC against GBS by assessing its impact on bacterial morphology, active enzymes, cell wall integrity, cell membrane function, and ROS levels *in vitro*, which will provide a fundamental theoretical basis for utilizing CHE as an active ingredient and developing clinical applications against GBS infections.

## 2 Materials and methods

### 2.1 Source and preparation of strains and reagents

The *Streptococcus agalactiae* strain (GBS, ATCC13813) utilized in this trial was procured from Beina Biotechnology Co., LTD. Initially, the GBS strain was activated and then added to a sterile test tube containing 5 mL of Brain-Heart Infusion Broth (BHI) medium using a sterile inoculation ring. Subsequently, the test tube was incubated overnight at 37°C in a constant temperature shaker, and on the following day, the tube containing the bacterial solution was retrieved. Lastly, the bacterial solution was diluted to 0.5 Malcolm turbidity by Malcolm turbidimetric tube method, and the bacterial solution was diluted 1:100 to a concentration of about 1.0 × 10^6^ CFU/mL as the experimental bacterial suspension.

The CHEC used in this test was purchased from Chengdu Plant Standard Pure Biotechnology Co., LTD. (C_21_H_18_ClNO_4_, PCS0121, HPLC ≥98%, Chengdu, China). The positive control Chlortetracycline Hydrochloride (CTC) was obtained from McLean Biochemical Technology Co., LTD. (C804422, Shanghai, China). For the experiment, 20% DMSO was employed as a co-solvent. A reserve drug solution of CHEC at a concentration of 10 mg/mL and CTC at a concentration of 10 mg/mL were prepared and stored at −20°C and 4°C, respectively.

### 2.2 Determination of the bacteriostatic zone

The test bacterial solution (100 μL) was added to a sterile petri dish, followed by the addition of sterilized agar medium (15–20 mL) into each plate. The mixture was quickly and evenly mixed. After cooling and solidification of the agar, holes with a diameter of 6 mm were punched on the agar plate using a sterile hole punch, ensuring a distance of more than 2.5 cm between the holes. Agar from the holes was removed using a sterile needle, and equal volumes (15 μL) of CHEC samples with different mass concentrations (0.5, 1, and 2 mg/mL) were added into each hole. A positive control (2 mg/mL CTC) and a negative control (3% DMSO) were included, with three replicates performed for each condition. Subsequently, following drug absorption, the petri dishes were inverted and incubated at 37°C for 24 h prior to observation. The total diameter of the inhibition zone was measured using a Vernier caliper, with the outer edge of the inhibition zone serving as the boundary. The diameter of the inhibition zone was calculated as the total diameter minus the diameter of the hole, and the average value was obtained by conducting three experiments.

### 2.3 Determination of MIC and MBC

According to the standards recommended by the American Clinical Laboratory Standardization Association (CLSI) and following the method described in Majolo et al. (22), the broth dilution in 96-well plates method was conducted to determine the MIC of CHEC against GBS. Sterile disposable unopened 96-well plates were utilized, where well 1 served as a negative control and received 100 μL of 3% DMSO followed by 100 μL of BHI. Using the microdilution method, wells 2 to 12 were supplemented with 100 μL of BHI. Subsequently, well No.2 received 100 μL of diluted CHEC (initial concentration:4096 μg/mL), which was thoroughly mixed using a pipettor. Then, 100 μL solution from well No. 2 was transferred to well No. 3. This process continued until reaching well No. 11. Finally, each test Wells from No. 2 to No. 12 received an additional 100 μL of diluted logarithmic phase bacterial solution. Meanwhile, well No. 12 without CHEC served as a positive control for comparison purposes. Since BHI medium, bacterial solution and drug solutions were mixed in equal proportions, resulting in a double dilution of the drug. The final tested drug concentrations consisted of eleven gradients: specifically, 1,024, 512, 256, 128, 64, 32, 16, 8, 4, 2, and 0 μg/mL. Three independent replicates were performed for each treatment group. The incubation period lasted for approximately 18–24 h at 37°C, followed by observation and comparison of results. Clear wells observed visually were recorded as MIC and the experiment was repeated for 3 times.

A total of 100 μL of the culture suspension from the above wells without bacterial growth was placed in a Petri dish, quickly mixed with 15–20 mL agar medium, and incubated at 37°C for 18–24 h to observe whether colonies could be formed. The concentration at which there was no colony growth at all was defined as MBC.

### 2.4 Plotting of time-kill curves

Five hundred microliters of bacterial suspension and 500 μL of CHEC were vigorously shaken and homogenized to achieve final concentrations of 0 × MIC, 1 × MIC, 2 × MIC, 4 × MIC, and 8 × MIC, respectively. A negative control containing 1 mL of 3% DMSO was included. Bacterial cultures were incubated at 37°C in a constant temperature shaker for 0, 3, 6, 12, and 24 h. At each time point, a tenfold dilution series (5 times) was prepared by diluting the bacterial suspension with appropriate volumes. For each dilution, 10 μL of bacterial suspension was placed in a Petri dish and quickly mixed with 15 mL of agar medium. After complete absorption on agar plates, they were invertedly cultured at 37°C for approximately18–24 h. Colonies ranging from 20 to 300 were counted to calculate average CFU and the average value was obtained by conducting three experiments. The logarithmically transformed CFU values per milliliter of bacterial suspension at different time points were plotted against time using GraphPad Prism software version 9.0 to generate the time-kill curve.

### 2.5 Observation of the micromorphology changes

The effect of CHEC on the morphological changes of GBS was observed using SEM (Hitachi SEM Flex 1,000 II, Japan), and samples were prepared following the method described by Shan et al. (23). GBS bacterial solution cultured to logarithmic phase was supplemented with CHEC (0 × MIC, 2 × MIC, 4 × MIC, 8 × MIC), respectively, and the bacteria were incubated at 37°C and 150 r/min in a constant temperature shaker. One milliliter of the culture was sampled at 24 h. The cells were centrifuged at 7000 r/min for 8 min to remove the supernatant. The resulting precipitate was then mixed with 1 mL of a fixative solution containing 2.5% glutaraldehyde by vortexing for 2 min to ensure even distribution. The centrifuge tube was covered with tin foil and kept in darkness before being placed in a refrigerator at 4°C for complete fixation over a period of 12 h. After fixation, the samples were removed and centrifuged again at 7000 r/min for 8 min followed by discarding the supernatant. The precipitate obtained from previous step was resuspended in 1 mL PBS solution which underwent swirling for 2 min. This process of washing with PBS solution was repeated 3 times. These washed cells were then dehydrated gradually using an ethanol gradient eluent (15, 30, 50, 70, 80, 90%, 95%, 100%). After that, the cells were centrifugated at 5000 r/min for10 min, and the supernatant was discarded. Finally, a cover glass was selected as the carrier, and bacterial sediment was sucked onto the front surface of the cover glass using a pipettor. The sample was then left to dry naturally to obtain the powdered sample.

The conductive tape is applied onto the sample table, followed by careful attachment of the cover glass and powder onto the conductive adhesive. Subsequently, a high vacuum coating instrument (Leica, EM ACE600) is employed to coat the nonconductive sample with a layer of conductive film on its surface. The prepared sample is then mounted onto an electron microscope (Hitachi SEM Flex 1,000 II, Japan) for observation after critical drying by the system.

### 2.6 Effect of CHEC on the ultrastructure of GBS cells

Slightly modified based on the literature report by Huang et al. (24), tubes containing GBS and CHEC at various concentrations of MIC were incubated for 24 h at 37°C. The bacterial precipitate was collected by centrifugation at 8000 rpm for 5 min at 4°C and washed three times with PBS. After removing the medium, the mixture was resuspended in 2.5% glutaraldehyde and fixed for 2–4 h at 4°C. Subsequently, the mixture was rinsed with PBS for a duration of 3 min before repeating the process of centrifugation and washing three times. The cells were preembedded in a solution of 1% agar and fixed at room temperature in darkness with 1% osmic acid for 2 h. Following this step, the cells underwent another round of washing thrice with PBS. Ethanol (30, 50, 70, 80, 95, 100%) was added sequentially for 20 min each time for dehydration, and 100% acetone was added twice for 15 min each time. Infiltration was embedded overnight, after which the embedding plates were placed in an oven at 60°C for polymerization for 48 h. The resin blocks were sectioned ultrathinly at 60 to 80 nm on an ultrathin microtome (Leica, UC7, Germany) and the pieces were harvested with 150 mesh square Chinese membrane copper mesh. Finally, uranyl acetate saturated alcohol solution and lead citrate solution were used for staining. After sample preparation, the morphology of GBS cells was observed under transmission electron microscope (HITACHI, HT7800/HT7700, Japan).

### 2.7 Determination of AKP activity of GBS by CHEC

According to the method described by Liang et al. (25), the effect of CHEC treatment at different concentrations on AKP in GBS cells was determined. The GBS bacterial solution cultured to the logarithmic phase was supplemented with CHEC, resulting in final concentrations of 0 × MIC, 2 × MIC, 4 × MIC, and 8 × MIC in the bacterial suspension. The cells were then cultured at 37°C and 150 r/min using a constant temperature shaker. At culture time points of 0, 6, 12, and 24 h, 1 mL samples were taken and centrifuged at 5000 r/min for 5 min. The supernatant was obtained. The alkaline phosphatase AKP activity kit (Nanjing Jiancheng Bioengineering Institute, Jiangsu, China) was used to treat the supernatant and measure absorbance values at OD_520nm_ on a full wavelength microplate reader (Thermo Fisher Multiskan Sky, Shanghai, China). Changes in AKP activity were utilized as an indicator reflecting the impact of CHEC on the bacterial cell wall.

The formula was as follows: AKP activity (gold units/100 mL) = (measured value − blank value)/(standard value − blank value) × standard concentration (0.1 mg/mL) × 100 mL × dilution ratio. The experiment was repeated for 4 times.

### 2.8 Determination Na^+^, K^+^, Ca^2+^, Mg^2+^-ATP activities

CHEC was added to the GBS bacterial solution during logarithmic phase culture at concentrations of 0 × MIC, 2 × MIC, 4 × MIC, and 8 × MIC. At time points of 0, 6, 12, and 24 h, samples were taken and centrifuged at a rate of 5,000 r/min for 5 min. The supernatant was then analyzed using ultramicro Na^+^, K^+^, and Ca^2+^ Mg^2+^-ATPase test boxes (Nanjing Jiancheng Bioengineering Institute, Jiangsu, China), with absorbance values measured at OD_630nm_. Changes in Na^+^, K^+^, and Ca^2+^ Mg^2+^-ATP activities reflect the effect of CHEC on membrane ion transport in GBS cells.

ATPase activity (U/mgprot) = (measured value − control value)/(standard value − blank value) × C standard × 60/T × N/Cpr (C standard: 0.02 μmol/mL; T: enzymatic reaction time 10 min; N: the dilution of enzymatic reaction system 7.8; Cpr: sample protein concentration, mgprot/mL). The experiment was repeated for 3 times.

### 2.9 Observation of membrane permeability

CHEC was introduced into the GBS bacterial broth during the logarithmic growth phase, resulting in final concentrations of 0 × MIC, 2 × MIC, 4 × MIC, and 8 × MIC in the bacterial suspension. The cells were cultured at 37°C using a shaker incubator. At time points of 0, 6, 12, and 24 h, 1 mL samples were collected and centrifuged at 5000 r/min for 5 min. After discarding the supernatant, the bacteria were resuspended in PBS solution with an addition of FDA (fluorescein diacetate, Macklin, F809625, Shanghai, China) dissolved in DMSO to achieve a final concentration of 2 mg/mL. Following incubation at room temperature for approximately 20 min, the bacteria were washed twice with PBS before measuring fluorescence intensity using a multifunctional microplateat an excitation wavelength of 488 nm and an emission wavelength of 530 nm. The experiment was conducted 4 times.

### 2.10 Measurement of intracellular ROS fluorescence intensity

The determination of reactive oxygen species (ROS) was performed following the modified method described by Wei et al. (26). Following the procedure of the reactive oxygen species assay kit (Beyotime Biotechnology, S0033S, Shanghai, China), 1 μL DCFH-DA was incubated with the bacteria at 37°C for 20 min in a constant temperature culture. Subsequently, the bacteria were collected through centrifuged at 5000 r/min for 5 min and washed by PBS for three times. The bacteria were treat with solution of 0 × MIC, 2 × MIC, 4 × MIC, and 8 × MIC at room temperature. Samples of were collected after culturing for 20, 40, and 60 min. Fluorescence measurements were conducted using a multifunctional microplate reader (Thermocycler Varioskan LUX, Shanghai, China), with an excitation wavelength of 488 nm and an emission wavelength of 525 nm. The fluorescence spectrum was plotted and the experiment was repeated for 4 times.

### 2.11 Fluorescent quantitative PCR

GBS bacterial solution cultured to logarithmic phase was supplemented with CHEC at the concentration of 0 × MIC and 8 × MIC, respectively. The bacteria were incubated at 37°C and 150 r/min in a constant temperature shaker for 12 h. The bacteria was obtained by centrifugation and washed three times with sterile PBS. RNA was extracted according to the TaKaRa MiniBEST Universal RNA Extraction Kit (TakaRa, 9,767, China). The extracted total RNA was tested for concentration and purity using an ultra-micro spectrophotometer (Aosen, Hangzhou, China), followed by a PrimeScript^™^ RT reagent Kit with gDNA Eraser (Perfect Real Time) (TakaRa, RR047Q, China) were used to synthesize cDNA at 37°C for 15 min (reverse transcription reaction) and 85°C for 5 s (inactivation reaction of reverse transcriptase). Based on the study conducted by Huo et al. (27), a total of eight virulence genes and 16S rRNA reference genes were selected, and corresponding primers were designed and synthesized by Qingke Biotechnology (Beijing) Co., LTD (Table 1). The qPCR reaction was total 25 μL, including 1 μL of cDNA template, 12.5 μL of 2 × TB GreenPremix Ex Tag ll Fast qPCR, 1 μL 10 μM forward, 1 μL reverse primers, and 9.5 μL of nuclease-free water. Reverse transcription polymerase chain reaction (RT-PCR) was predenatured at 95°C for 30 s, It was extended at 95°C for 5 s and annealed at 60°Cfor 10 s. Forty cycles were performed. Melting was performed at 95°C for 15 s, 60°C for 30 s, and 95°C for 15 s. The effect of CHEC on the expression of GBS virulence genes was calculated according to the CT value, and the relative mRNA expression was calculated using the 2^−ΔΔCt^ method with 16S as the reference gene. The experiment was repeated for 3 times.

**Table 1:** Primer sequences for 8 virulence genes.

Gene	Primer sequences (5′ → 3′)	Target fragment size/bp
*PI*-2b	F:ACACAGGCGACTTATCAACAGGAG	105
	R:AACCTGGCCTCGTTGGATCATTAG	
sip	F:GTATCAGCTCCAGCAGTTCCTGTG	93
	R:TTGTGCTACCGGAACGCTCTTAAC	
hylB	F:GCTTCAACCGCAACTGCAACAG	139
	R:CGCTTGTATGTGACCAGCTTCCAG	
fbsA	F:AGCGTCGTCAACGTGATGCG	149
	R:ACGCTCTAGAACATTGCCTTGGC	
fbsB	F:ACTGCGCAAACTTCTGTCCA	166
	R:GCGAGGTCATTTCCGCAGTT	
CAMP	F:AAGCCCAGCAAATGGCTCAA	156
	R:CTGTCTCAGAGTTGGCACGC	
cpsA	F:ACAACGCTTCACTGTCGAGTCAC	82
	R:AGCTCCTGGCATTGCATATGAAGG	
cpsE	F:AGAAGGTCTGACCACGAAGCATTG	98
	R:ATCCACATTGCACTAGGCGTCAC	
16*S*	F:GCTCACCAAGGCGACGATACATAG	135
	R:GCGTTGCTCGGTCAGACTTCC	

### 2.12 Statistical evaluation method

The resulting data were statistically analyzed using Microsoft Excel. The results were presented as the mean ± standard deviation. One-Way ANOVA and Tukey’s method were employed for analysis and multiple comparisons, respectively, using Graphpad Prism 9.0 software. Fluorescence spectra were plotted using Origin 2021 software. Statistical significance was denoted by * or ^#^ (*p* <0.05), ** or ^##^ (*p* <0.01), *** or ^###^ (*p* <0.001), and **** or ^####^ (*p* < 0.0001).

## 3 Results

### 3.1 The inhibition zone size of CHEC against GBS at different concentrations

The results presented in Table 2 demonstrate the inhibitory potential of CHEC at various concentrations against GBS growth. CHEC exhibited a significant inhibitory effect on GBS at concentrations of 0.5, 1, and 2 mg/mL, resulting in inhibition zone diameters of 10.79 mm, 12.67 mm, and 14.32 mm, respectively. Notably, the inhibitory ability of CHEC increased with higher concentrations. However, it should be noted that the antibacterial efficacy of the three tested concentrations of CHEC was comparatively lower than the observed for the positive control (CTC) at a concentration of 2 mg/mL.

**Table 2:** Inhibition zone diameter of different concentrations of CHEC on GBS.

Treatment	Inhibitory zone diameter (mm)
3% DMSO	0 ± 0
2 mg/mL CTC	26.72 ± 1.26
0.5 mg/mL CHEC	10.79 ± 0.22
1 mg/mL CHEC	12.67 ± 1.76
2 mg/mL CHEC	14.32 ± 0.49

The data in the table are the mean ± standard deviation.

### 3.2 MIC and MBC

GBS gradually became clearer from well 4 in the 96-well plate, indicating that the CHEC concentration from 256 μg/mL had an inhibitory effect on GBS. The results showed that the MIC and MBC of CHEC against GBS were 256 μg/mL and 512 μg/mL, respectively. The 2 × MIC, 4 × MIC, and 8 × MIC were used as 512, 1,024, and 2048 μg/mL, respectively, and were tested as different concentrations in the subsequent tests. The inhibitory effect of CHEC on GBS became evident as the clarity gradually increased from well 4 in the 96-well plate, indicating a concentration-dependent response. The MIC and MBC of CHEC against GBS were determined to be 256 μg/mL and 512 μg/mL, respectively.

### 3.3 The time kill assay of CHEC against GBS

**Figure 1:**
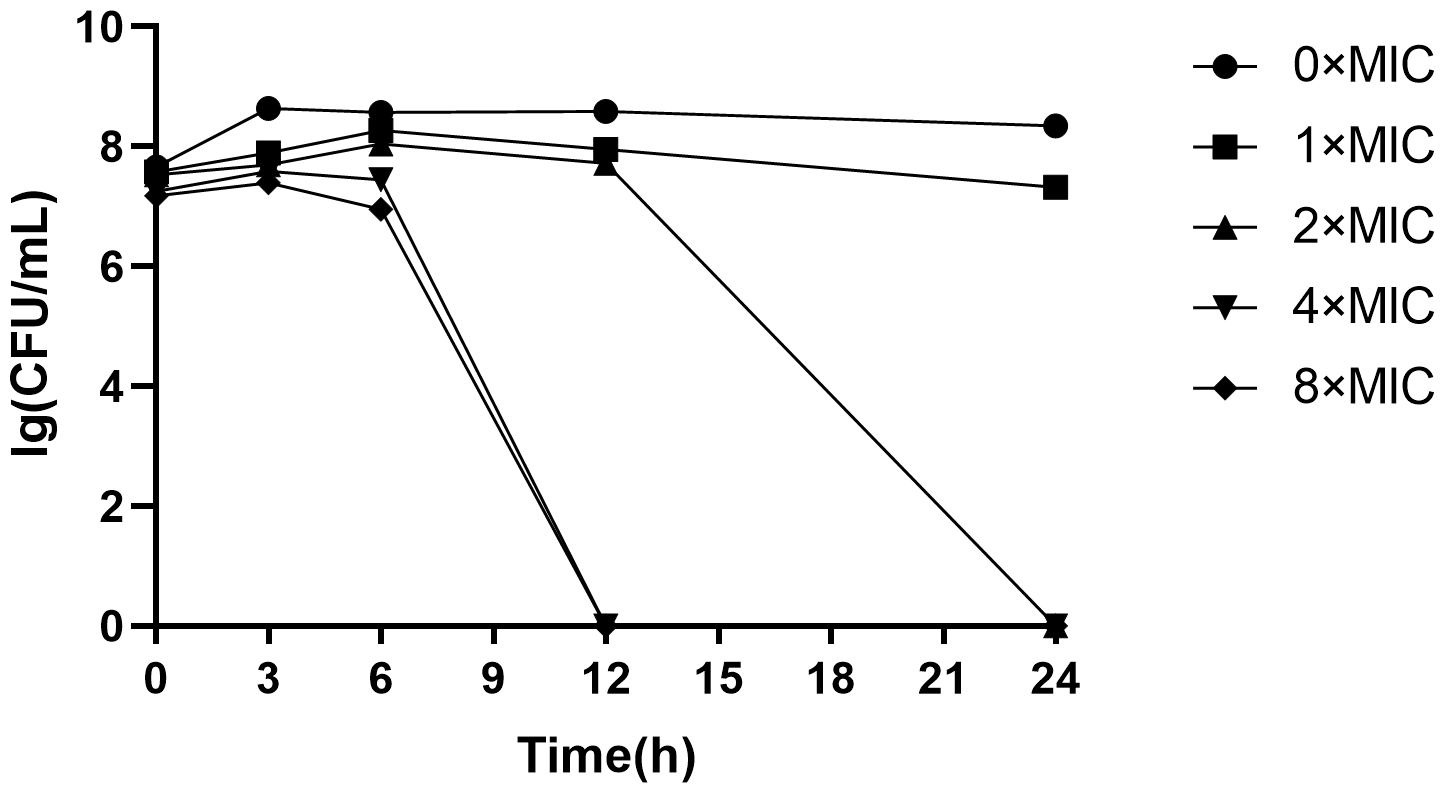
The time-killing effect curve of CHEC against GBS.

As depicted in Figure 1, the total number of colonies in both 4 × MIC and 8 × MIC exhibited a significant decline after 6 h, with no colony formation observed beyond the 12 h mark, indicating that CHEC at concentrations of 4 × MIC and 8 × MIC effectively eradicated GBS within a span of 12 h. The concentration of CHEC at 2 × MIC still allowed for colony formation after a culture period of 12 h but demonstrated bactericidal activity against GBS after a culture duration of 24 h. Conversely, the bactericidal effect of CHEC at 1 × MIC was limited as colonies continued to form even after a culture period exceeding 24 h. Notably, with increasing concentrations of CHEC, there was an associated enhancement in its ability to eradicate GBS *in vitro* in a concentration-dependent manner.

### 3.4 Effects of CHEC on the morphology and ultrastructure of GBS cells

**Figure 2:**
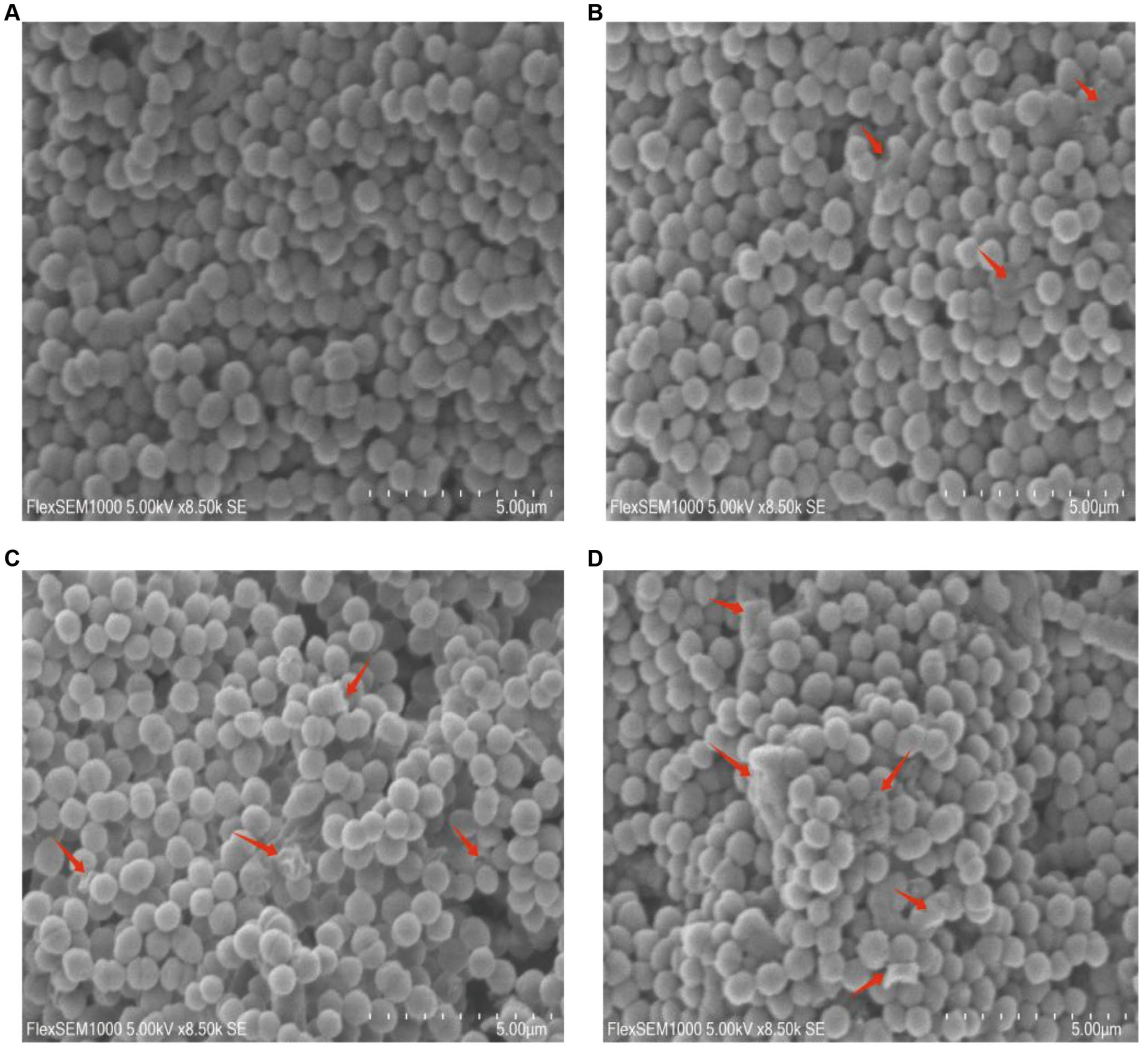
Morphological effects of CHEC on GBS cells. **(A–D)** were the 0 × MIC group treated with DMSO for 24 h and the experimental groups treated with 2 × MIC, 4 × MIC, and 8 × MIC CHEC for 24 h, respectively. Arrows in the figure indicate GBS cells showing significant damage. Arrows in the figure indicate GBS cells showing significant damage.

The scanning electron microscopy (SEM) images revealed distinct morphological alterations in GBS cells. In the 0 × MIC group treated with DMSO for 24 h, the bacteria exhibited a round and plump morphology with a smooth surface (Figure 2A). Conversely, upon exposure to varying concentrations of CHEC for 24 h, the morphology became irregular. Some cells in the 2 × MIC and 4 × MIC groups displayed shrinkage and collapse, while others exhibited cell surface lysis and leakage of cellular fluid (Figures 2B,C). Notably, even in the 8 × MIC group, most of the cell morphology was severely impaired as evidenced by cell adhesion and agglomeration (Figure 2D).

**Figure 3:**
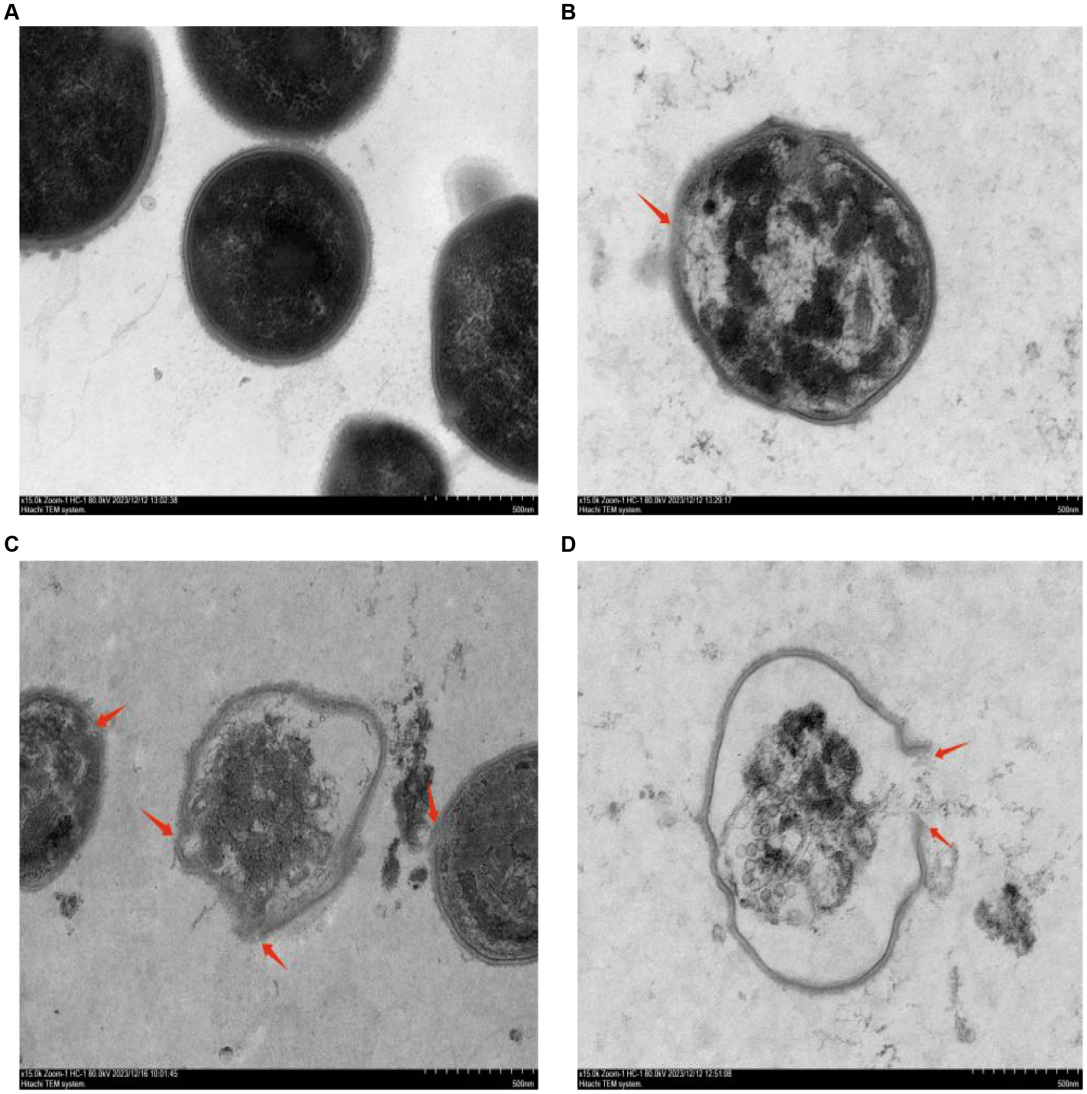
Effect of CHEC on the ultrastructure of GBS. **(A–D)** were the 0 × MIC group treated with DMSO for 24 h and the experimental groups treated with 2 × MIC, 4 × MIC, and 8 × MIC CHEC for 24 h, respectively. Arrows in the figure indicate GBS cells showing significant damage. Arrows in the figure indicate GBS cells showing significant damage.

The destruction process of GBS internal structure by different concentrations of CHEC was observed using TEM (Figure 3). The results demonstrated that GBS bacteria at 0 × MIC exhibited intact cell wall and membrane, dense and uniformly distributed contents, as well as preserved cell morphology (Figure 3A). Following CHEC treatment for 24 h, the majority of bacteria treated with 2 × MIC displayed normal spherical shape; however, a small proportion started to undergo lysis, resulting in separation of cytoplasm from the cell wall, cellular shrinkage and deformation, decreased electron density, and dissolution and blurring of the cell membrane and wall (Figure 3B). Conversely, higher concentrations led to wrinkling and rupture of GBS cells treated with CHEC at both 4 × MIC and 8 × MIC levels. These cells exhibited incomplete cell walls and membranes along with massive content leakage (Figures 3C,D). SEM and TEM findings collectively indicated that CHEC effectively disrupted both internal and external morphological structures of GBS *in vitro* while compromising cellular integrity ultimately leading to disintegration and death.

### 3.5 Effects of CHEC on the AKP activity of GBS

**Figure 4:**
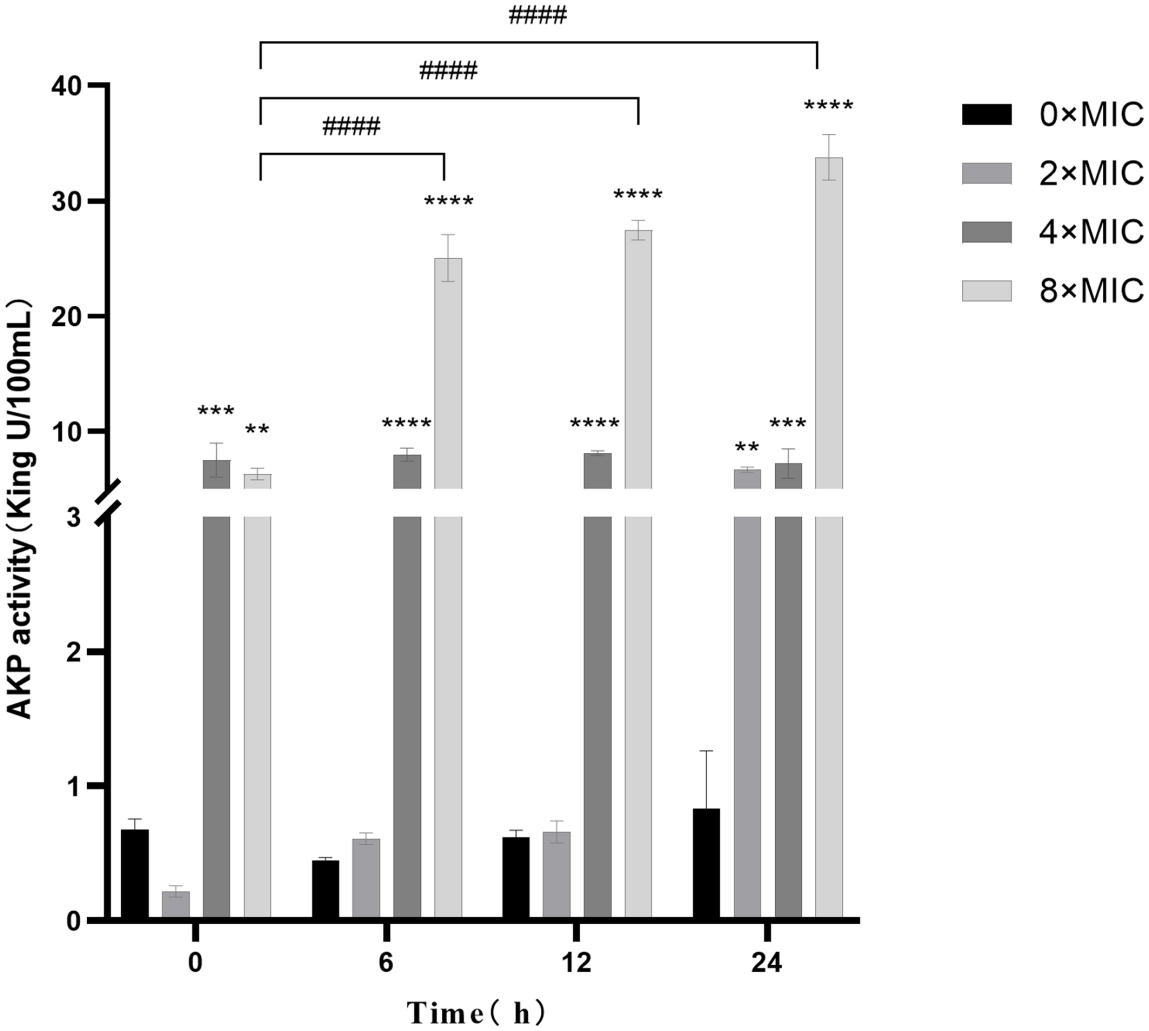
Effect of CHEC on AKP in GBS. 0 × MIC for comparison in the same period **p < 0.01, ***p < 0.001, ****p < 0.0001. The 8 × MIC at 0 h was used as a comparison in different time periods, ^####^*p* < 0.0001.

The results are presented in Figure 4, illustrating the cultivation of bacterial suspensions at various concentrations and the subsequent varying degrees of increased AKP activity. Notably, AKP activity at 2 × MIC and 8 × MIC exhibited a positive correlation with culture time, particularly when combined with higher CHEC concentrations and longer culture durations. This observation suggests that the high concentration of CHEC may lead to cell wall disruption and subsequent leakage of AKP from the cells. However, it is worth mentioning that the AKP activity at 4 × MIC and 8 × MIC was significantly higher than that observed at 0 × MIC. There was a significant difference in AKP activity between samples treated with 2 × MIC and 0 × MIC after a duration of 24 h (*p* < 0.01). Furthermore, the AKP activity of 8 × MIC was significantly higher than that of 0 × MIC at 6, 12, 24 h (*p* < 0.0001). These findings collectively demonstrate that different concentrations of CHEC effectively induce cell wall damage, resulting in intracellular AKP leakage and subsequent elevation in extracellular AKP activity.

### 3.6 Effects of CHEC on Na^+^, K^+^, and Ca^2+^, Mg^2+^-ATP activity in GBS

**Figure 5:**
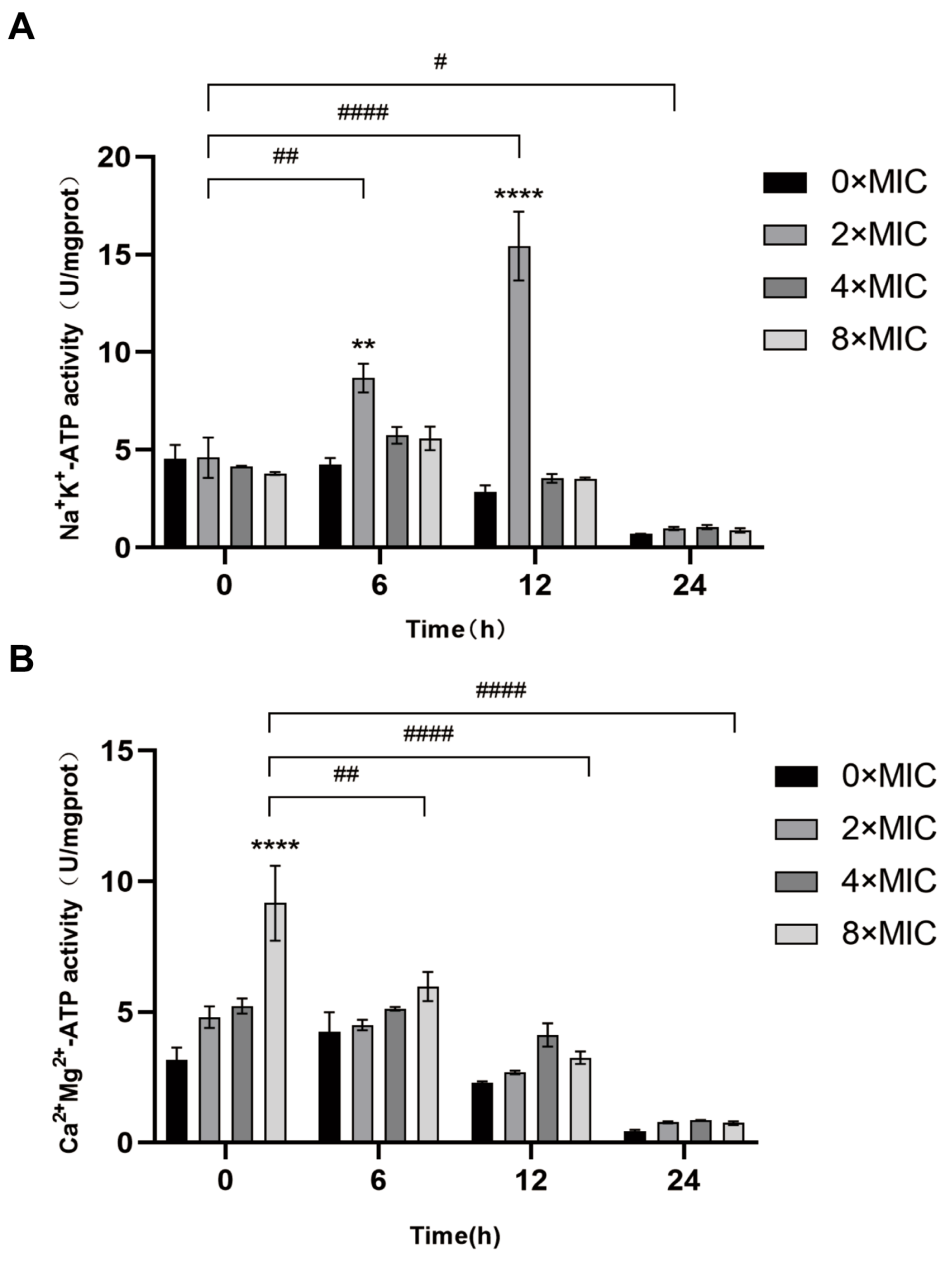
Effect of CHEC on Na^+^, K^+^, and Ca^2+^Mg^2+^-ATP activities of GBS. **(A)** Determination of Na^+^, K^+^-ATP activity of GBS by CHEC. **p < 0.01 and ***p < 0.001. The 2 × MIC at 0 h in different time periods was used for comparison. ^#^*p* < 0.05, ^##^*p* < 0.01, ^####^*p* < 0.0001. **(B)** Determination of Ca^2+^, Mg^2+^-ATP activity of GBS by CHEC. ****p < 0.0001. The 8 × MIC at 0 h was used as a comparison in different time periods, ^##^*p* < 0.01 and ^####^*p* < 0.0001.

As shown in Figure 5, the Na^+^, K^+^-ATP activity remained at a comparable level at 0 h when treated with different concentrations of CHEC, exhibiting no significant difference. However, the Ca^2+^, Mg^2+^-ATP activity displayed a similar trend at both 0 h and 6 h, with the highest ATP activity observed at 8 × MIC at 0 h. The Na^+^, K^+^-ATP activity in the CHEC treatment group started to increase after 6 h of culture, reaching its peak values at 4 × MIC and 8 × MIC. At 12 h, there was a significant increase in Na^+^ K^+^ activity for the concentration of 2 × MIC compared to that of 0 × MIC (*p* < 0.0001). Both Na^+^, K^+^-ATP and Ca^2+^, Mg^2+^-ATP exhibited a declining trend after culturing for more than 24 h. This decline may be attributed to prolonged culture time leading to cell membrane damage and subsequent extracellular permeability of Na^+^, K^+^, Ca^2+^, and Mg^2+^. Consequently, ATP becomes inactive over an extended period and is not fully detected. The findings indicate that CHEC enhances the permeability of Na^+^, K^+^, Ca^2+^, and Mg^2+^ ions, thereby increasing extracellular ATP levels. Moreover, CHEC effectively disrupts the integrity of GBS cell membranes.

### 3.7 Effect of CHEC on membrane permeability of GBS cells

**Figure 6:**
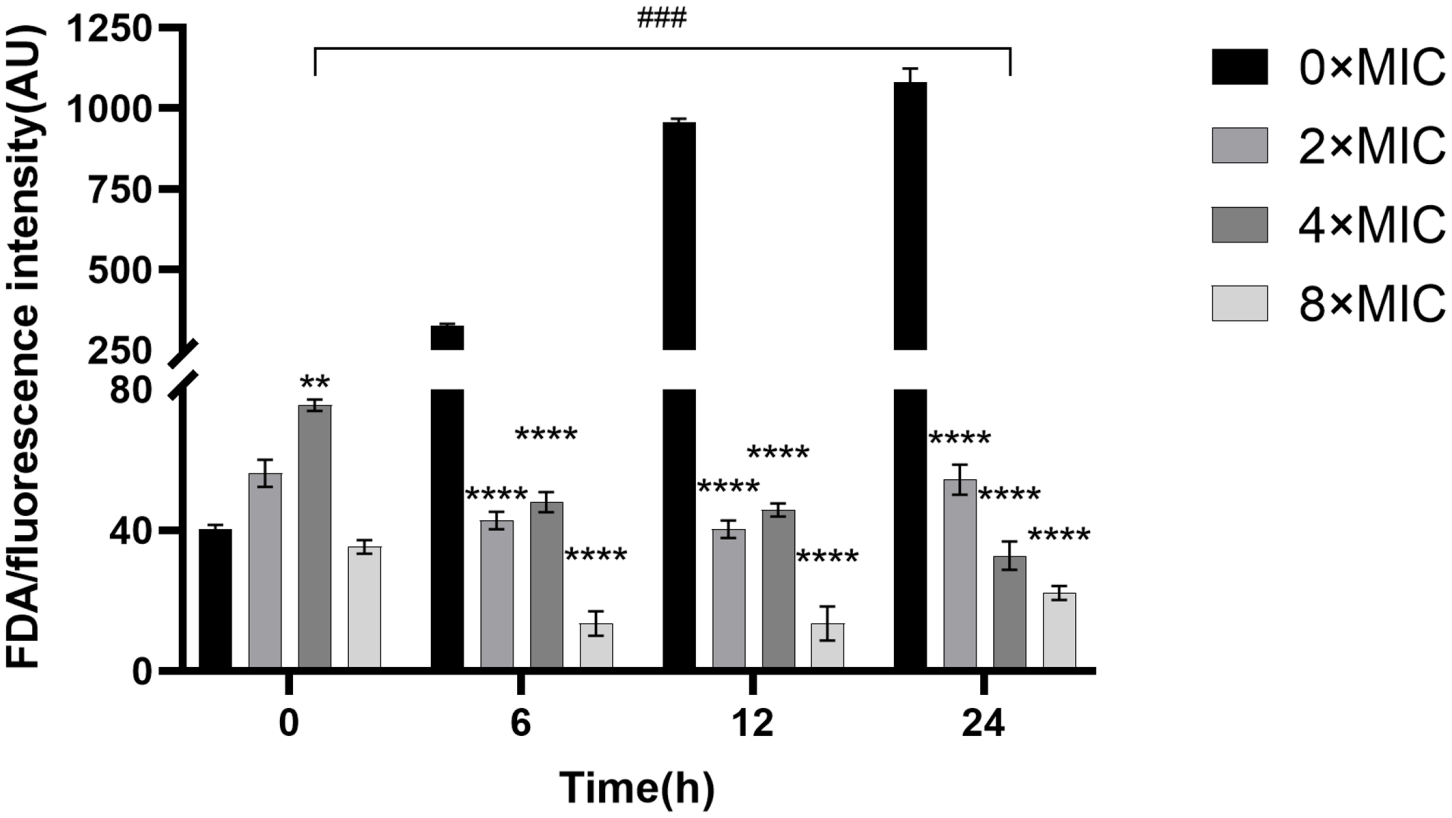
Fluorescein diacetate staining experiment to study the effect of CHEC on membrane permeability of GBS. *p < 0.05 and ****p < 0.0001. The 4 × MIC at 0 h was used as a comparison in different time periods, ^###^*p* < 0.001.

As shown in Figure 6, 2 × MIC, 4 × MIC, and 8 × MIC fluorescein began to decrease from 6 h onwards. Notably, the fluorescence intensity of the control group 0 × MIC was significantly higher than that of the groups treated with 8 × MIC, 4 × MIC, and 2 × MIC at both 12 and 24 h (*p* < 0.0001). At 24 h, the fluorescence intensity 2 × MIC was found to be higher than that of 4 × MIC and the 8 × MIC. Furthermore, the fluorescence intensity of 0 h 4 × MIC was significantly different from that of 24 h 4 × MIC (*p* < 0.001). These findings demonstrate that different concentrations of CHEC can effectively disrupt GBS cell membranes leading to loss of fluorescein from bacterial cells and subsequent reduction in FDA fluorescence intensity.

### 3.8 Effects of CHEC on the intracellular ROS content of GBS

**Figure 7:**
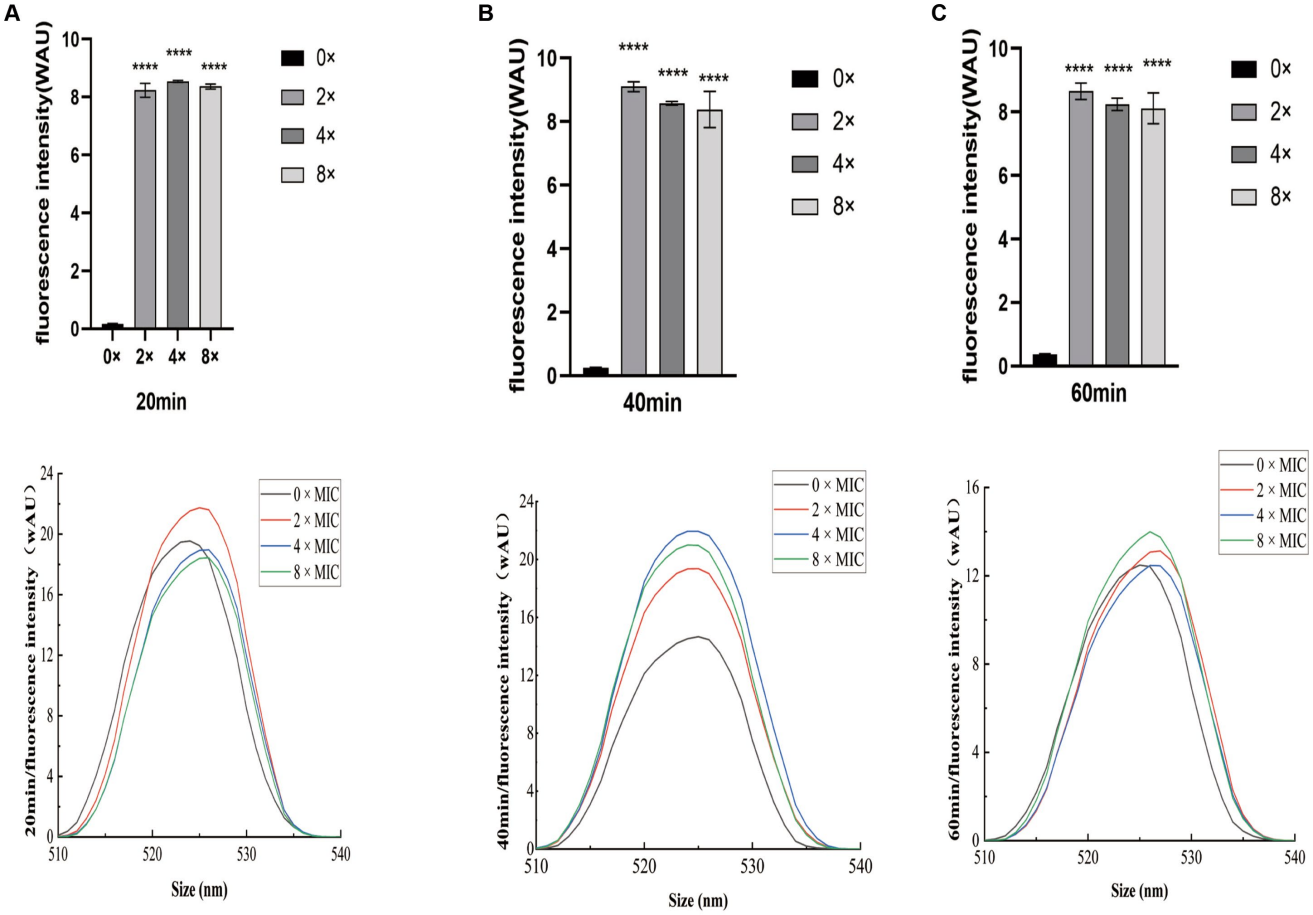
Effect of CHEC on intracellular ROS fluorescence intensity in GBS **(A)** 20 min, **(B)** 40 min, and **(C)** 60 min. ****p < 0.0001.

The intracellular ROS fluorescence intensity results following CHEC treatment of GBS are presented in Figure 7. The ROS content at 0 × MIC exhibited a slowly increase over time, while the ROS content at 8 × MIC, 4 × MIC, and 2 × MIC showed a sharp increase with culture time. Moreover, the ROS content at 8 × MIC, 4 × MIC and 2 × MIC was significantly different from that at 0 × MIC at 20 min, 40 min and 60 min (*p* < 0.0001). At both the 40 min and 60 min marks, the highest ROS content was observed for the concentration of 2 × MIC followed by those of 4 × MIC and then by those of 8 × MIC. This suggests that this particular concentration is most effective in inducing ROS production. Additionally, analysis of fluorescence spectra revealed a similar trend to that observed for fluorescence intensity. These findings indicate that CHEC at various concentrations can induce oxidative damage to bacteria and stimulate significant production of ROS by GBS.

### 3.9 Effects of CHEC on virulence genes

**Figure 8:**
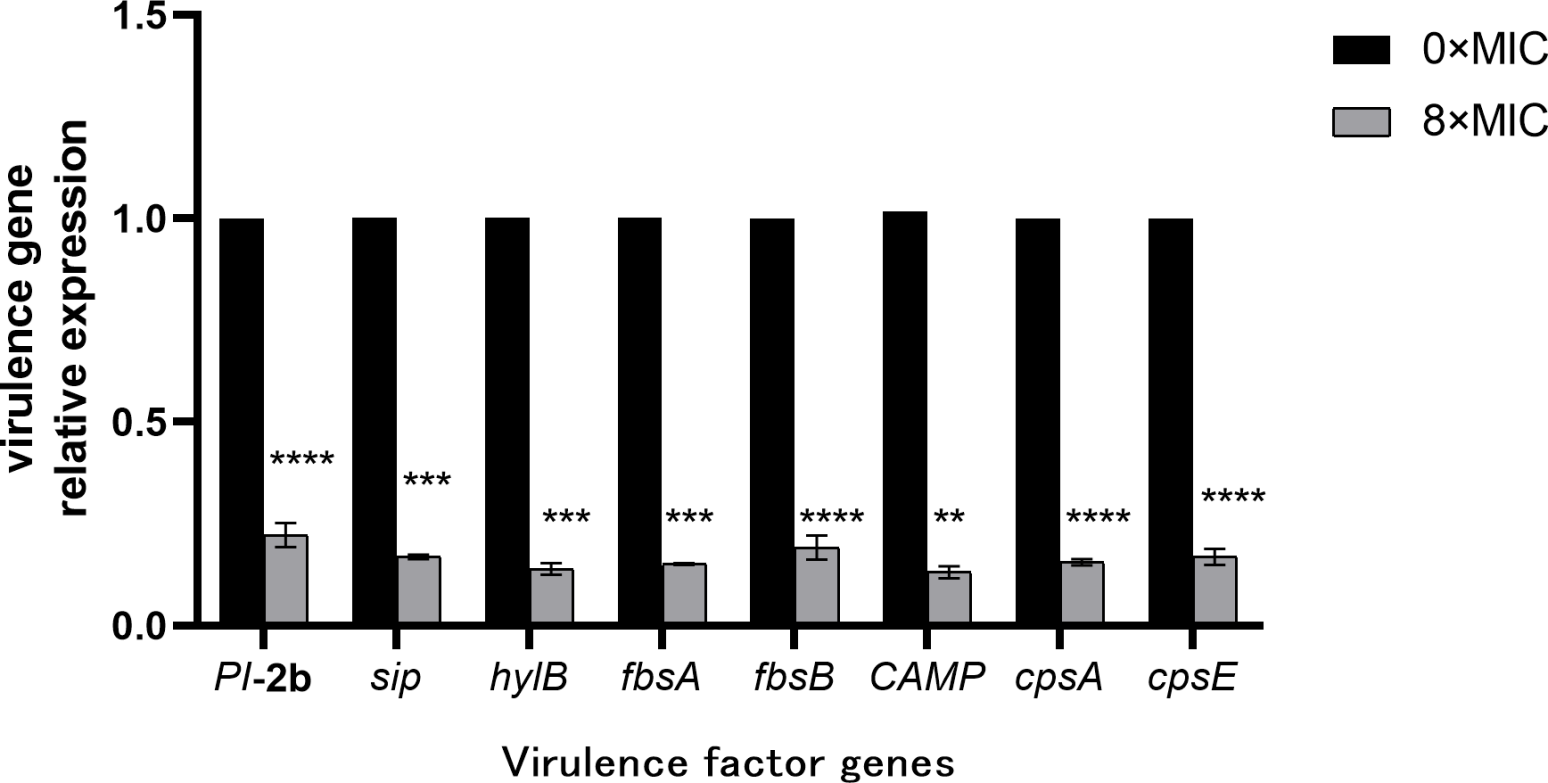
Effect of CHEC on the expression of eight virulence-related genes of GBS. **p < 0.01, ***p < 0.001, and ****p < 0.0001.

The effect of CHEC on gene expression after GBS treatment was verified using qRT-PCR. As shown in Figure 8, the gene expressions of fimbrial protein (*PI*-2b), fibrinogen binding protein (*fbsA* and *fbsB*) and surface immune-related protein (*sip*), which were closely related to the adhesion and colonization of GBS, were significantly down-regulated in the 8 × MIC CHEC treatment group. The expression of CAMP factor (*CAMP*) and hyaluronidase (*hylB*) genes, which play important roles in the invasion of GBS, were significantly decreased. In addition, the expression of capsular polysaccharide related gene (*cpsA*, *cpsE*), which is related to bacterial immune evasion, was also significantly decreased (*p* < 0.0001).

## 4 Discussion

CHE is a naturally occurring benzophenidine alkaloid with diverse biological and pharmacological activities, including antibacterial, antitumor, and anti-inflammatory effects (28). Wang et al. (29) demonstrated that the combination of CHE with conventional antibiotics holds promise as a natural product for treating multidrug-resistant bacterial infections, particularly against Gram-positive bacteria. In this study, we investigated the antimicrobial activity and mechanism of action of CHE derived from *Macleaya cordata* (MC) against *Streptococcus agalactiae* (GBS). The inhibition zone diameter of 2 mg/mL CHEC was measured to be 14.32 mm. The MIC and MBC values were determined to be 256 μg/mL and 512 μg/mL, respectively. However, the active compounds derived from other plant extracts exhibited enhanced antibacterial efficacy against GBS. Torres et al. (30) isolated and identified three active compounds from garlic that demonstrated antimicrobial activity against GBS, with MICs of 5,410 μg/mL, 4,600 μg/mL, and 160 μg/mL, respectively. Pang et al. (31) assessed the antibacterial potential against GBS in a collection of 2,443 natural products and observed MIC ranging from 4 to 256 μg/mL and MBC ranging from 4 to 64 μg/mL. These results may stem from variations in tested strains’ susceptibility to the drug or differences in antimicrobial activities among different fractions or concentrations of CHE extracts. The discrepancies in the aforementioned results may arise from variations in the strains employed during testing, divergent susceptibility to the drug, dissimilar CHE extracts exhibiting distinct antimicrobial activities across different fractions, as well as varying concentrations of CHE that exert disparate effects on the bacteria. The results of time-kill curve in this study showed that CHEC at 8 × MIC and 4 × MIC concentrations could kill GBS within 12 h, while the bacteria could be killed within 24 h after treatment with 2 × MIC. Zhang et al. (32) also reported concentration-dependent antibacterial activity for Terpinen-4-ol against GBS; bacteria treated with a concentration equal to 2 × MIC were killed within 12 h according to their time-killing curve analysis. The *in vitro* antimicrobial activity results of the present assay were similar to those of these studies, indicating that CHEC had a good inhibitory effect on GBS.

Previous studies have demonstrated that the bactericidal effects of various plant extracts primarily involve the disruption of cell morphology and microstructure, consequently impacting bacterial functionality. The alterations in bacteria can be directly observed using scanning electron microscopy (SEM) and transmission electron microscopy (TEM) techniques (33). Peng et al. (20) observed through TEM analysis that treatment with 78 μg/mL berberine resulted in the rupture of bacterial cell membranes, unequal cell division, and severe damage to bacterial cells. Similarly, Eliane et al. (34) found that copaiba oil treatment induced morphological and ultrastructural changes in GBS, as evidenced by SEM and TEM results indicating cytoplasmic content leakage due to disrupted cell walls and reduced electron density. These findings align with those obtained from our study where SEM and TEM observations revealed significant microstructural modifications in GBS following treatment with different concentrations of CHEC including destruction of the cell membrane and wall, cytolysis, reduction in cytoplasm volume, as well as leakage of cellular contents. Therefore, it suggest that CHEC exerts its antibacterial effect on GBS by disrupting the cellular structure leading to loss of cytoplasmic material.

The protective effect of the cell wall can maintain the intrinsic morphology of the bacteria and enable the cells to survive in a relatively hypotonic environment. Gram-positive bacteria possess a thick and dense layer of peptidoglycan and teichoic acid, which constitutes a unique and crucial component structure (35). Alkaline phosphatase (AKP) is located between the bacterial cell wall and membrane, making it an indicator of cell wall permeability. When the cell wall is damaged, AKP leakage occurs, leading to elevated extracellular AKP activity that indirectly reflects the integrity of the bacterial cell wall (36). In this study, we observed that different concentrations of CHEC effectively disrupted the cell wall, resulting in intracellular AKP leakage and increased extracellular AKP activity. This finding aligns with He et al. (37) report on antibacterial activity against *Staphylococcus aureus* (SA), methicillin-resistant *Staphylococcus aureus* (MRSA), and extended-spectrum β-lactamase *Staphylococcus aureus* (ESBLs-SA) using CHE isolated from *Toddalia asiatica* roots. ATP serves as the donor that maintains normal cellular energy metabolism and exchange. It has been utilized to demonstrate common biological alterations in response to antimicrobial-induced damage to cell membranes. In order to assess the impact of CHEC on GBS cell membrane integrity, this study employed kits for detecting the release of Na^+^, K^+^ and Ca^2+^, Mg^2+^-ATP ions. The findings revealed that CHEC effectively disrupted the integrity of GBS cell membrane, leading to increased permeability of Na^+^, K^+^, Ca^2+^, and Mg^2+^, consequently resulting in elevated extracellular ATP activity. Similar to our study, Wu et al. (38) also discovered that fungal defensin-derived peptide P2 induced ATP release in GBS cells. FDA is a permeable molecule capable of penetrating living cell membranes. Once inside the cell body, FDA undergoes hydrolysis into fluorescein which cannot penetrate the cell membrane itself but causes green fluorescence within the cell, thereby enabling determination of bacterial cell membrane destruction using FDA fluorescent dye staining technique. In our study, the fluorescence intensity of the control group 0 × MIC was significantly higher than that of the groups treated with CHEC. Similar to our study, Renata et al. (39) employed a staining kit to evaluate bacterial viability in GBS treated with eugenol.

Reactive oxygen species (ROS) was known as a significant cellular activity indicator. While low levels of ROS can function as intracellular messengers, high levels can cause oxidation with intracellular components such as lipids, proteins, and nucleic acids, ultimately disrupting bacterial cellular structure and resulting in bacterial death. To investigate the accumulation of ROS in GBS cells treated with varying concentrations of CHEC, intracellular ROS fluorescence intensity was measured after 20 min, 40 min, and 60 min of CHEC treatment on GBS cells, followed by plotting the fluorescence spectra. The results of fluorescence intensity and fluorescence spectrum demonstrated that different concentrations of CHEC could induce GBS to generate a substantial amount of reactive oxygen species (ROS), leading to oxidative damage in the bacteria. This process is detrimental to cells and leads to cell demise. Gu et al. (40) used a DCFH-DA fluorescent probe to quantify intracellular ROS levels and observed that Sanguinarine induced DNA damage and apoptosis, producing a bactericidal effect by up-regulating ROS in *Staphylococcus aureus*. Our findings provided evidence supporting the association between CHEC’s bactericidal efficacy and increased ROS levels.

GBS harbors a diverse array of virulence factors that contribute to bacterial adhesion, evasion of immune mechanisms, invasion of the host leading to tissue damage, and other pathogenic processes (41). The *fbsB* and *PI*-2b genes primarily mediate adhesion function and participate in biofilm formation. Additionally, *CAMP* can influence bacterial invasiveness. These virulence determinants facilitate GBS colonization within the host, potentially resulting in disease manifestation under specific conditions. In this study, as the concentration of CHE increased from 0 × MIC to 8 × MIC, there was a significant dose-dependent reduction in mRNA expression levels for all eight virulence genes examined. Consistent with our findings, Huo et al. (27) reported that matrine treatment also led to a substantial decrease in mRNA expression levels for key virulence genes such as *fbsB* and *PI*-2b in GBS. Therefore, our results suggest that CHE has the potential to downregulate pathogenic gene expression.

## 5 Conclusion

In summary, CHEC exhibited promising antibacterial activity against GBS. The primary antibacterial mechanism of CHEC may be attributed to its capacity to disrupt the cell wall and cell membrane of GBS, leading to enhanced membrane permeability and consequent leakage of cellular contents into the extracellular space. Simultaneously, CHEC might induce oxidative damage in GBS by generating ROS and downregulating mRNA expression levels of key virulence genes. By impairing cell structure and interfering with intracellular metabolism, CHEC holds potential as an antimicrobial agent against GBS and further investigations are necessary to elucidate additional molecular mechanisms.

## Data availability statement

The raw data supporting the conclusions of this article will be made available by the authors, without undue reservation.

## Author contributions

JX: Conceptualization, Funding acquisition, Project administration, Supervision, Writing – review & editing. QP: Conceptualization, Data curation, Formal analysis, Methodology, Visualization, Writing – original draft. RW: Formal analysis, Validation, Writing – original draft. YG: Validation, Writing – original draft. LH: Investigation, Supervision, Writing – original draft. XD: Visualization, Writing – original draft. GT: Data curation, Writing – original draft. DH: Funding acquisition, Methodology, Project administration, Resources, Writing – original draft.
