# Supplementary figures and images for: Antibacterial activity and mechanism of chelerythrine against Streptococcus agalactiae
Source: Front Vet Sci. 2024 Jun 14;11:1408376. doi: 10.3389/fvets.2024.1408376 (PMC11212505; doi:10.3389/fvets.2024.1408376)

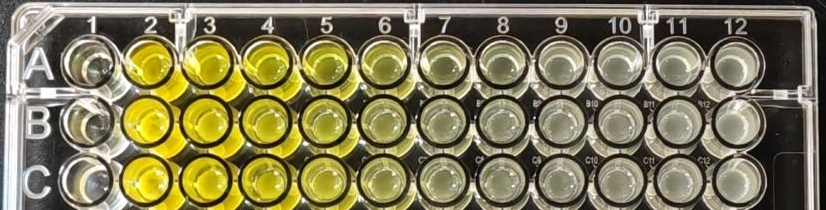

Supplement: Supplementary file 1 [file Data_Sheet_1.ZIP › Figure1-MIC/MIC images/MIC images .jpg]

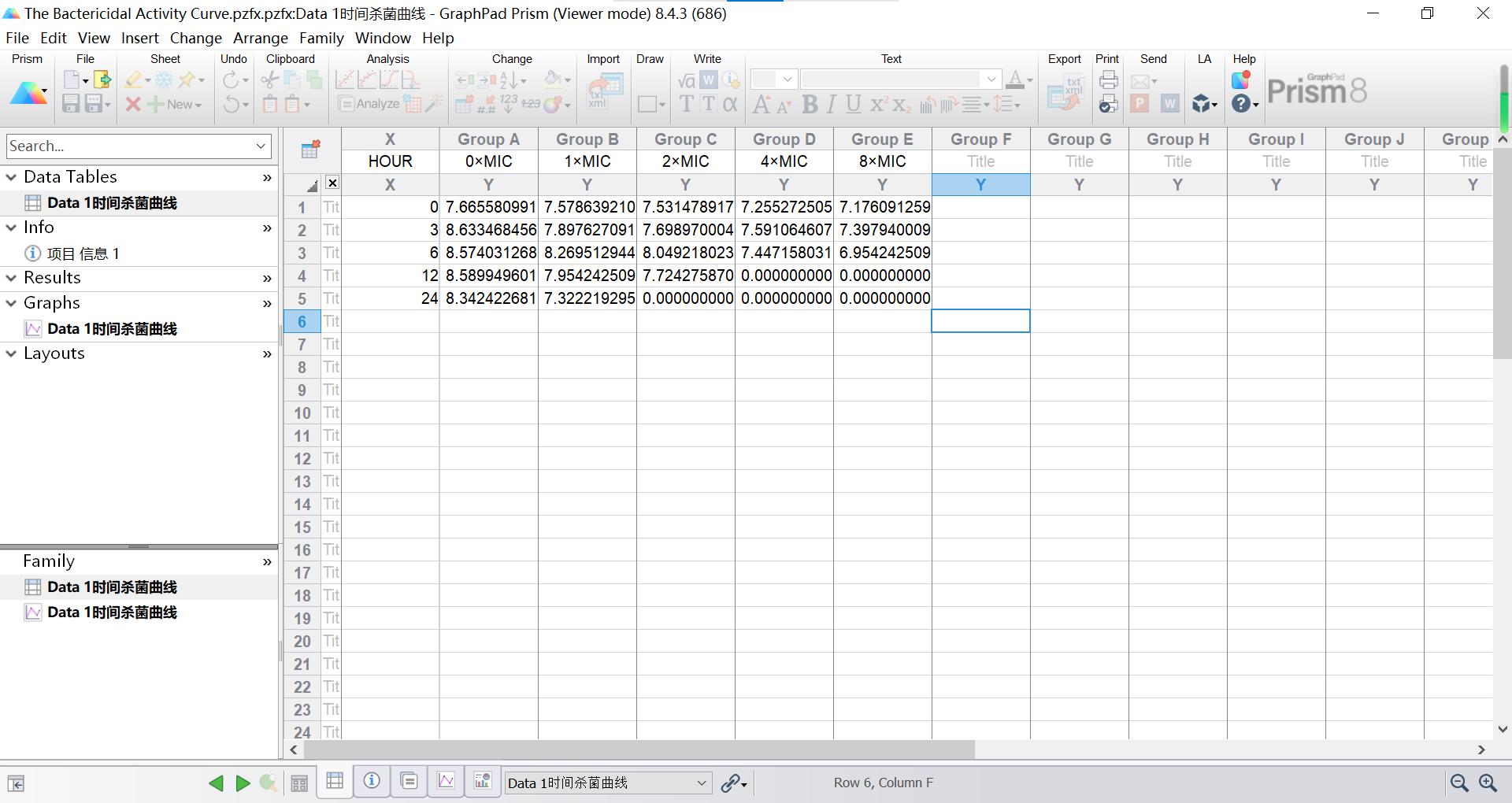

Supplement: Supplementary file 1 [file Data_Sheet_1.ZIP › Figure1-MIC/screenshots of GraphPad Prism 8.0/Time-The Bactericidal Activity Curve..jpg]

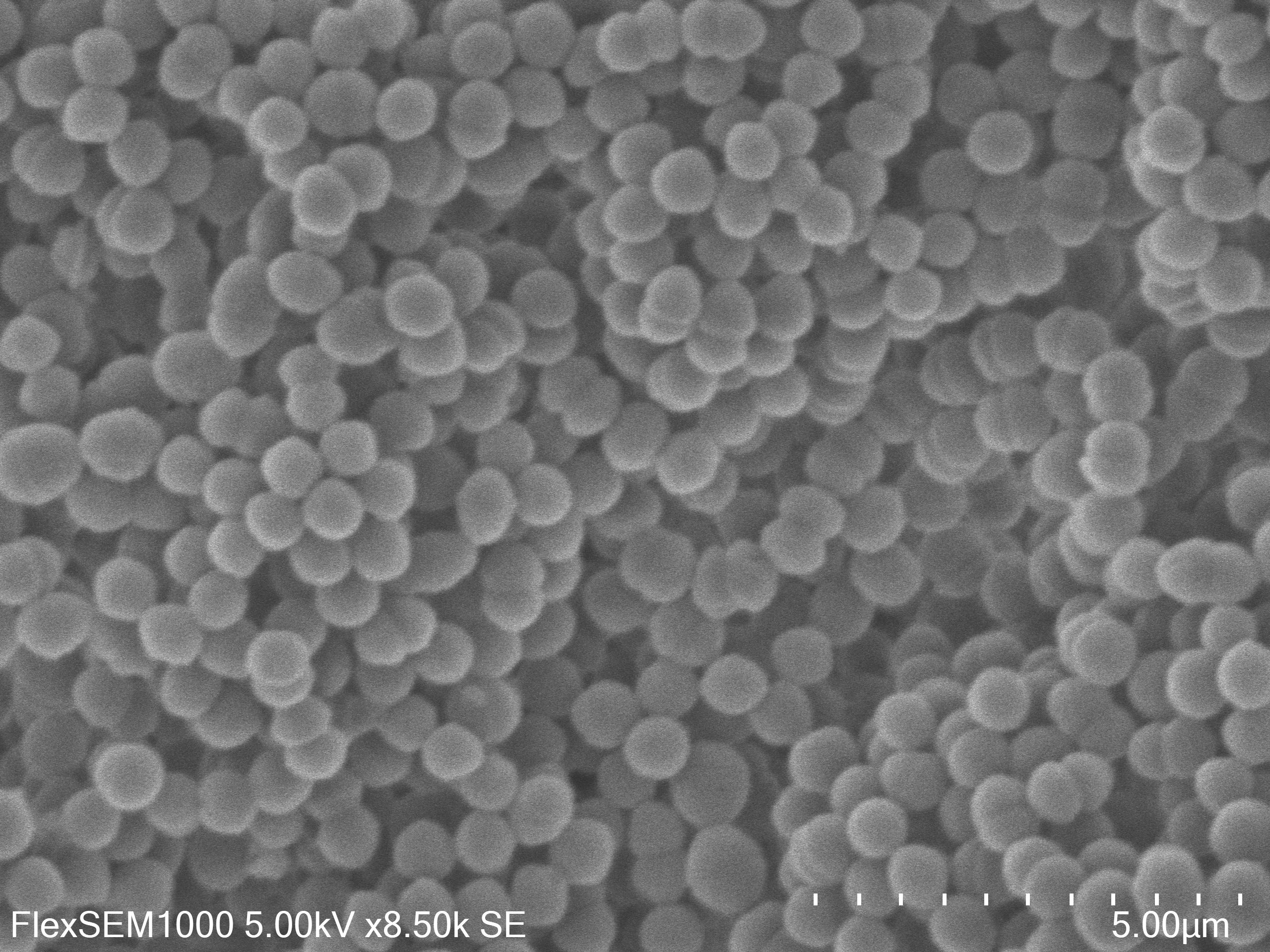

Supplement: Supplementary file 1 [file Data_Sheet_1.ZIP › Figure2-SEM/0×-1.jpg]

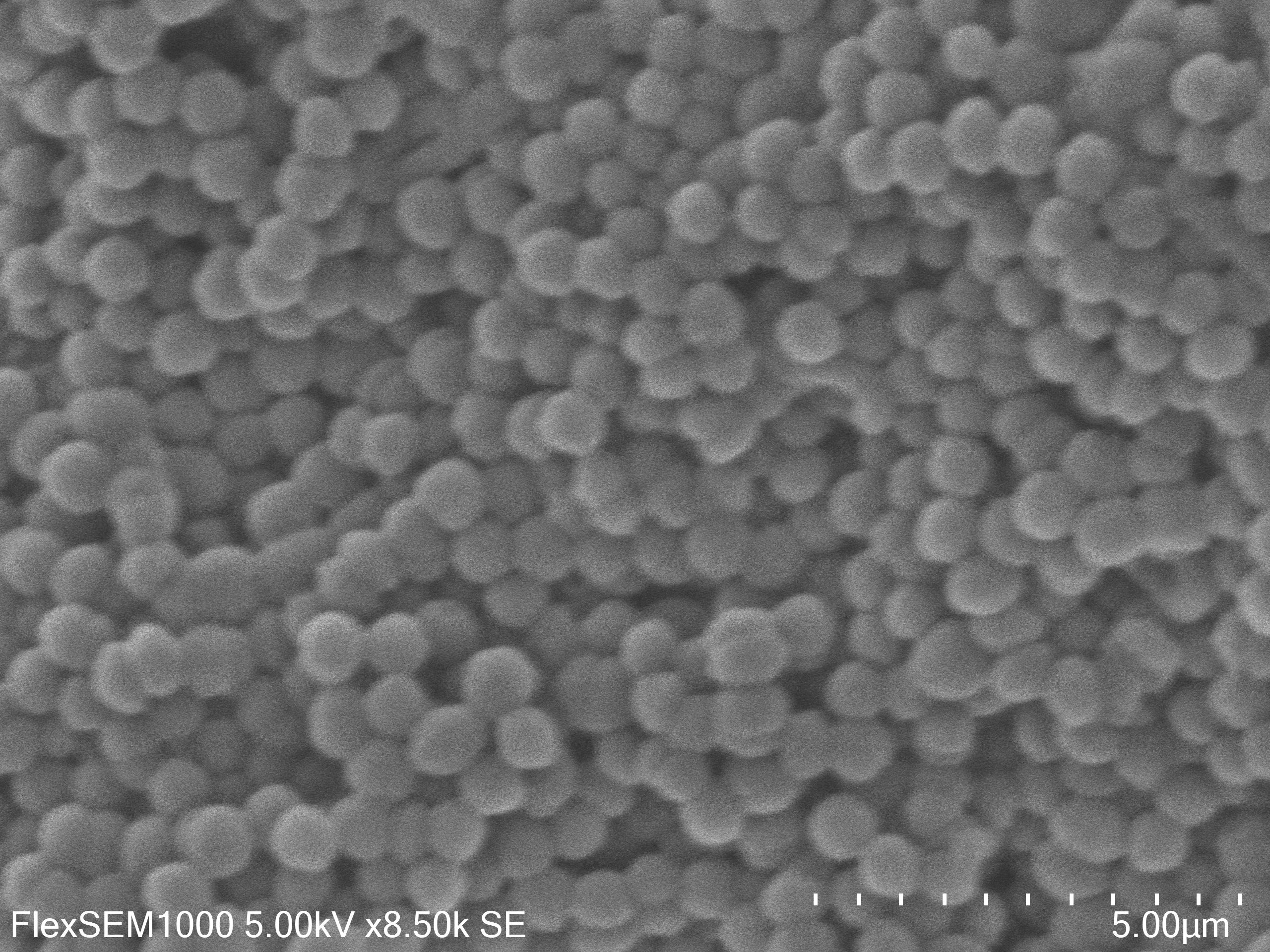

Supplement: Supplementary file 1 [file Data_Sheet_1.ZIP › Figure2-SEM/0×-2.jpg]

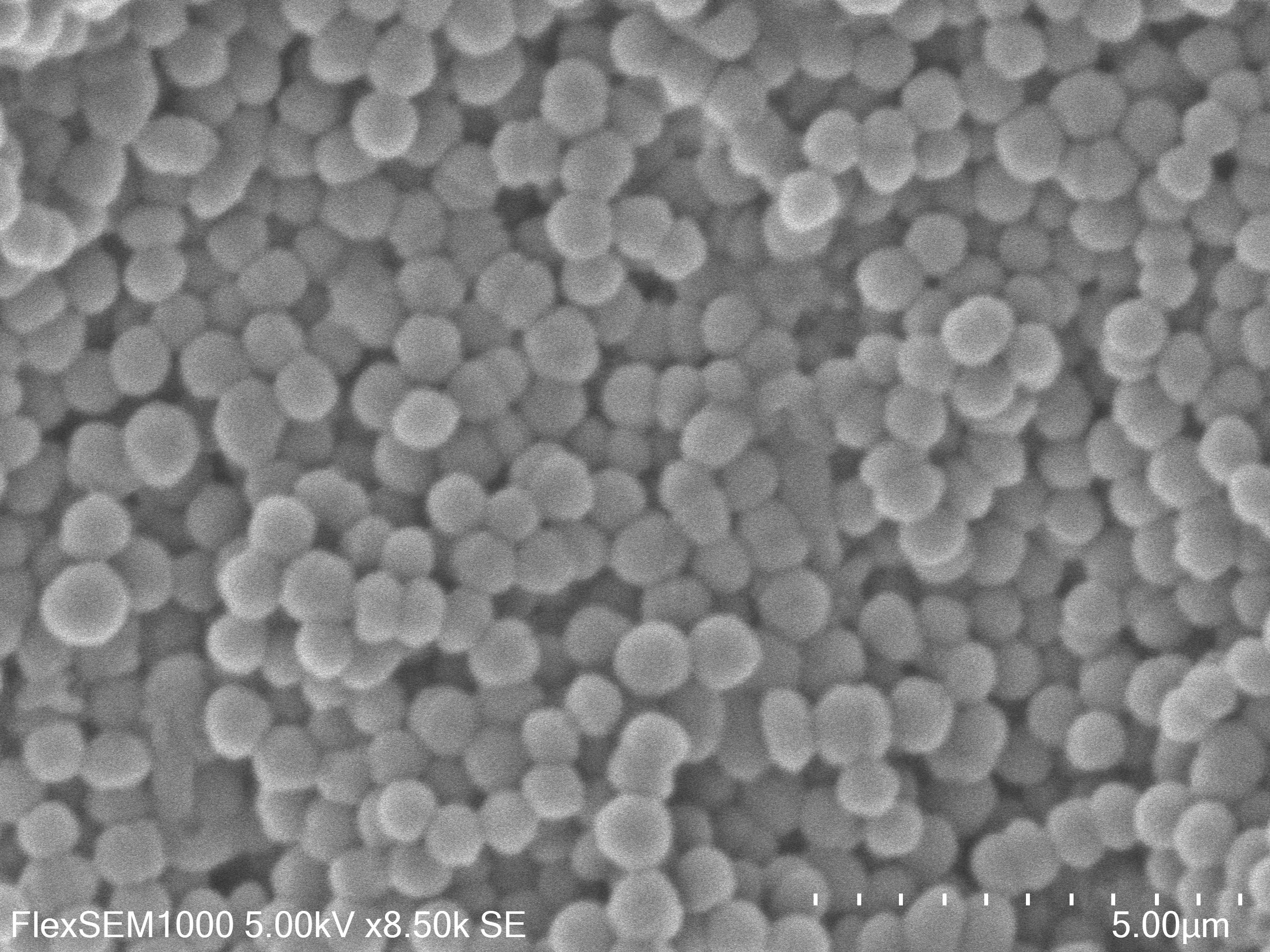

Supplement: Supplementary file 1 [file Data_Sheet_1.ZIP › Figure2-SEM/0×-3.jpg]

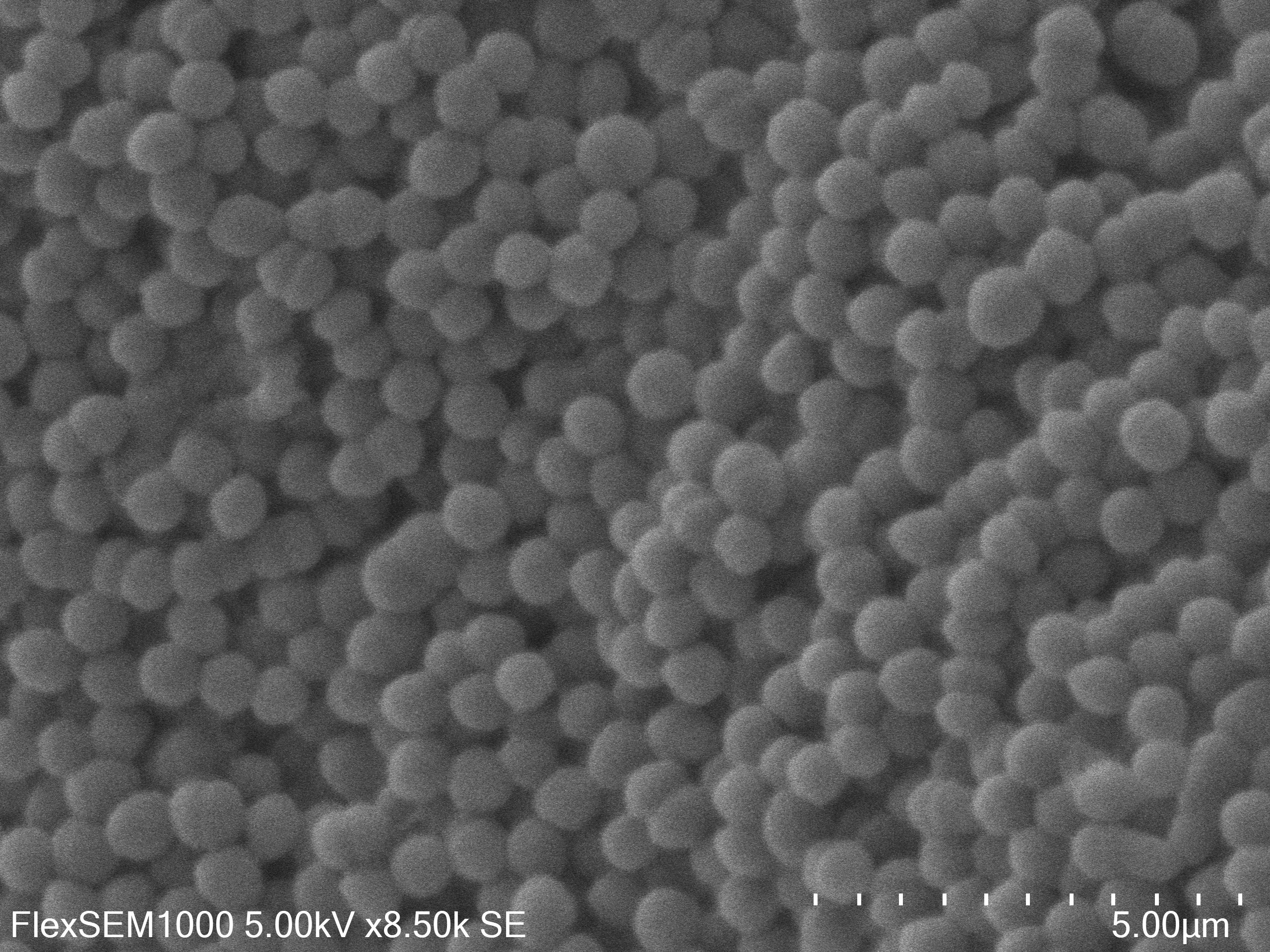

Supplement: Supplementary file 1 [file Data_Sheet_1.ZIP › Figure2-SEM/2×-1.jpg]

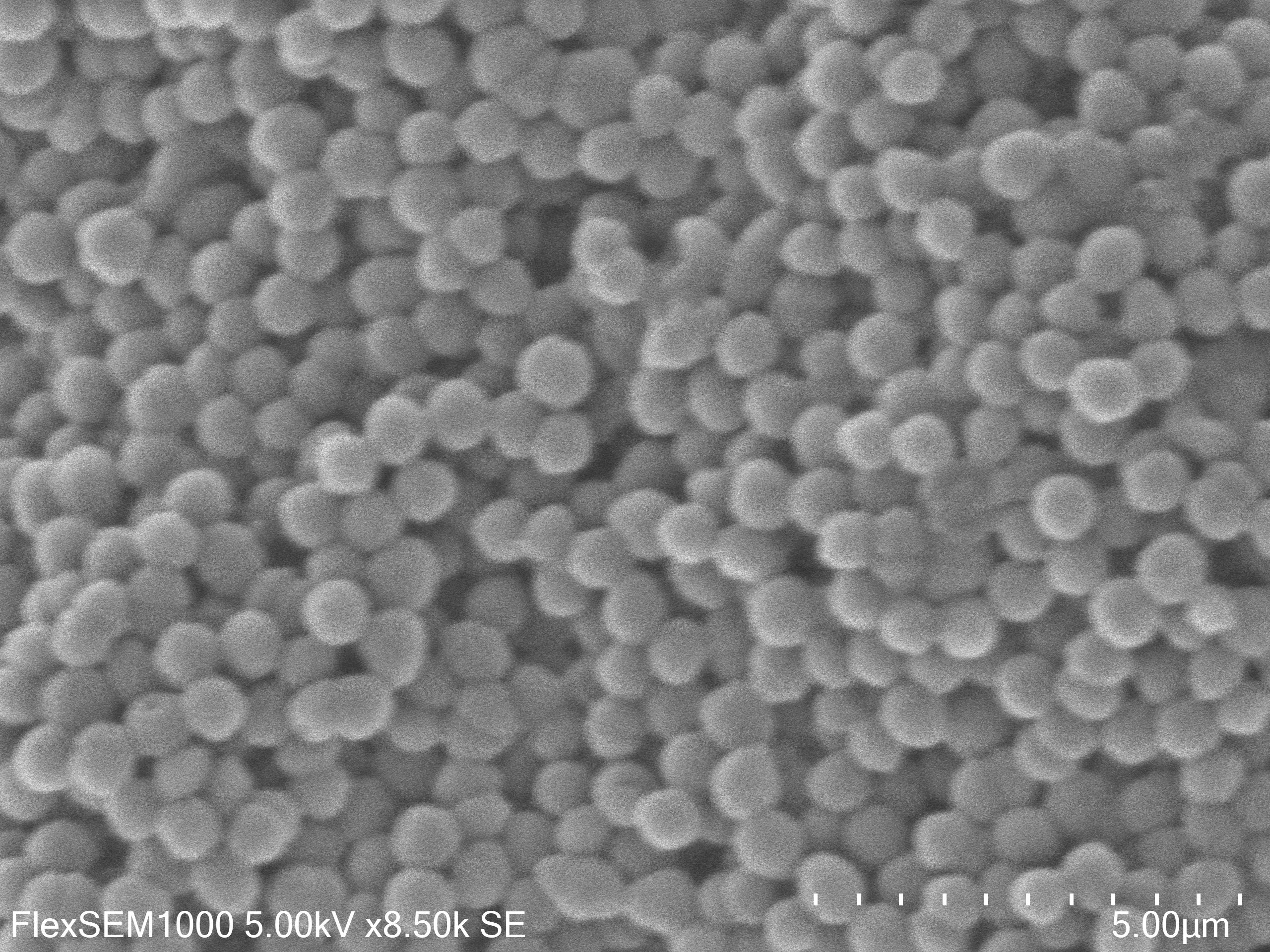

Supplement: Supplementary file 1 [file Data_Sheet_1.ZIP › Figure2-SEM/2×-2.jpg]

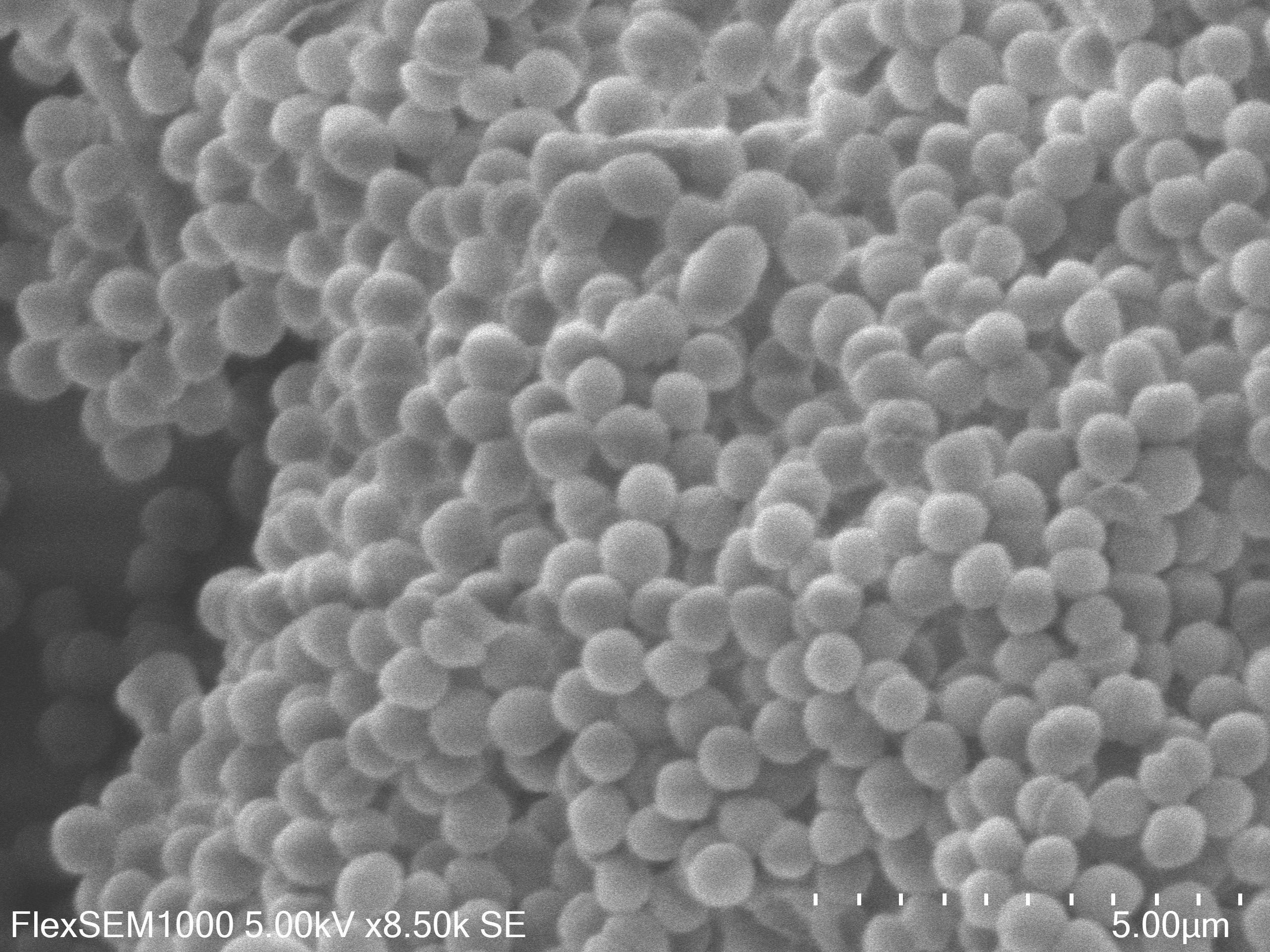

Supplement: Supplementary file 1 [file Data_Sheet_1.ZIP › Figure2-SEM/2×-3.jpg]

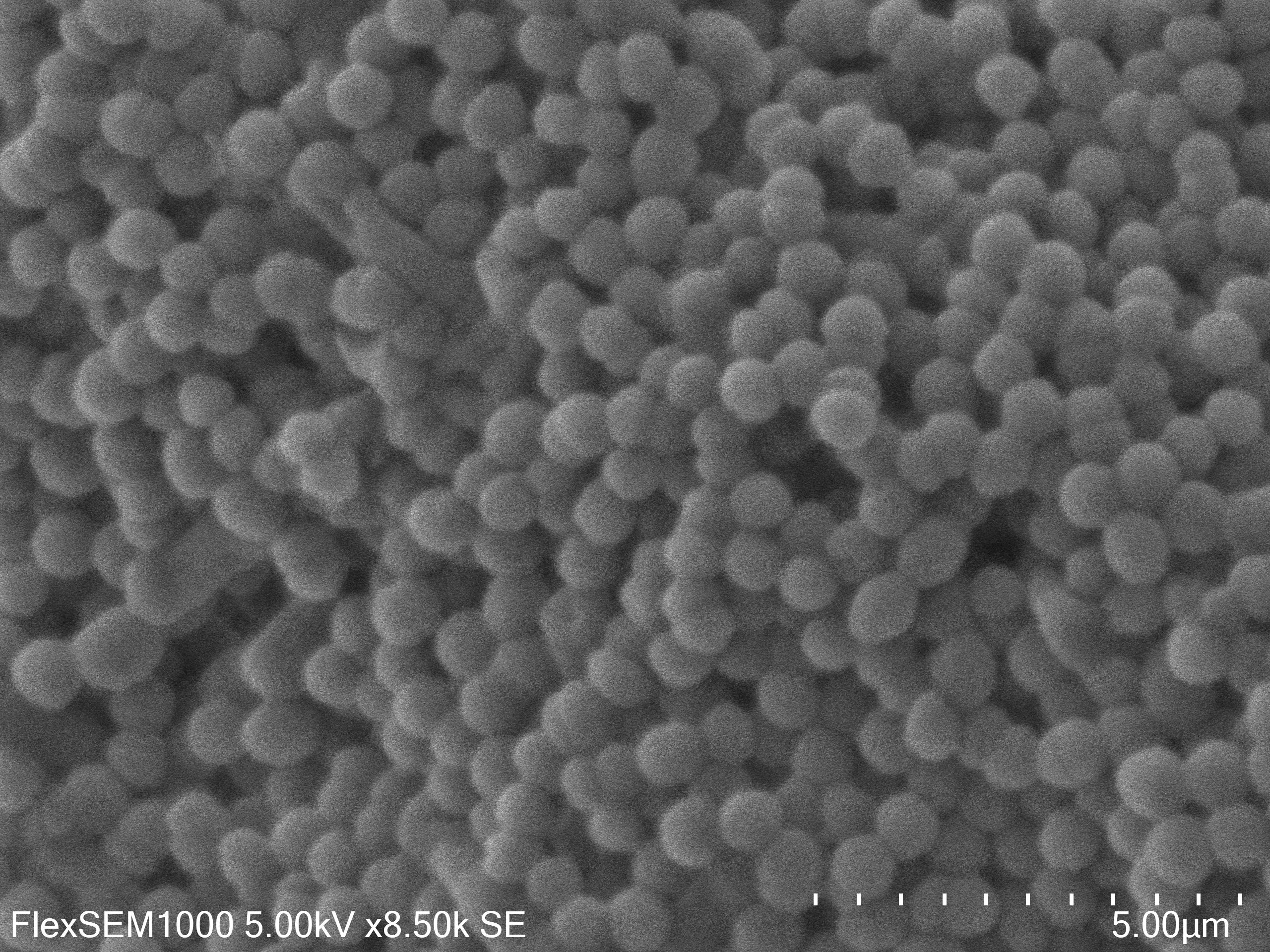

Supplement: Supplementary file 1 [file Data_Sheet_1.ZIP › Figure2-SEM/4×-1.jpg]

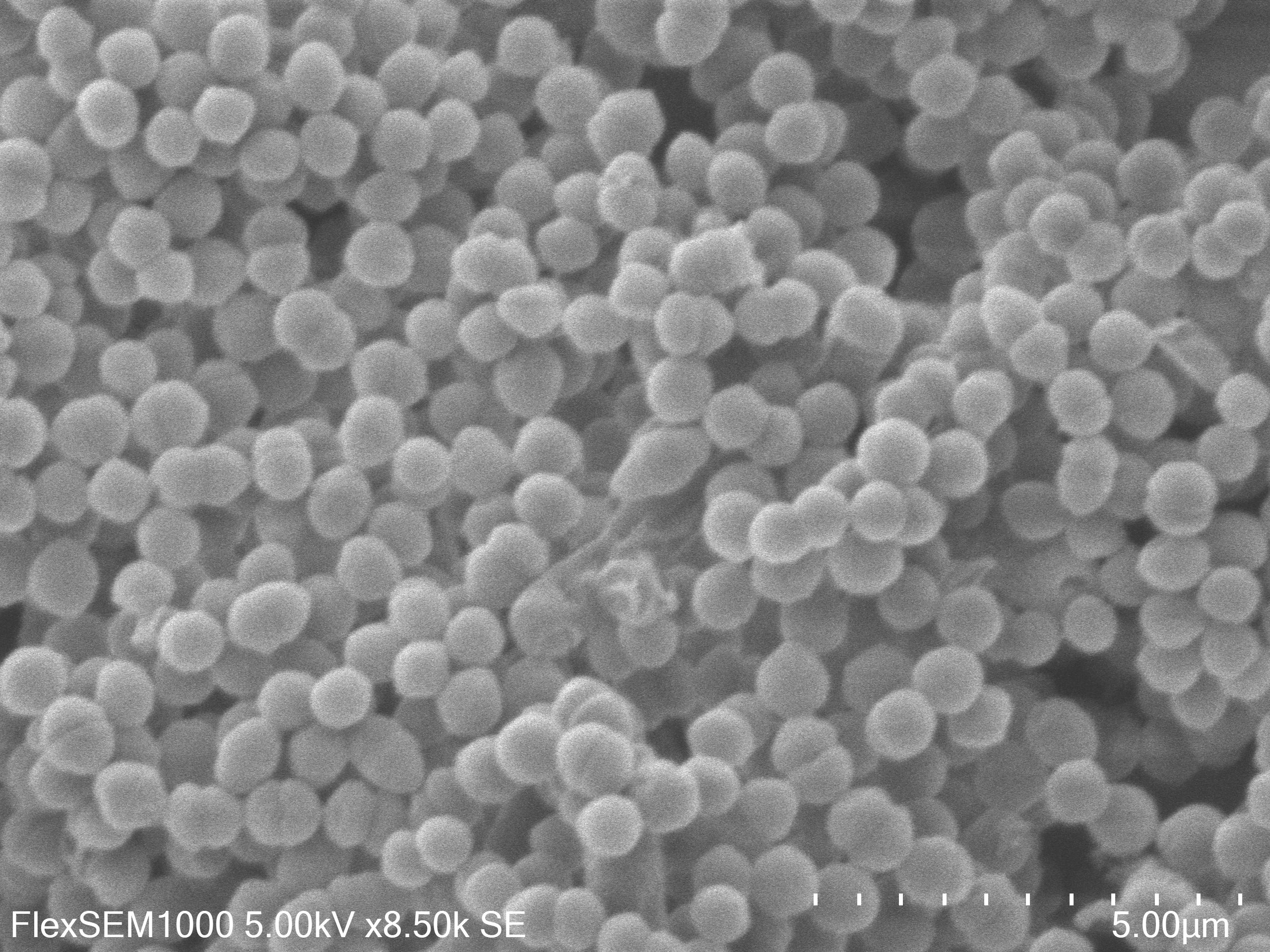

Supplement: Supplementary file 1 [file Data_Sheet_1.ZIP › Figure2-SEM/4×-2.jpg]

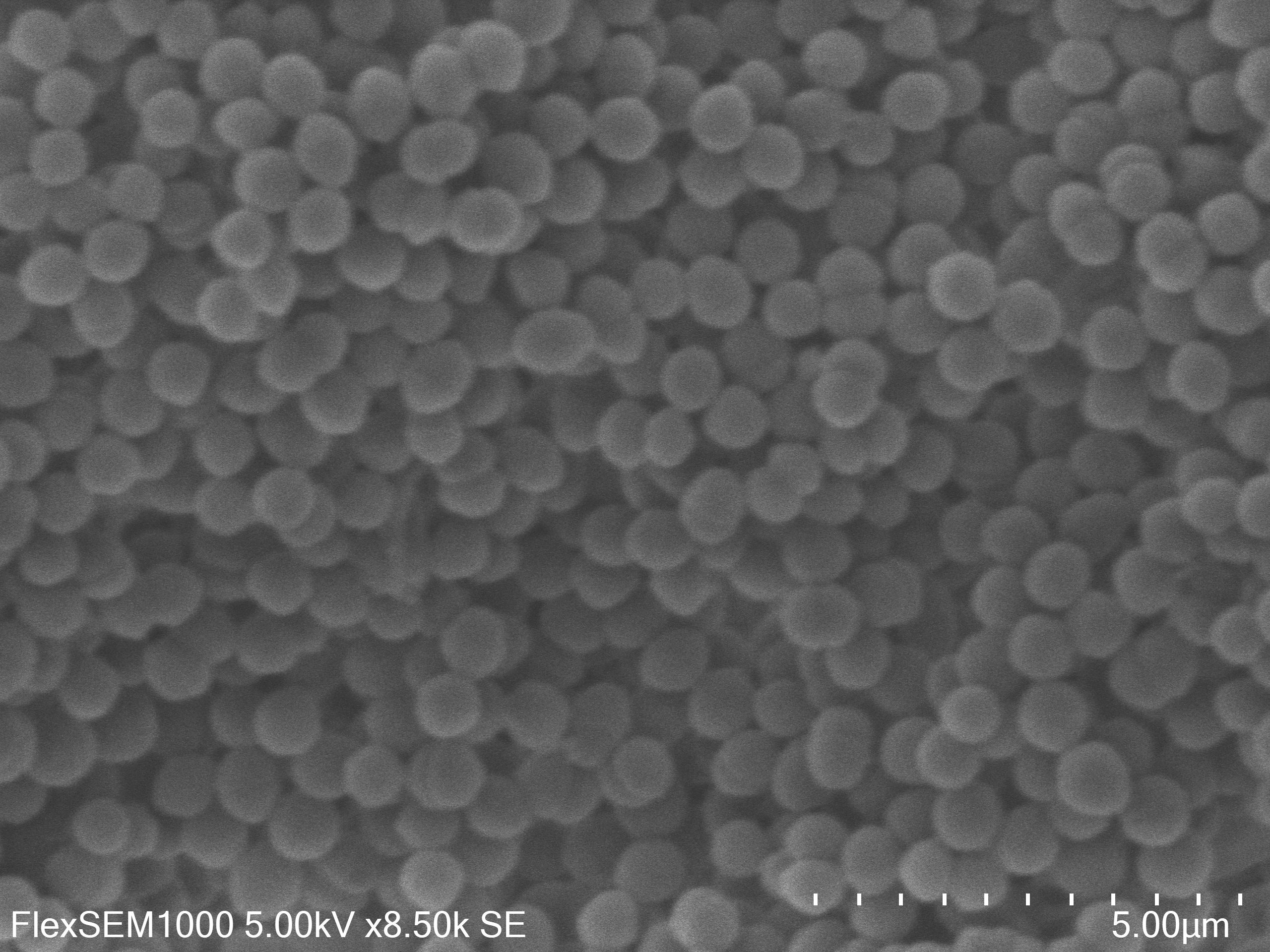

Supplement: Supplementary file 1 [file Data_Sheet_1.ZIP › Figure2-SEM/4×-3.jpg]

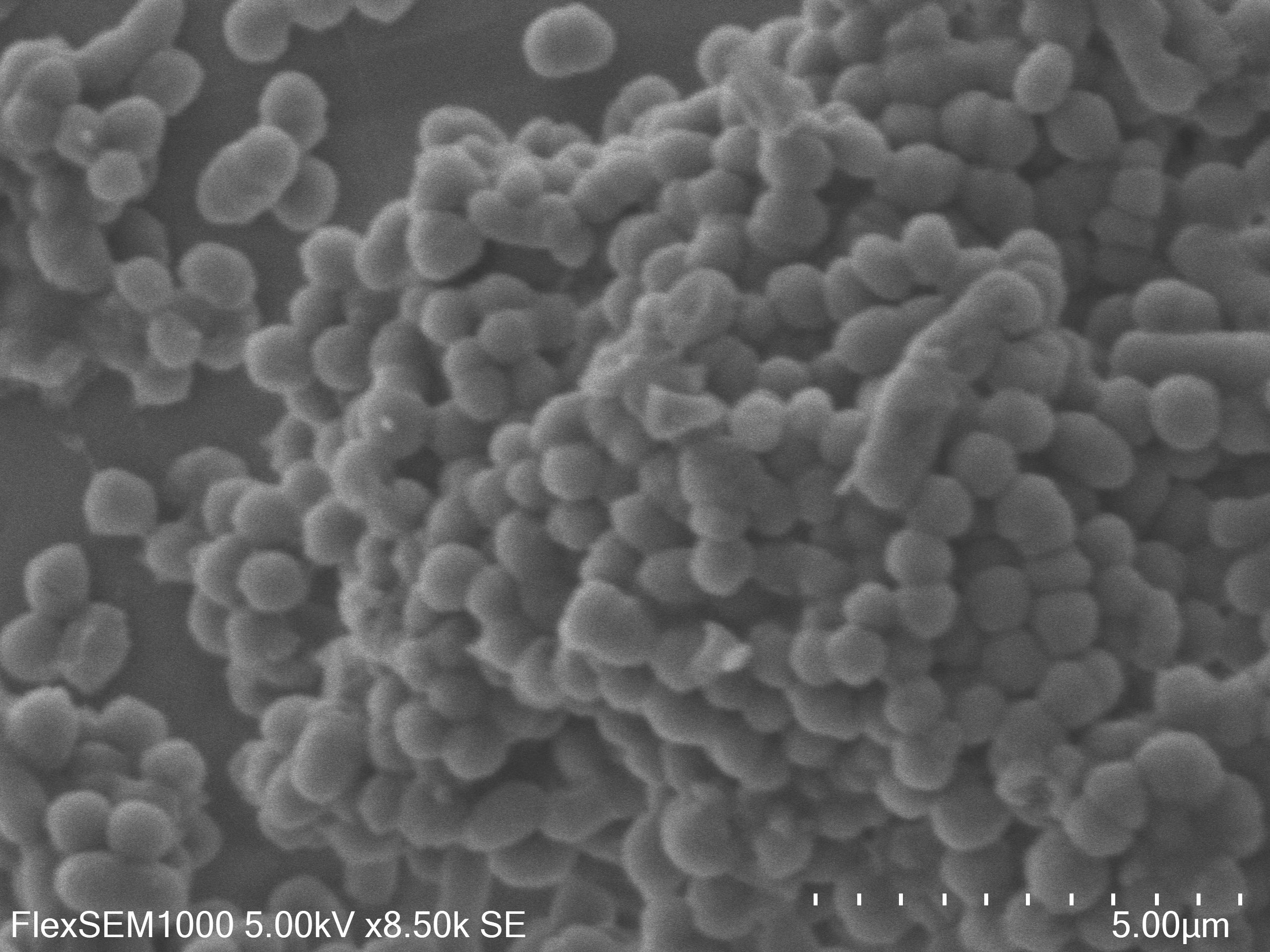

Supplement: Supplementary file 1 [file Data_Sheet_1.ZIP › Figure2-SEM/8×-1.jpg]

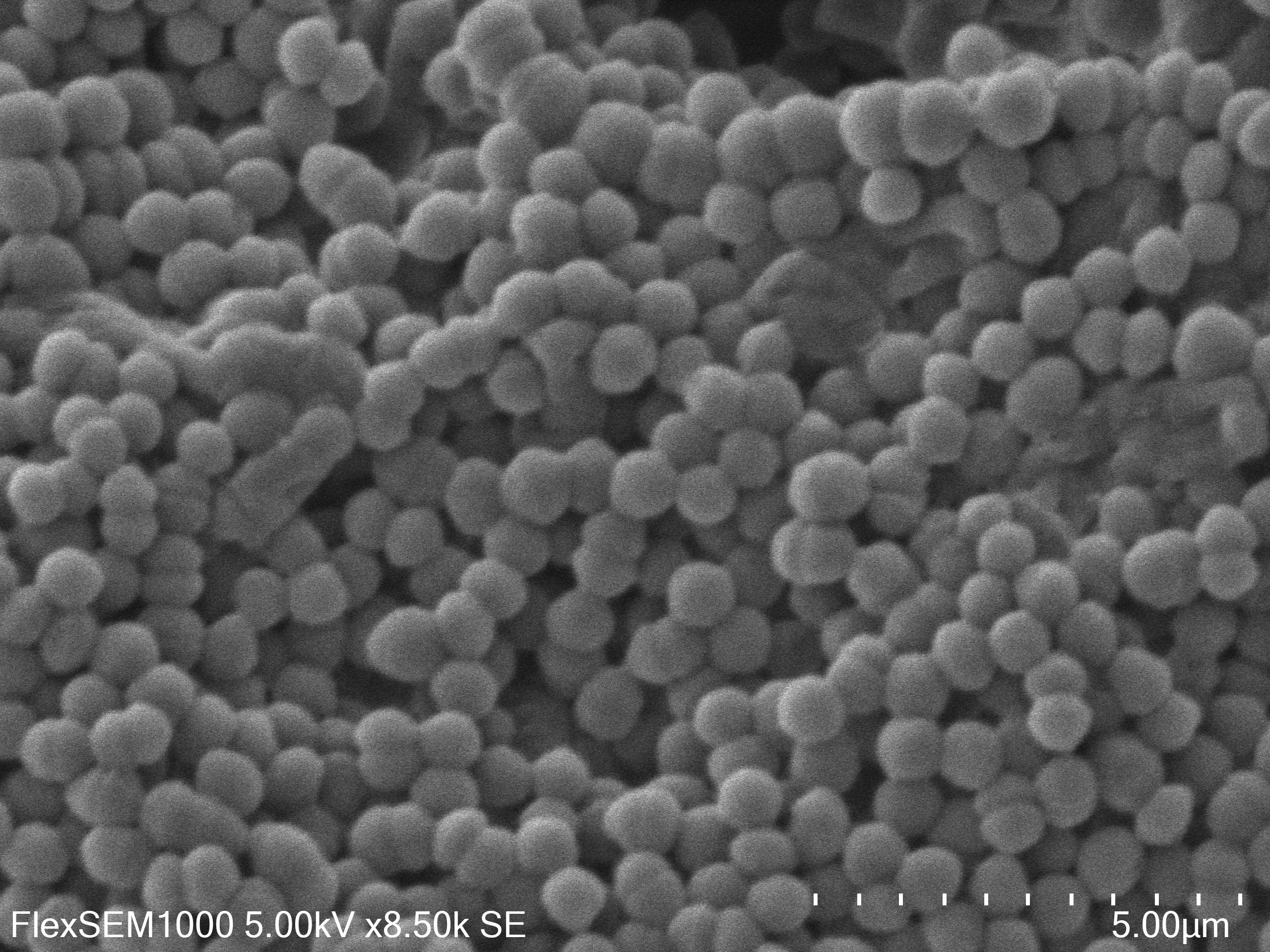

Supplement: Supplementary file 1 [file Data_Sheet_1.ZIP › Figure2-SEM/8×-2.jpg]

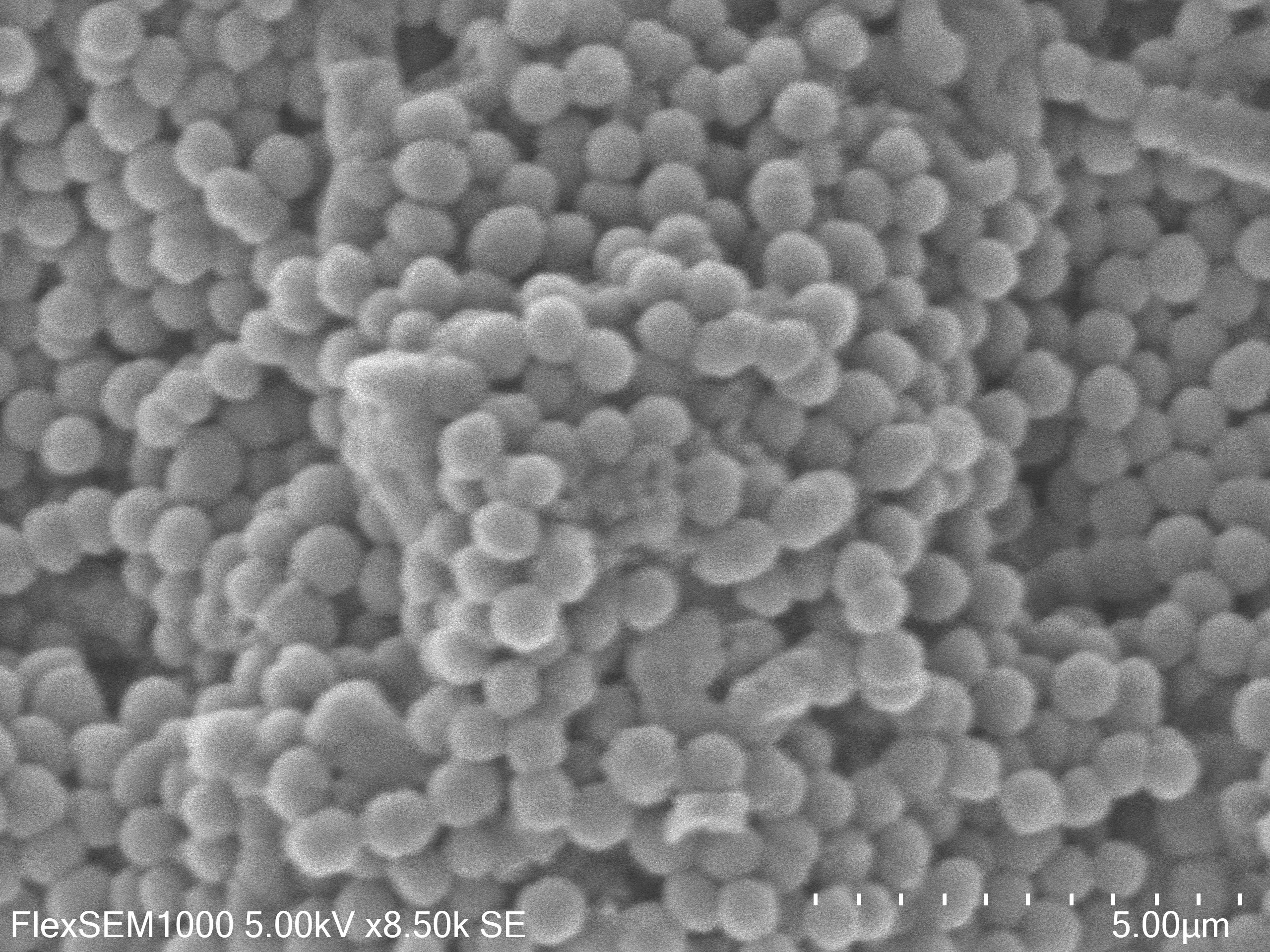

Supplement: Supplementary file 1 [file Data_Sheet_1.ZIP › Figure2-SEM/8×-3.jpg]

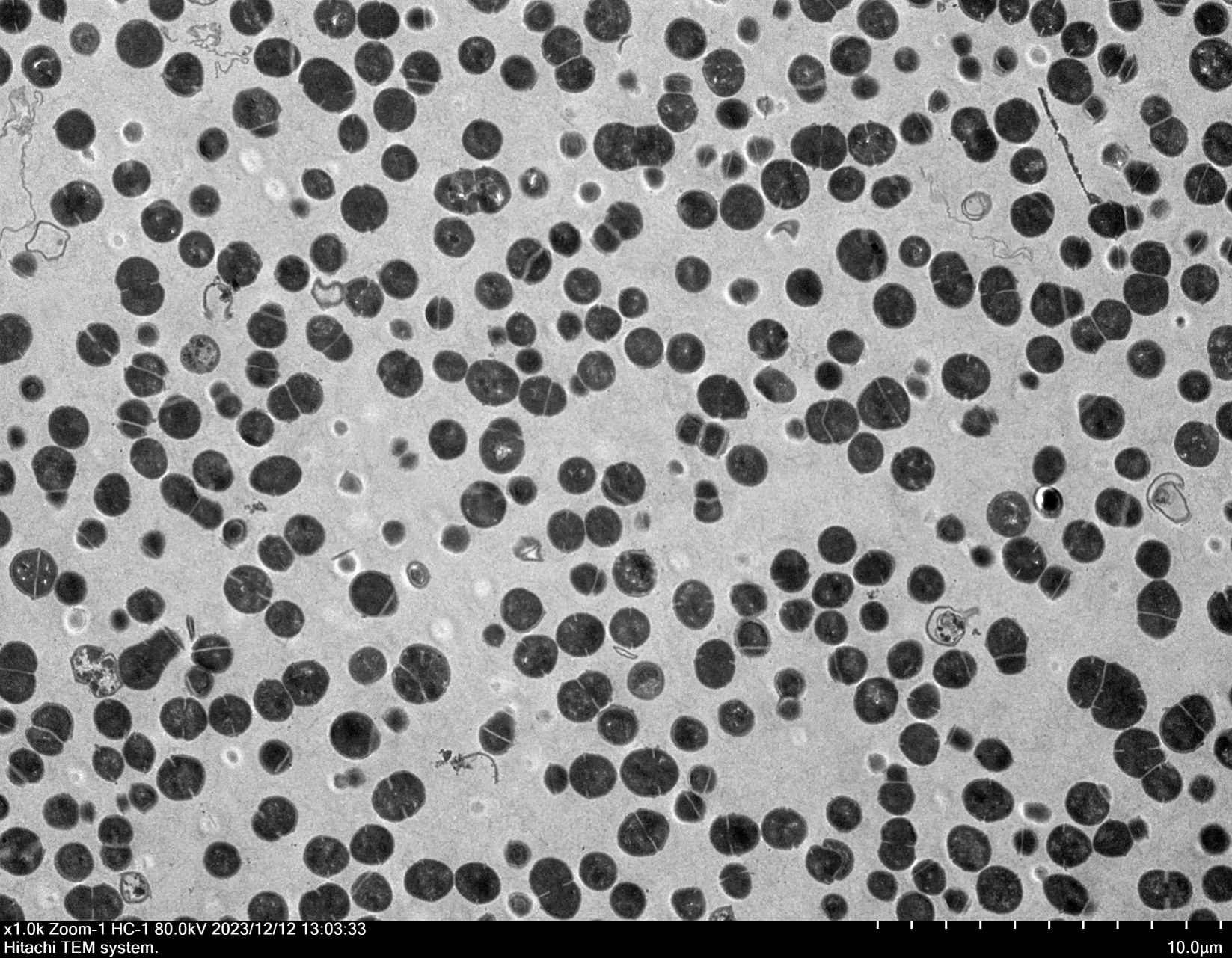

Supplement: Supplementary file 1 [file Data_Sheet_1.ZIP › Figure3-TEM/0×.jpg]

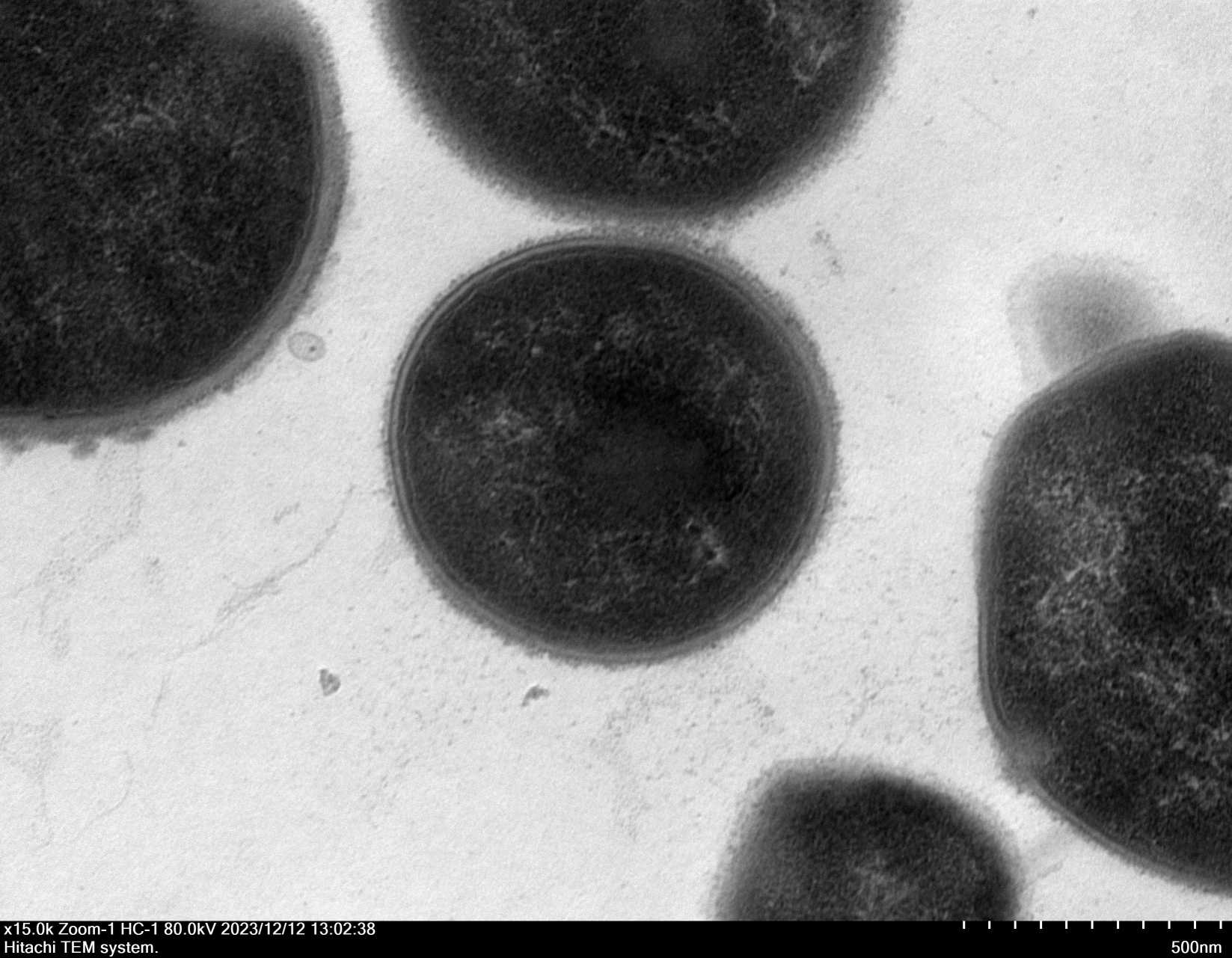

Supplement: Supplementary file 1 [file Data_Sheet_1.ZIP › Figure3-TEM/0×(1).jpg]

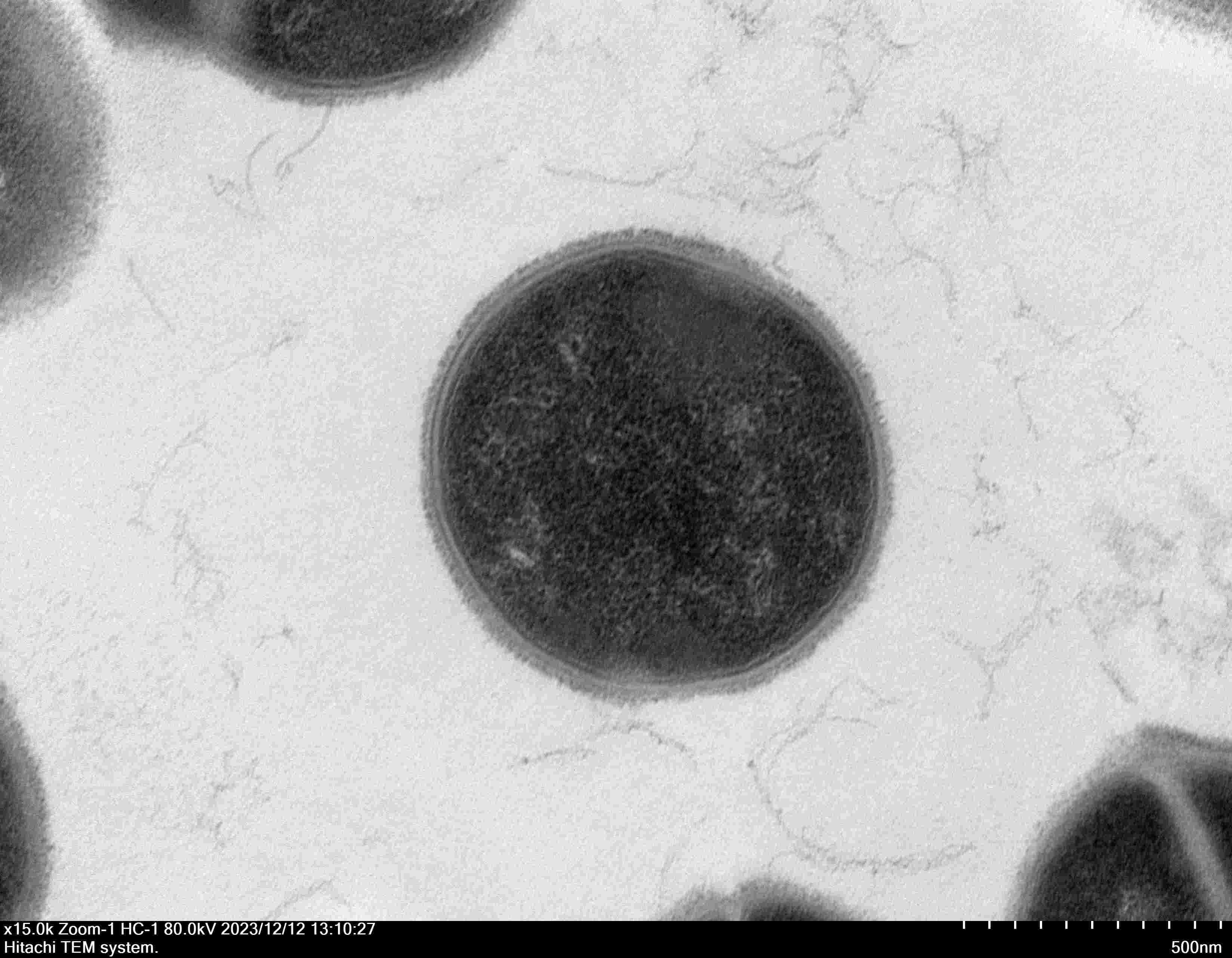

Supplement: Supplementary file 1 [file Data_Sheet_1.ZIP › Figure3-TEM/0×(2).jpg]

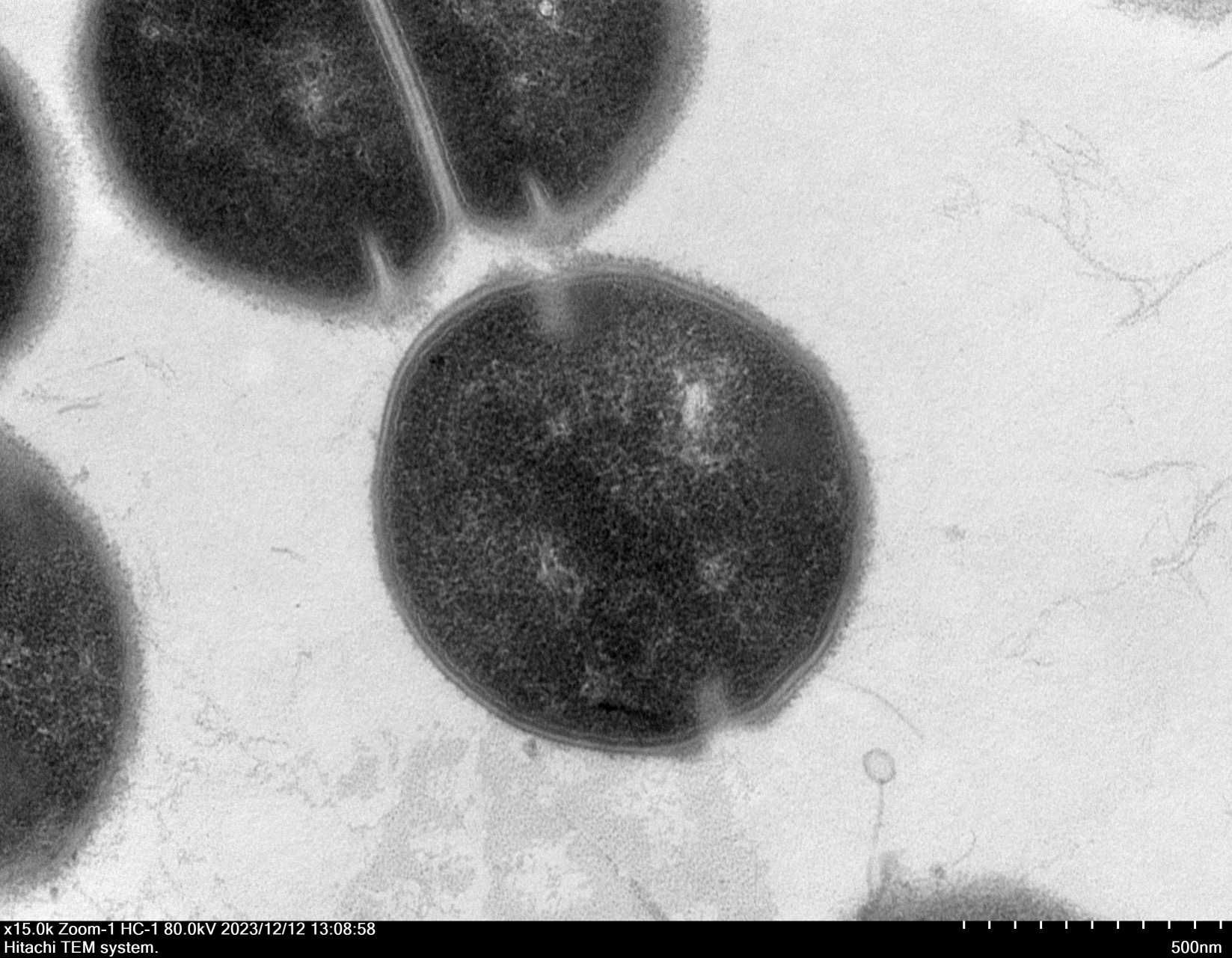

Supplement: Supplementary file 1 [file Data_Sheet_1.ZIP › Figure3-TEM/0×(3).jpg]

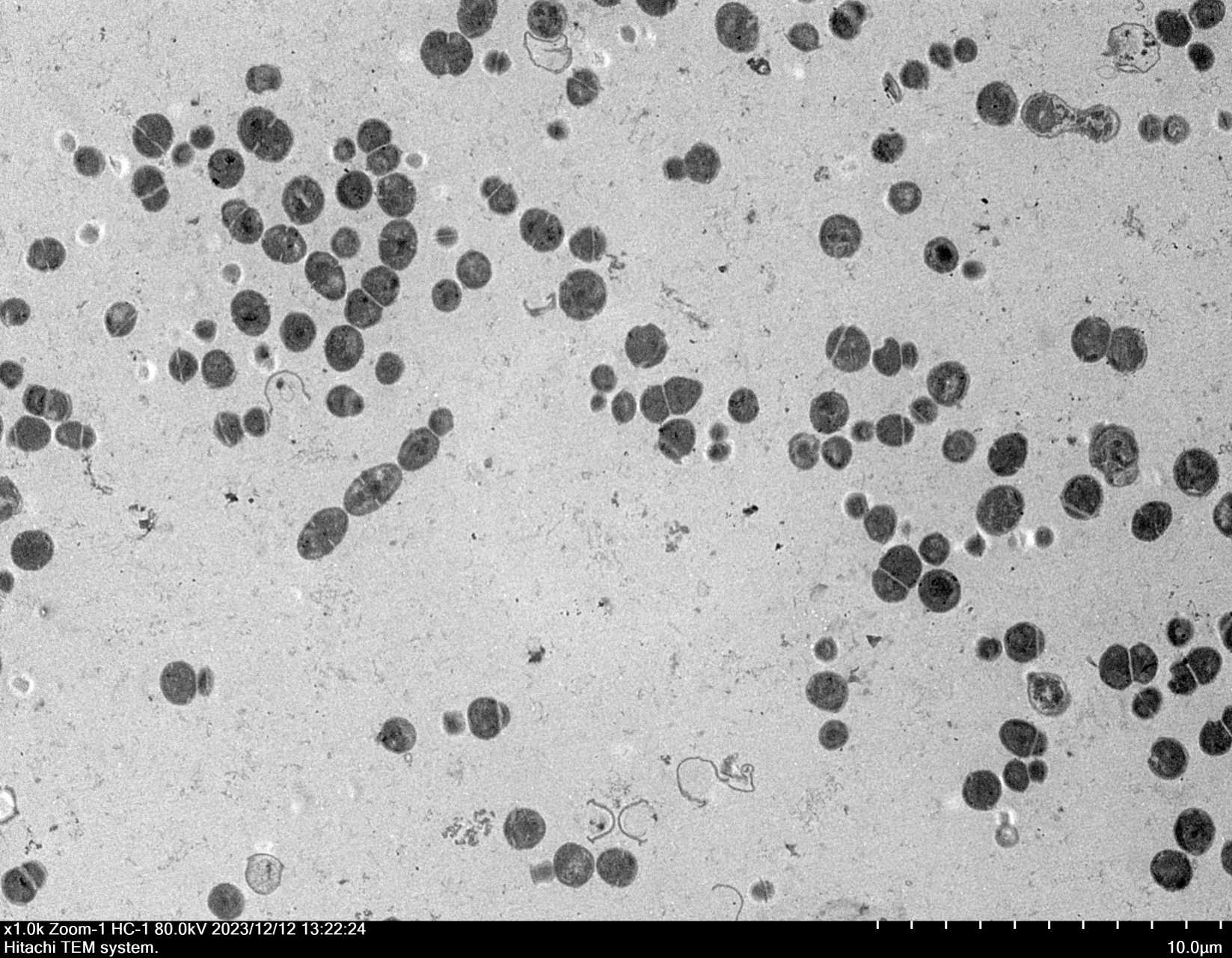

Supplement: Supplementary file 1 [file Data_Sheet_1.ZIP › Figure3-TEM/2×.jpg]

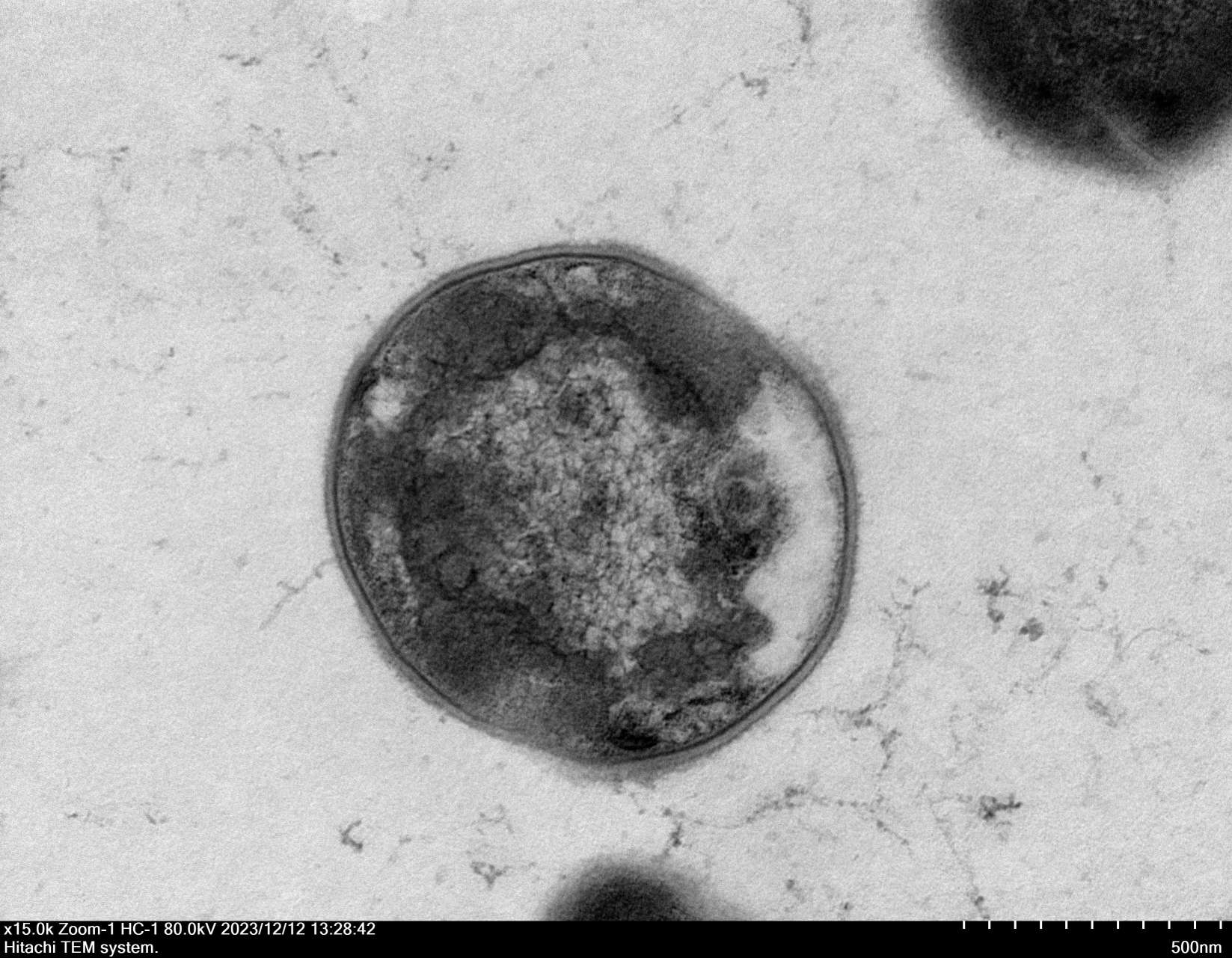

Supplement: Supplementary file 1 [file Data_Sheet_1.ZIP › Figure3-TEM/2×(1).jpg]

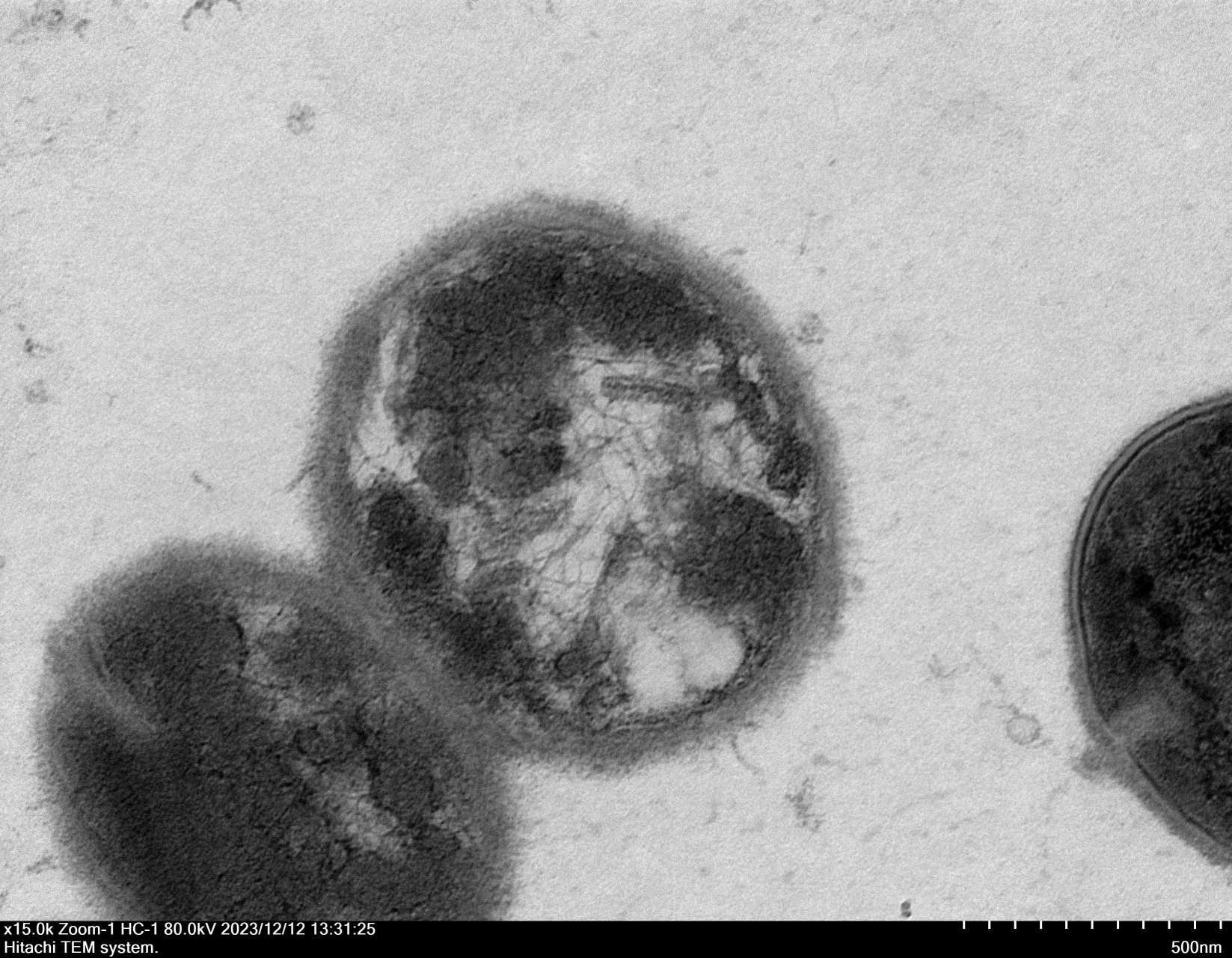

Supplement: Supplementary file 1 [file Data_Sheet_1.ZIP › Figure3-TEM/2×(2).jpg]

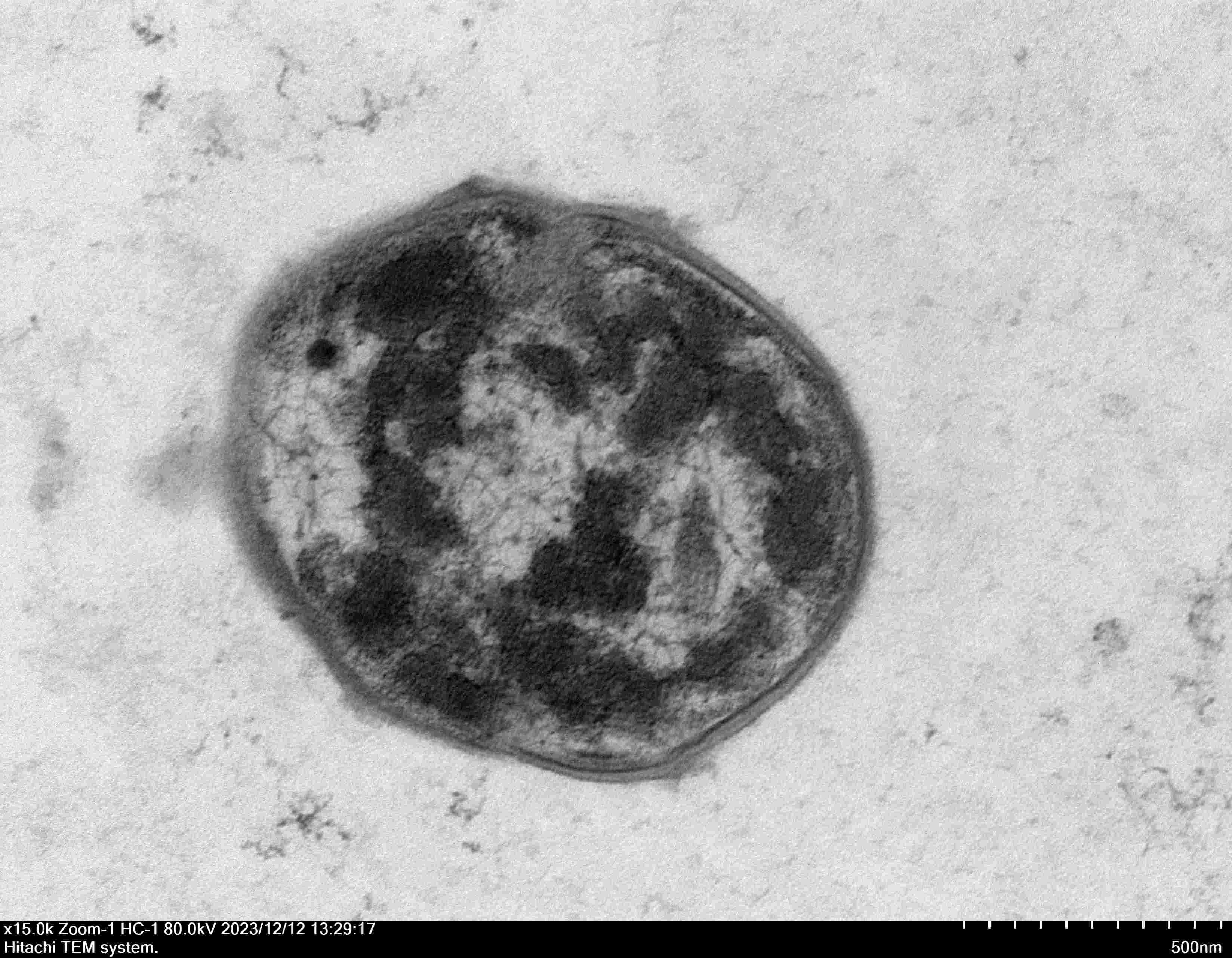

Supplement: Supplementary file 1 [file Data_Sheet_1.ZIP › Figure3-TEM/2×(3).jpg]

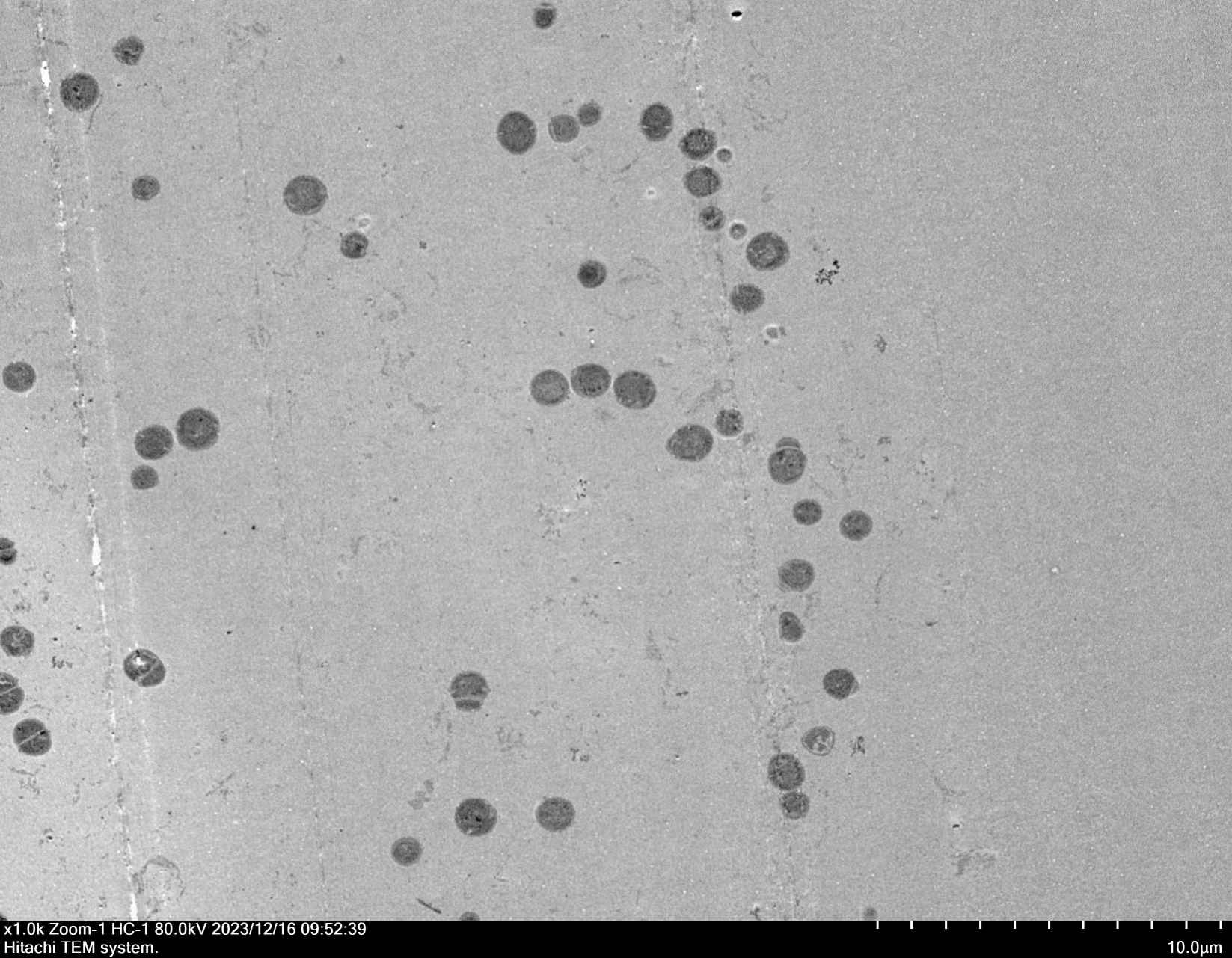

Supplement: Supplementary file 1 [file Data_Sheet_1.ZIP › Figure3-TEM/4×.jpg]

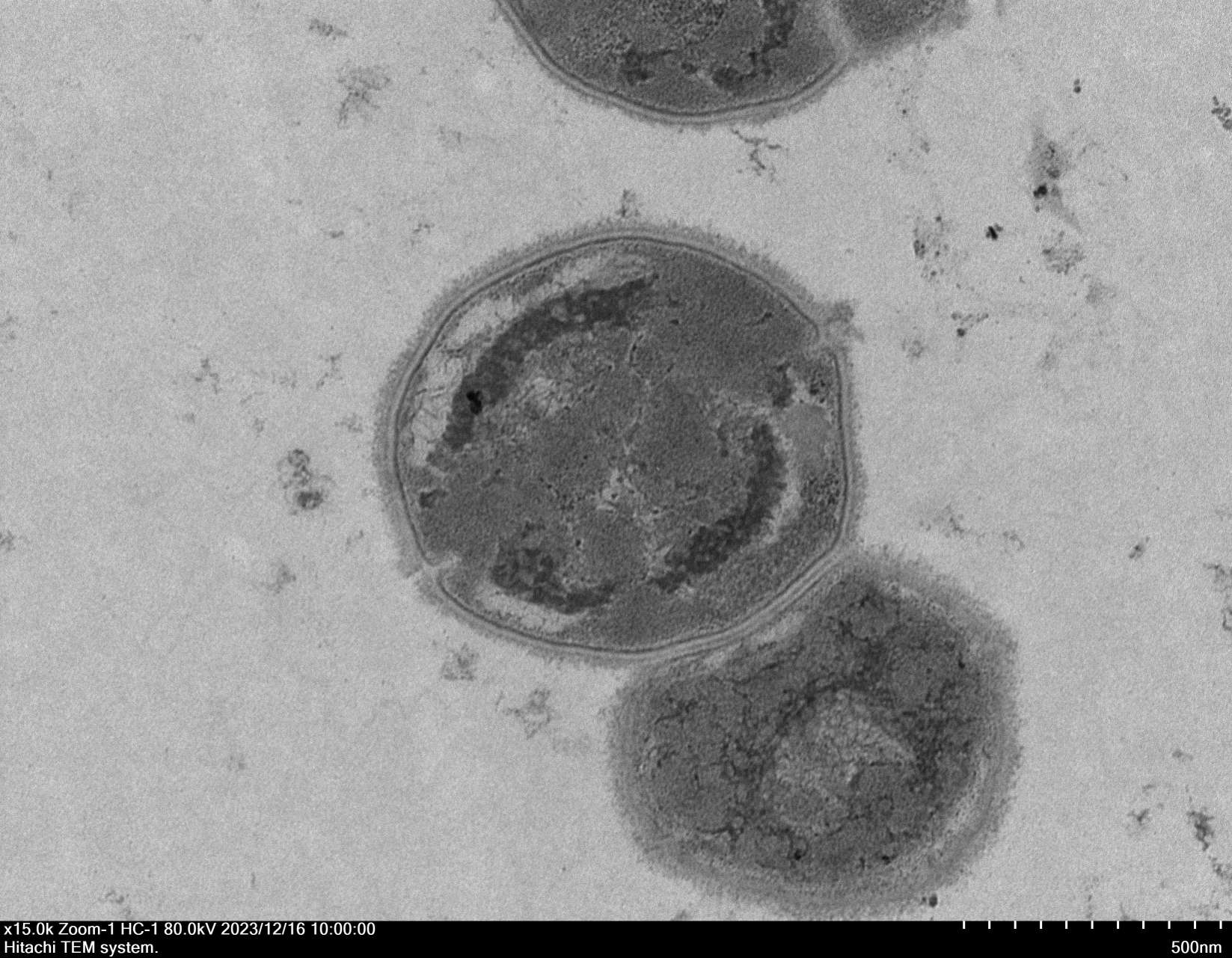

Supplement: Supplementary file 1 [file Data_Sheet_1.ZIP › Figure3-TEM/4×(1).jpg]

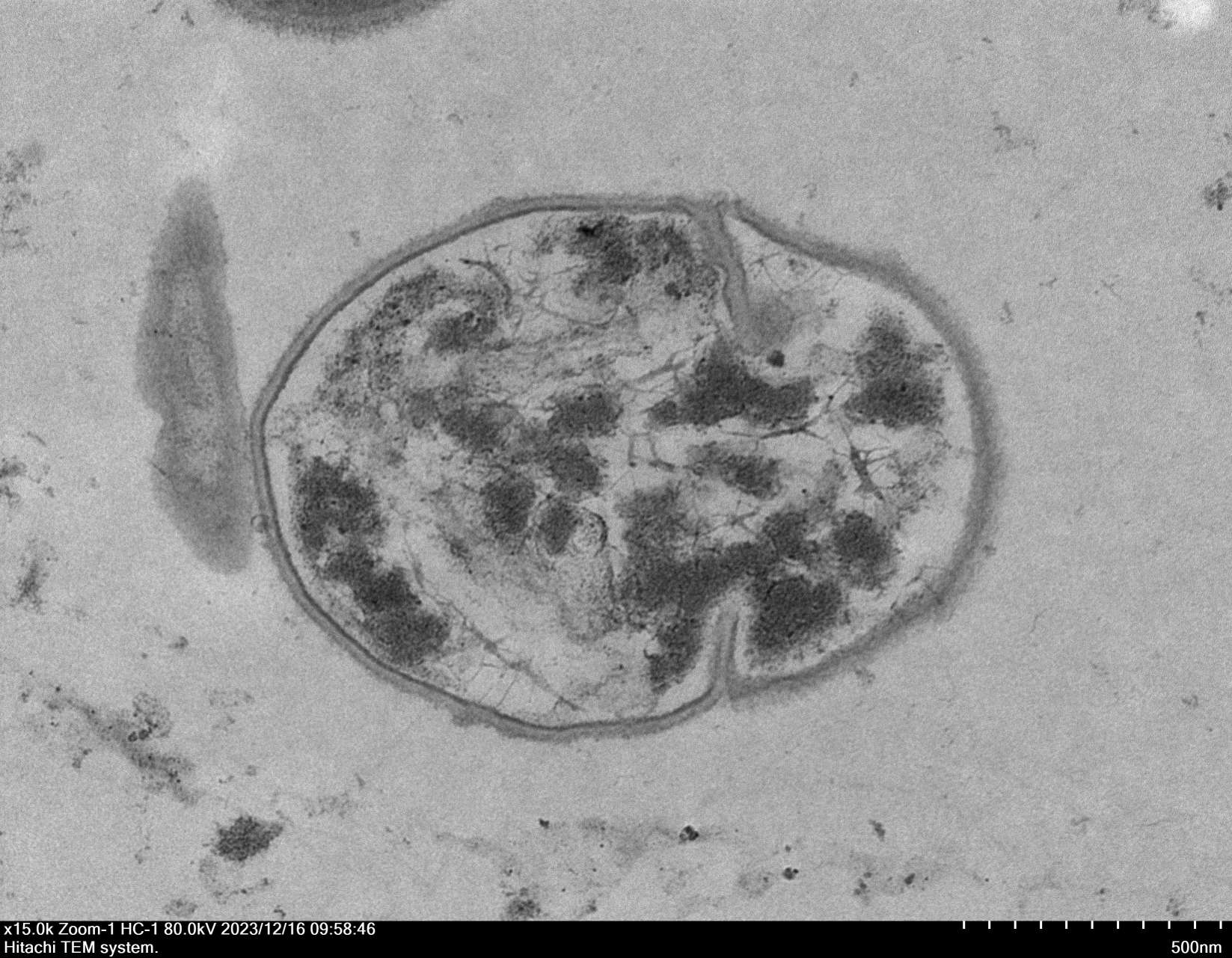

Supplement: Supplementary file 1 [file Data_Sheet_1.ZIP › Figure3-TEM/4×(2).jpg]

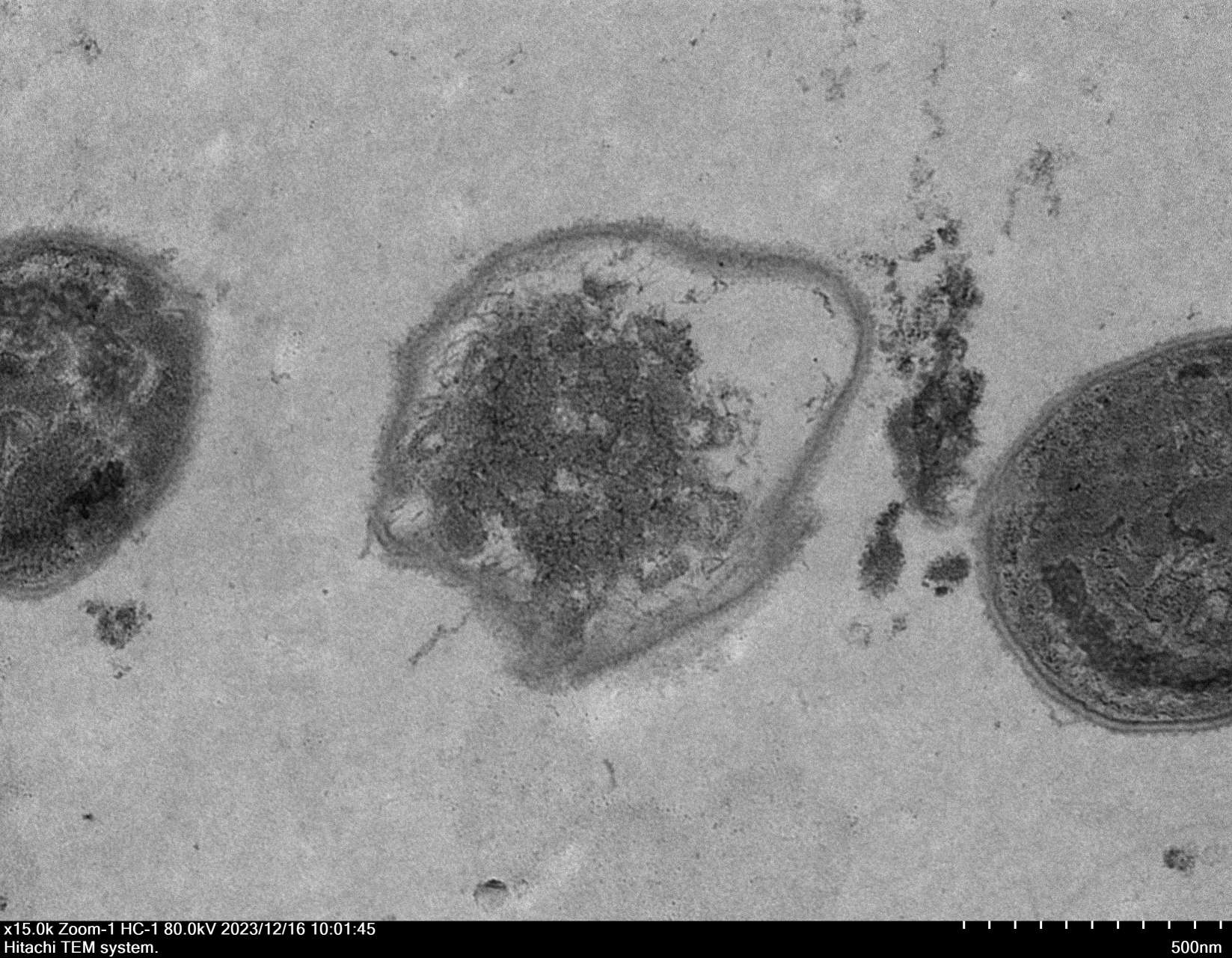

Supplement: Supplementary file 1 [file Data_Sheet_1.ZIP › Figure3-TEM/4×(3).jpg]

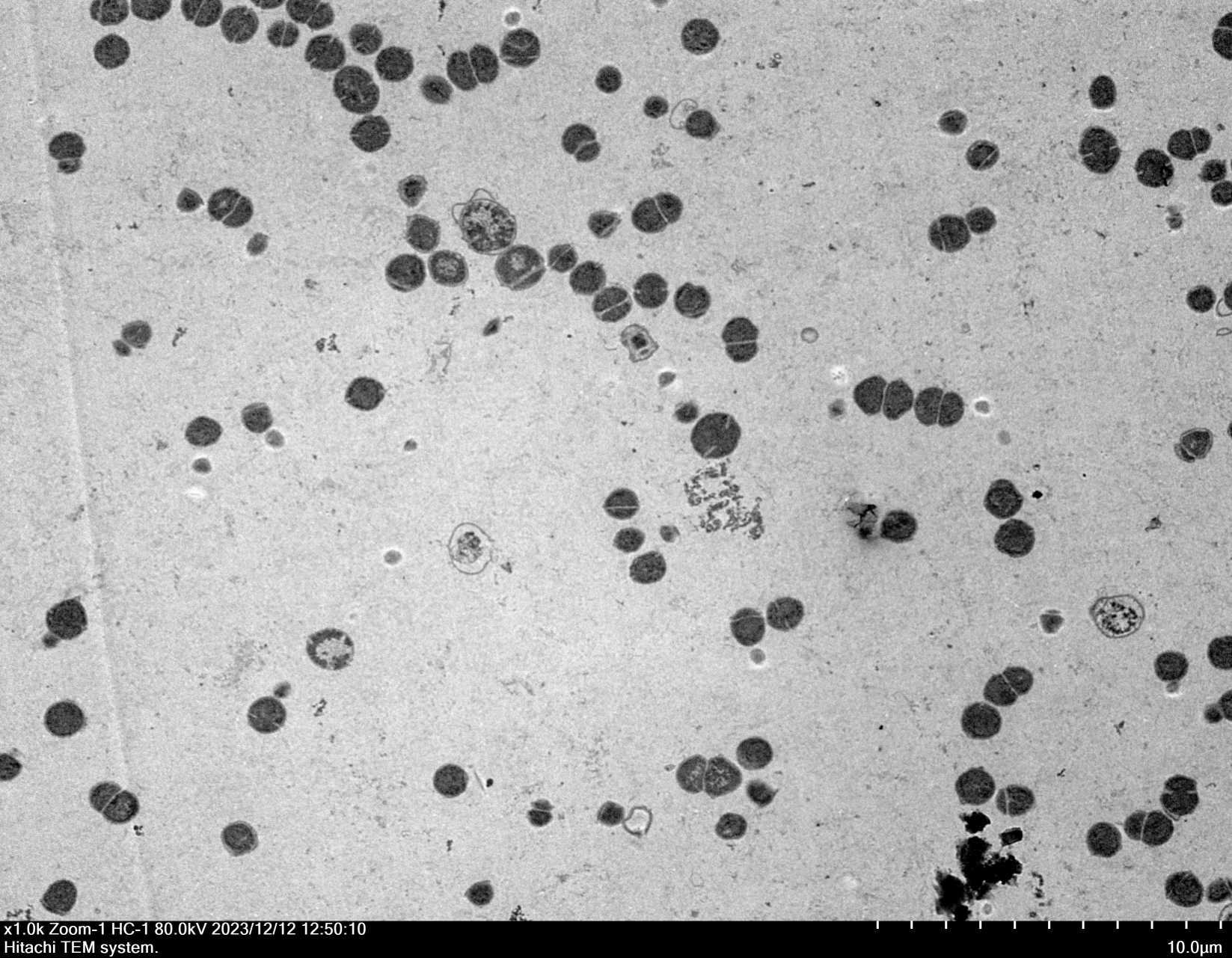

Supplement: Supplementary file 1 [file Data_Sheet_1.ZIP › Figure3-TEM/8×.jpg]

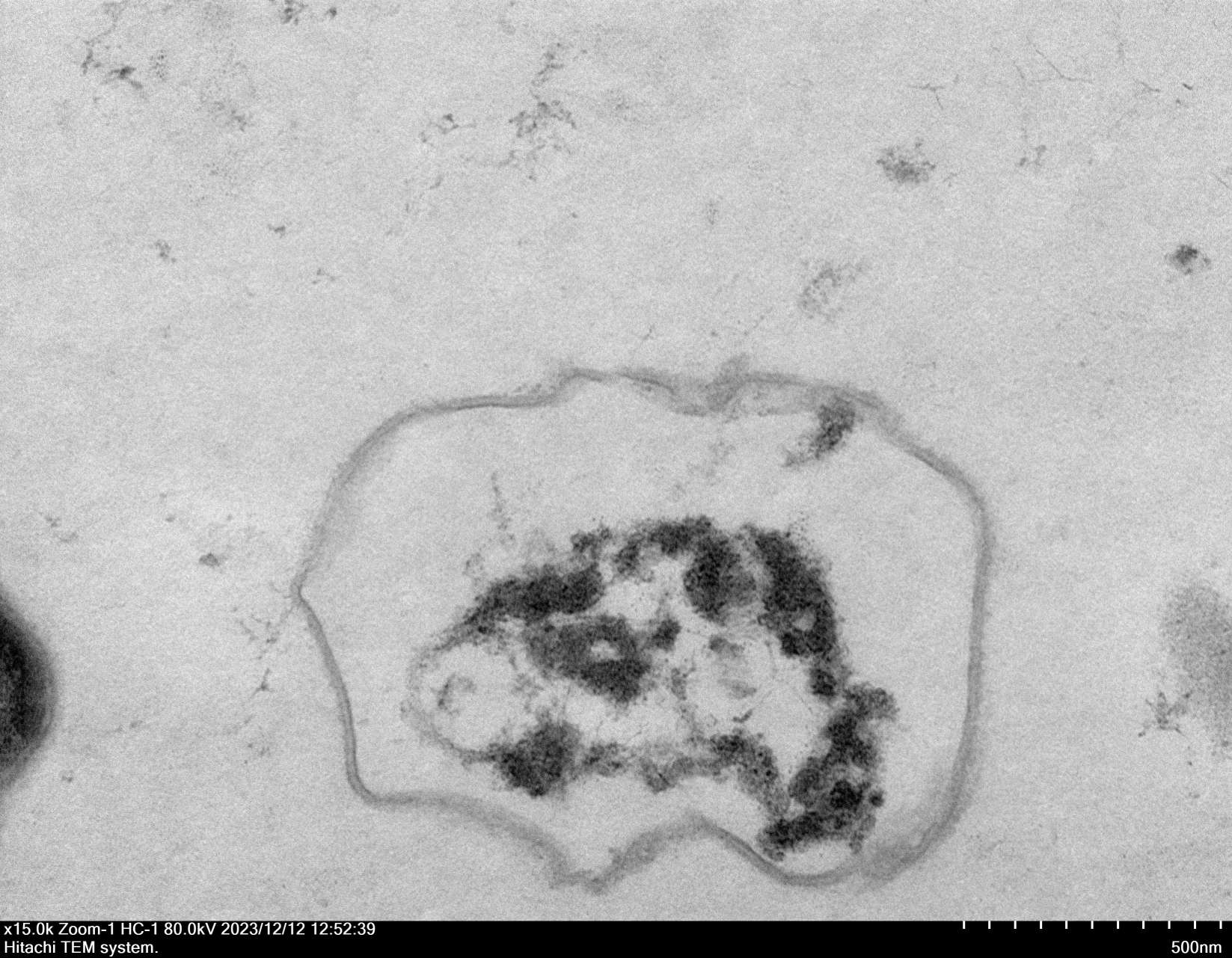

Supplement: Supplementary file 1 [file Data_Sheet_1.ZIP › Figure3-TEM/8×(1).jpg]

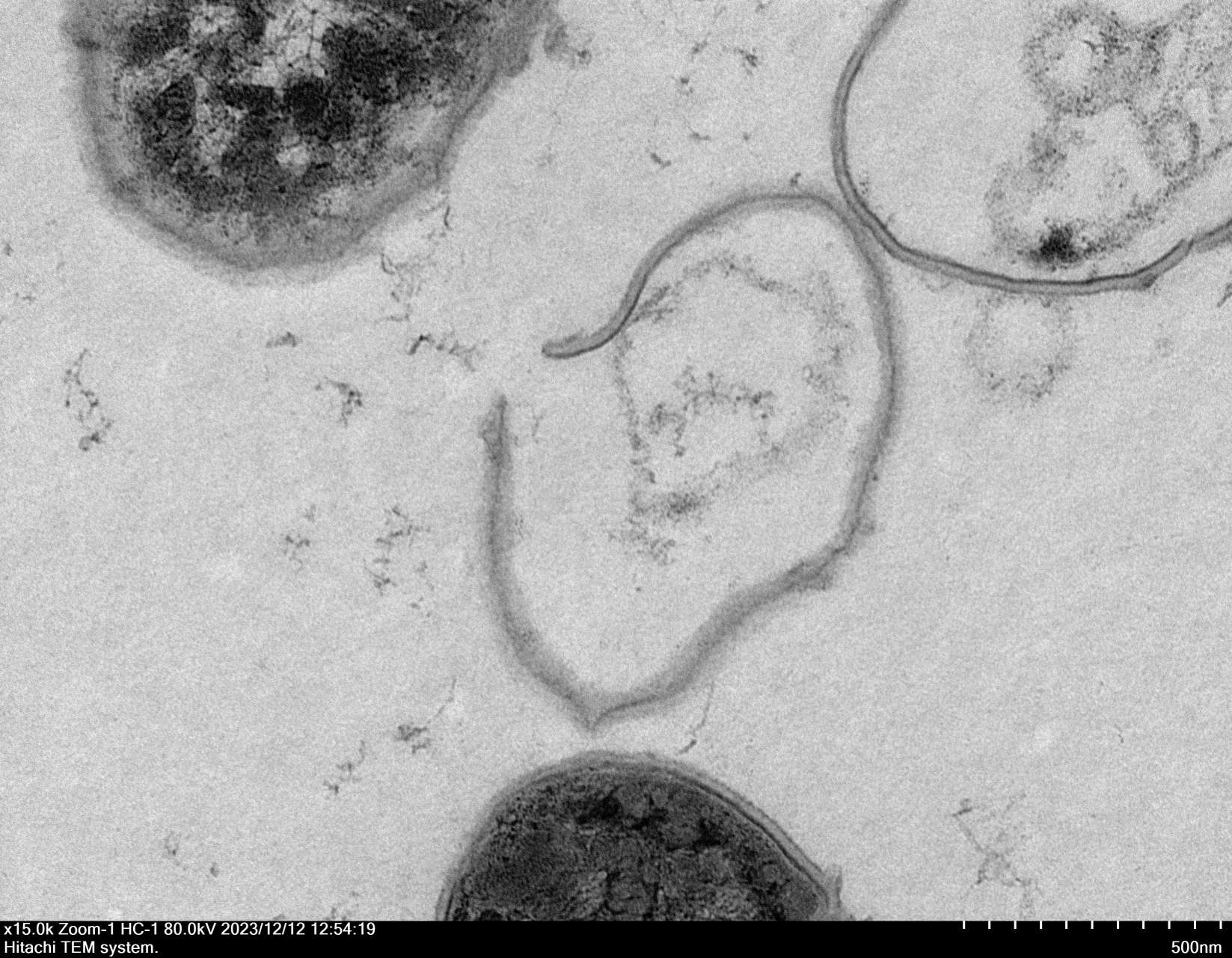

Supplement: Supplementary file 1 [file Data_Sheet_1.ZIP › Figure3-TEM/8×(2).jpg]

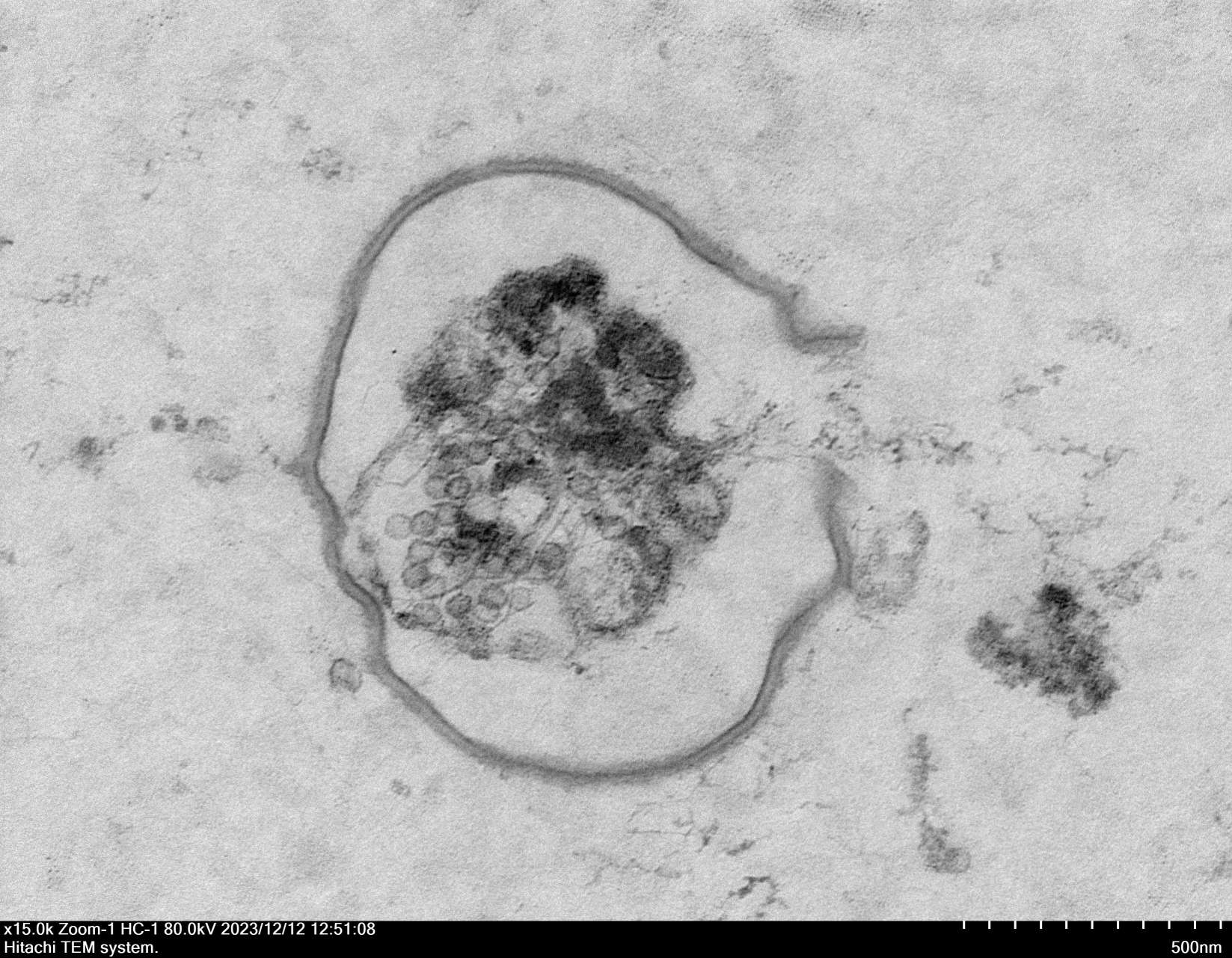

Supplement: Supplementary file 1 [file Data_Sheet_1.ZIP › Figure3-TEM/8×(3).jpg]

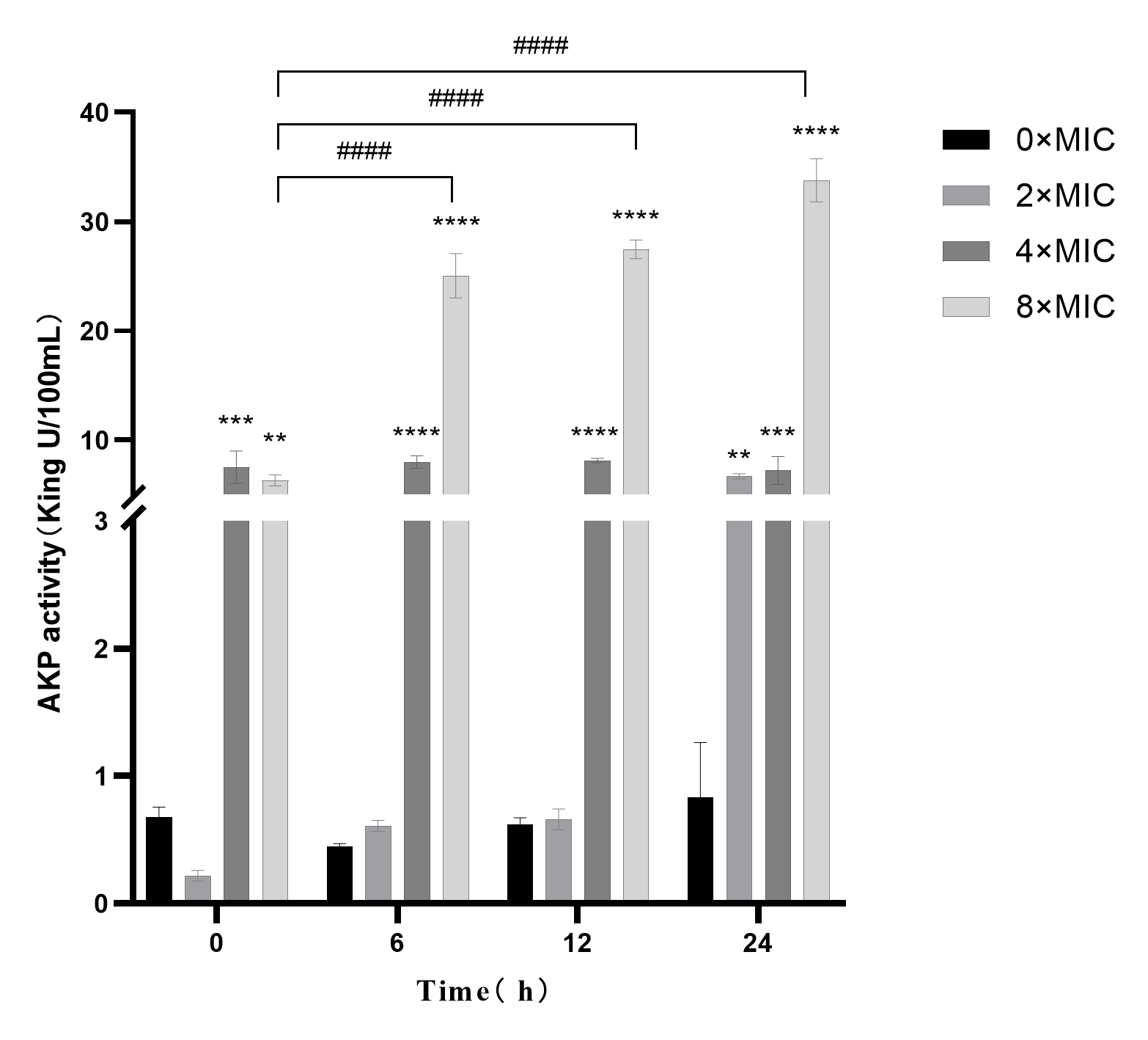

Supplement: Supplementary file 1 [file Data_Sheet_1.ZIP › Figure4-AKP Activity/AKP.tif]

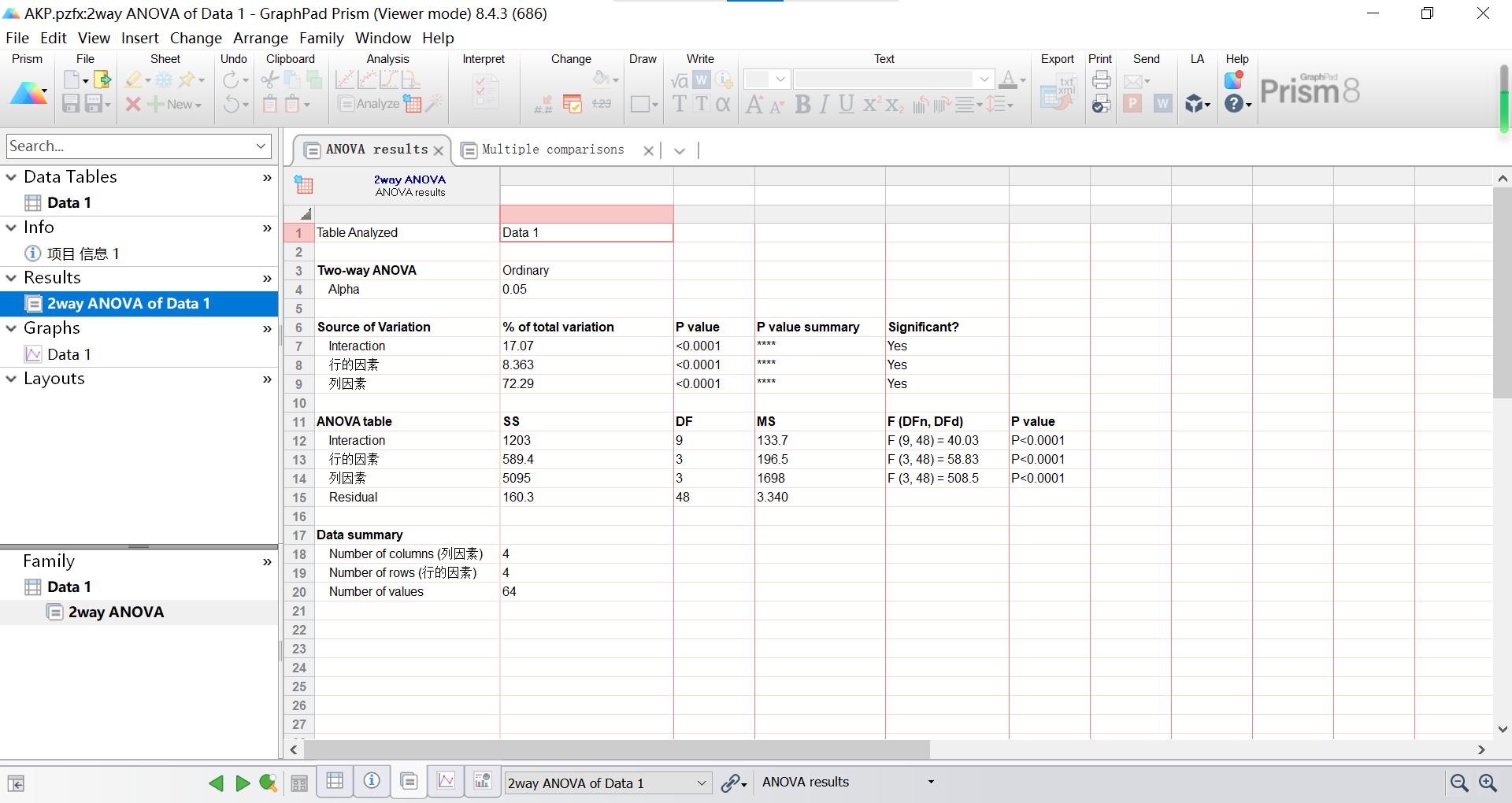

Supplement: Supplementary file 1 [file Data_Sheet_1.ZIP › Figure4-AKP Activity/screenshots of GraphPad Prism 8.0/2way ANOVA of AKP.jpg]

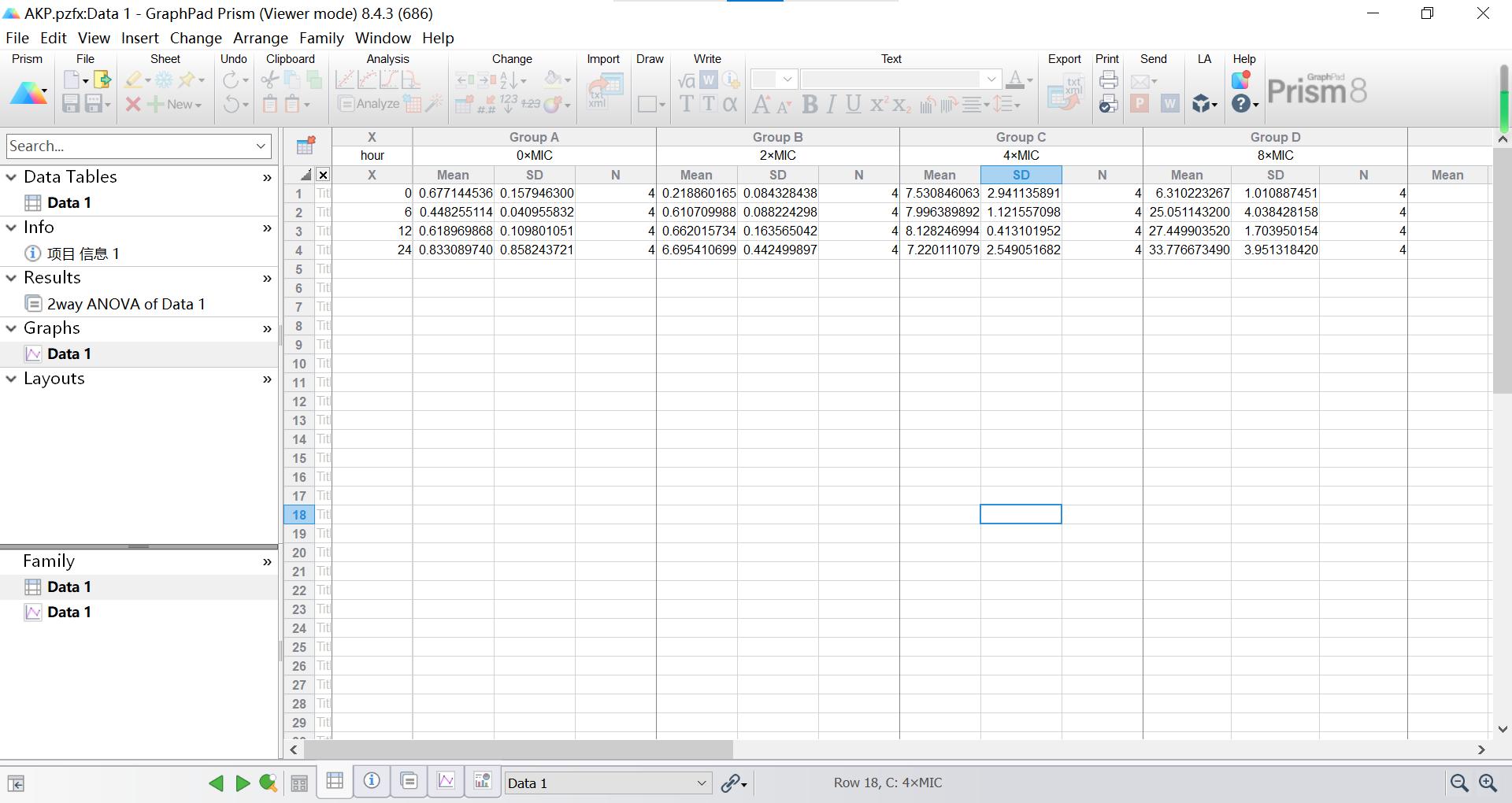

Supplement: Supplementary file 1 [file Data_Sheet_1.ZIP › Figure4-AKP Activity/screenshots of GraphPad Prism 8.0/AKP Data.jpg]

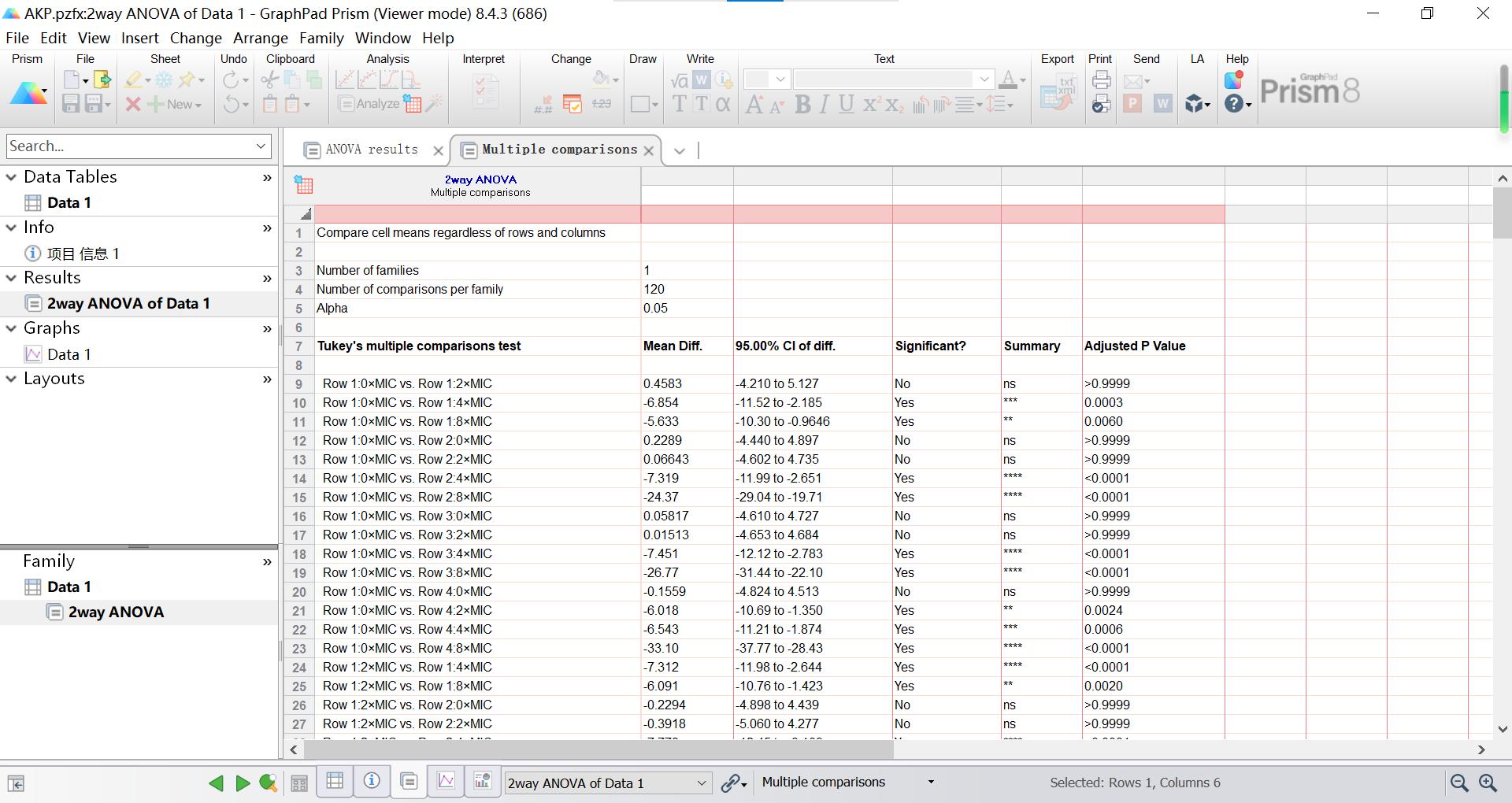

Supplement: Supplementary file 1 [file Data_Sheet_1.ZIP › Figure4-AKP Activity/screenshots of GraphPad Prism 8.0/Multiple comparisons1-1.jpg]

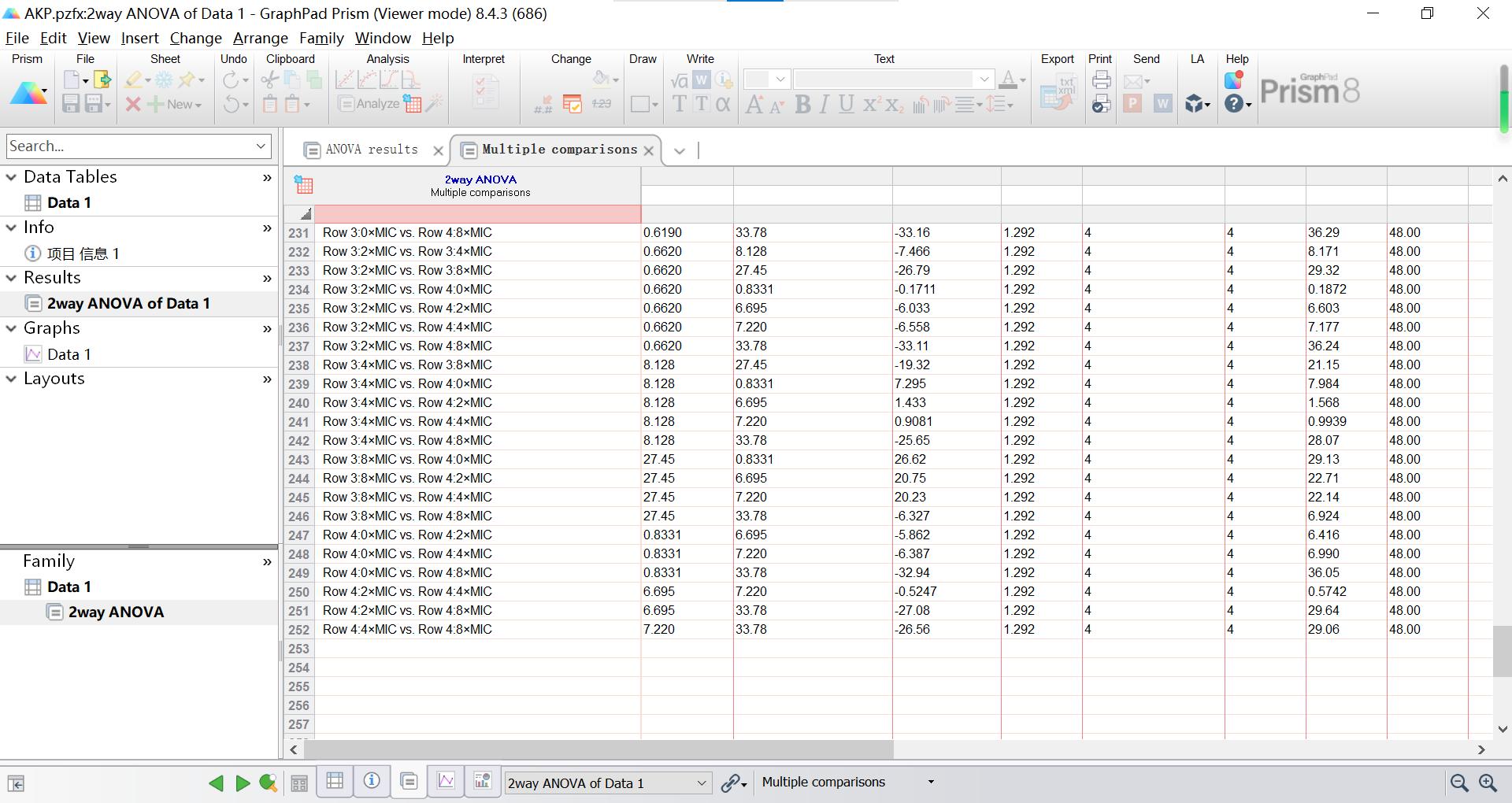

Supplement: Supplementary file 1 [file Data_Sheet_1.ZIP › Figure4-AKP Activity/screenshots of GraphPad Prism 8.0/Multiple comparisons1-10.jpg]

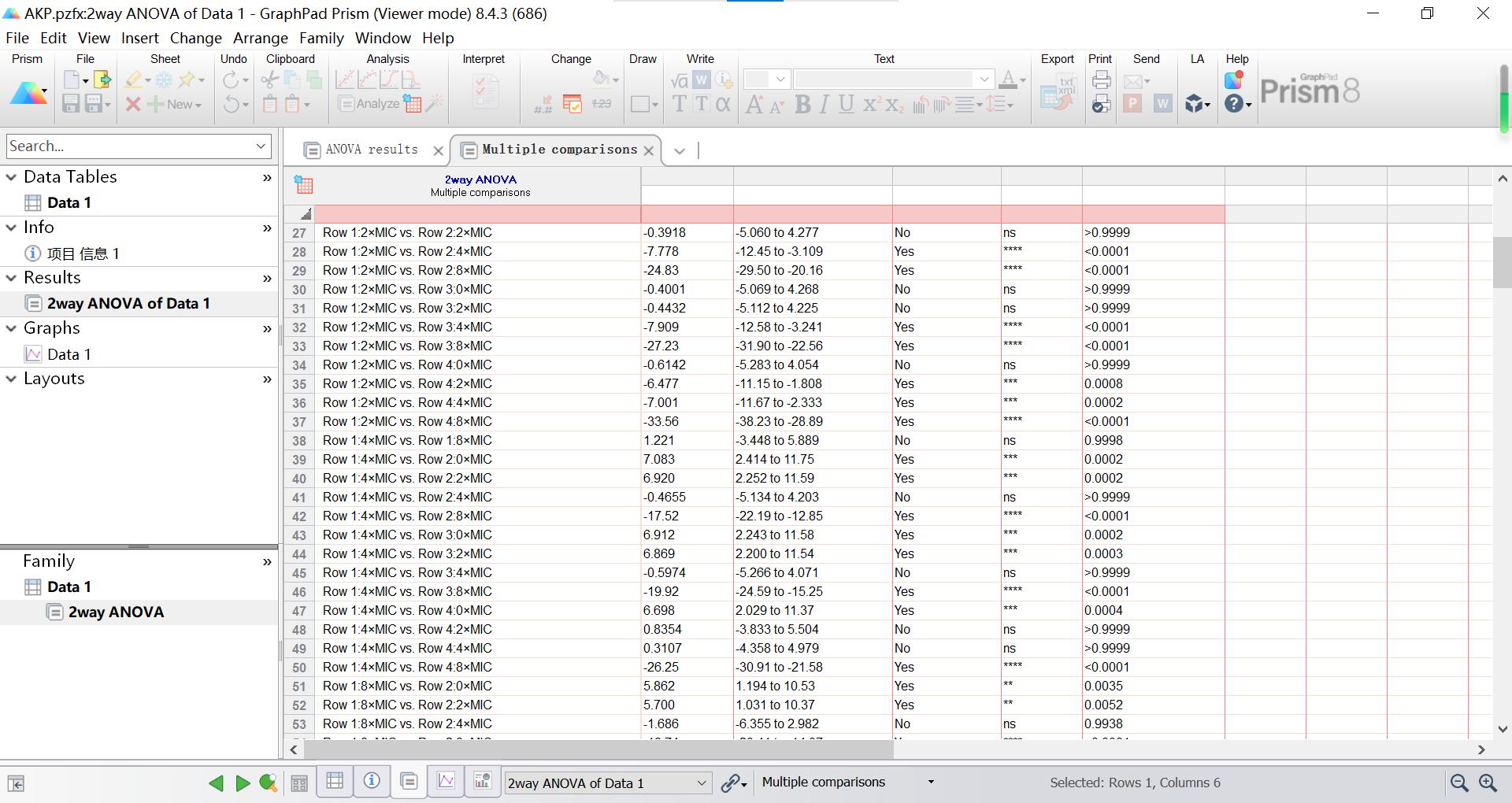

Supplement: Supplementary file 1 [file Data_Sheet_1.ZIP › Figure4-AKP Activity/screenshots of GraphPad Prism 8.0/Multiple comparisons1-2.jpg]

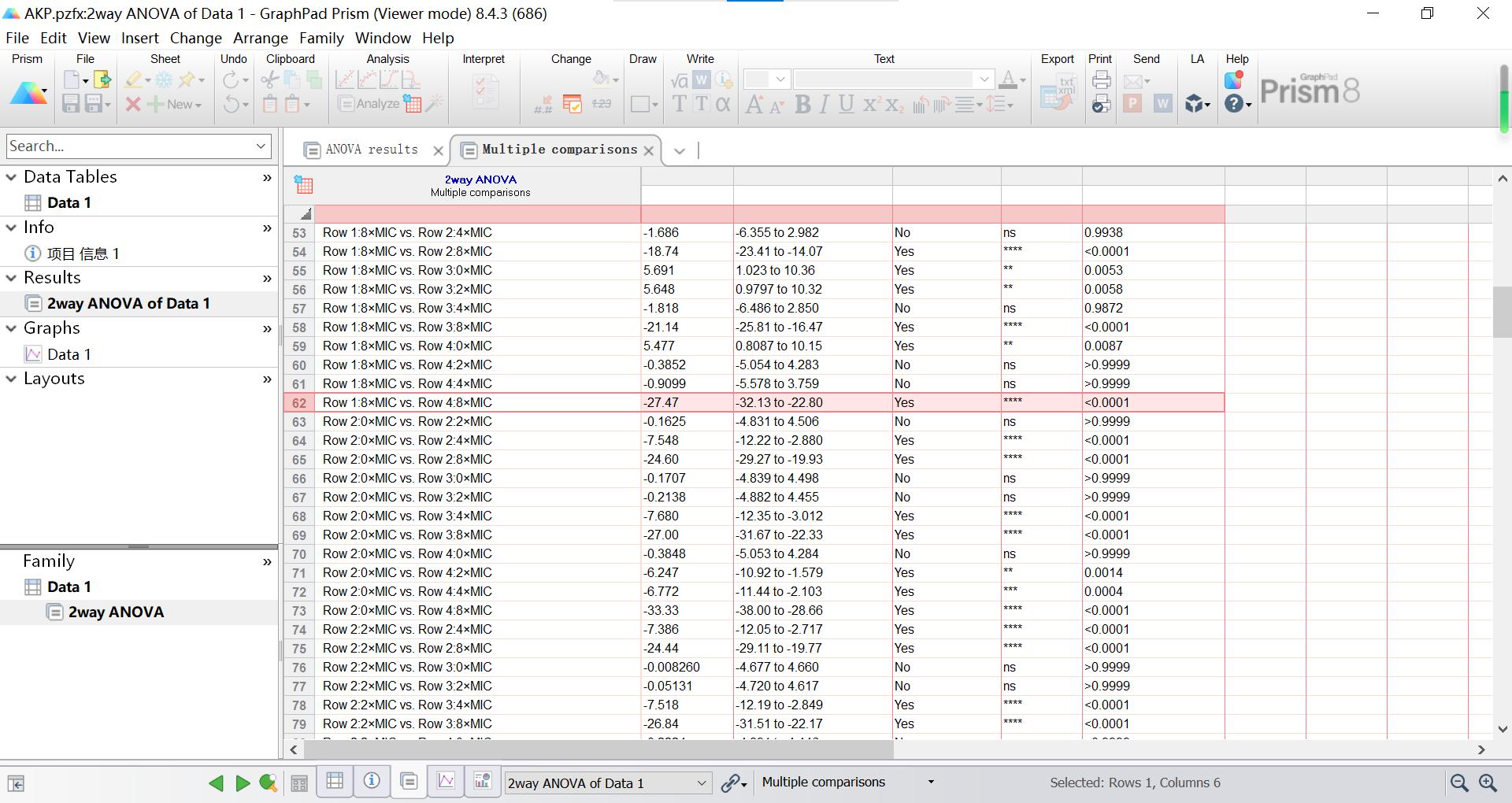

Supplement: Supplementary file 1 [file Data_Sheet_1.ZIP › Figure4-AKP Activity/screenshots of GraphPad Prism 8.0/Multiple comparisons1-3.jpg]

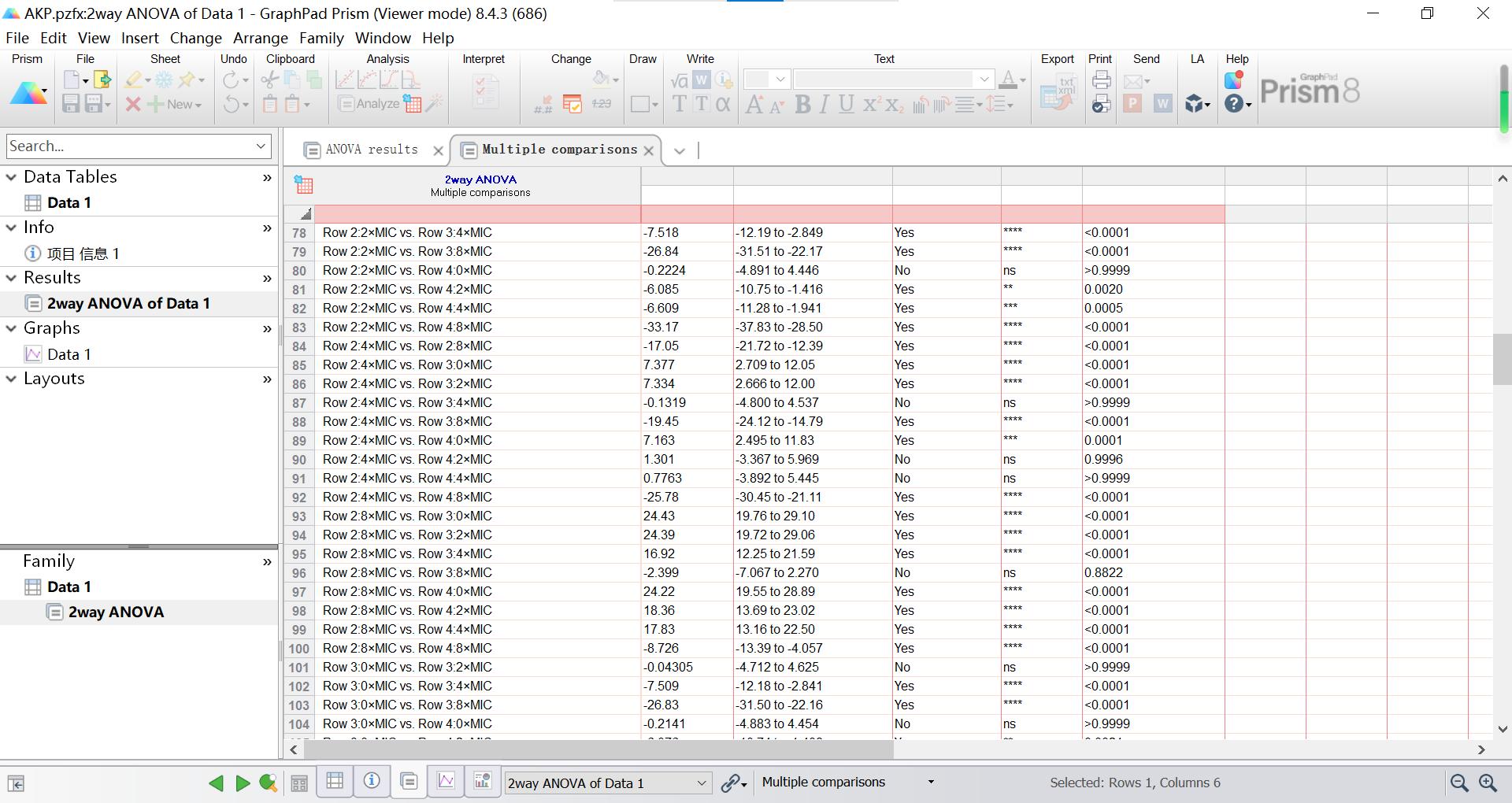

Supplement: Supplementary file 1 [file Data_Sheet_1.ZIP › Figure4-AKP Activity/screenshots of GraphPad Prism 8.0/Multiple comparisons1-4.jpg]

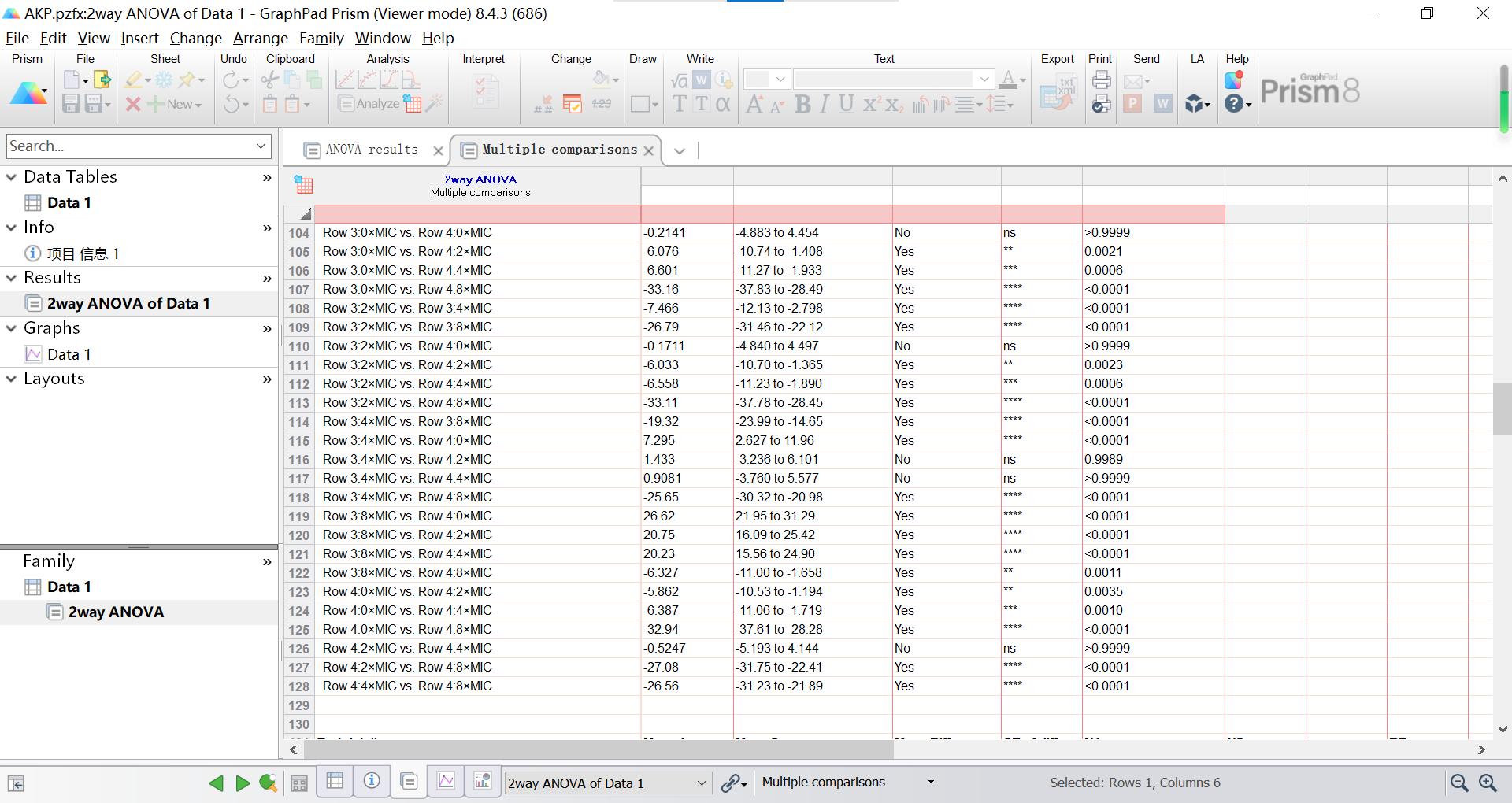

Supplement: Supplementary file 1 [file Data_Sheet_1.ZIP › Figure4-AKP Activity/screenshots of GraphPad Prism 8.0/Multiple comparisons1-5.jpg]

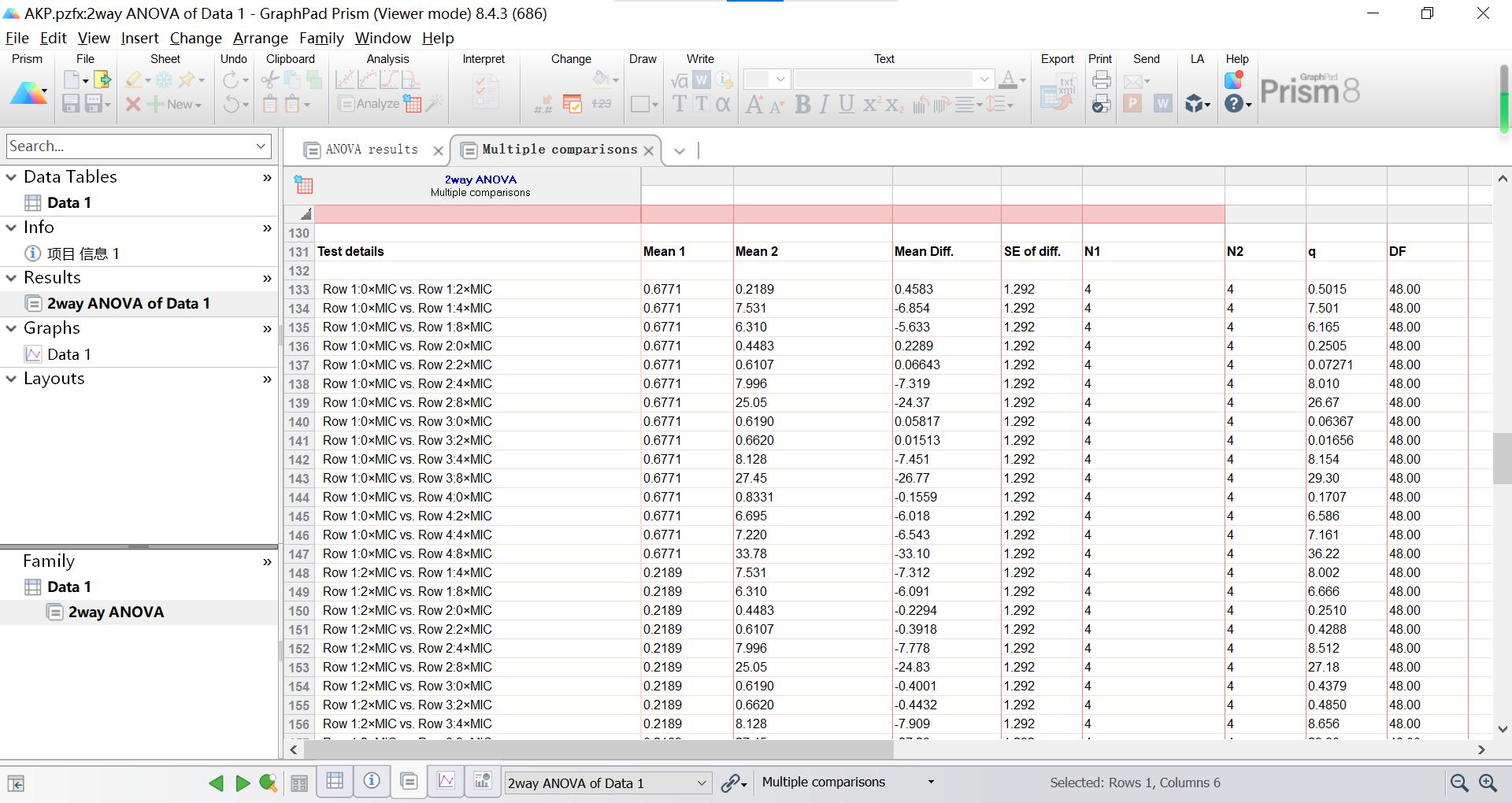

Supplement: Supplementary file 1 [file Data_Sheet_1.ZIP › Figure4-AKP Activity/screenshots of GraphPad Prism 8.0/Multiple comparisons1-6.jpg]

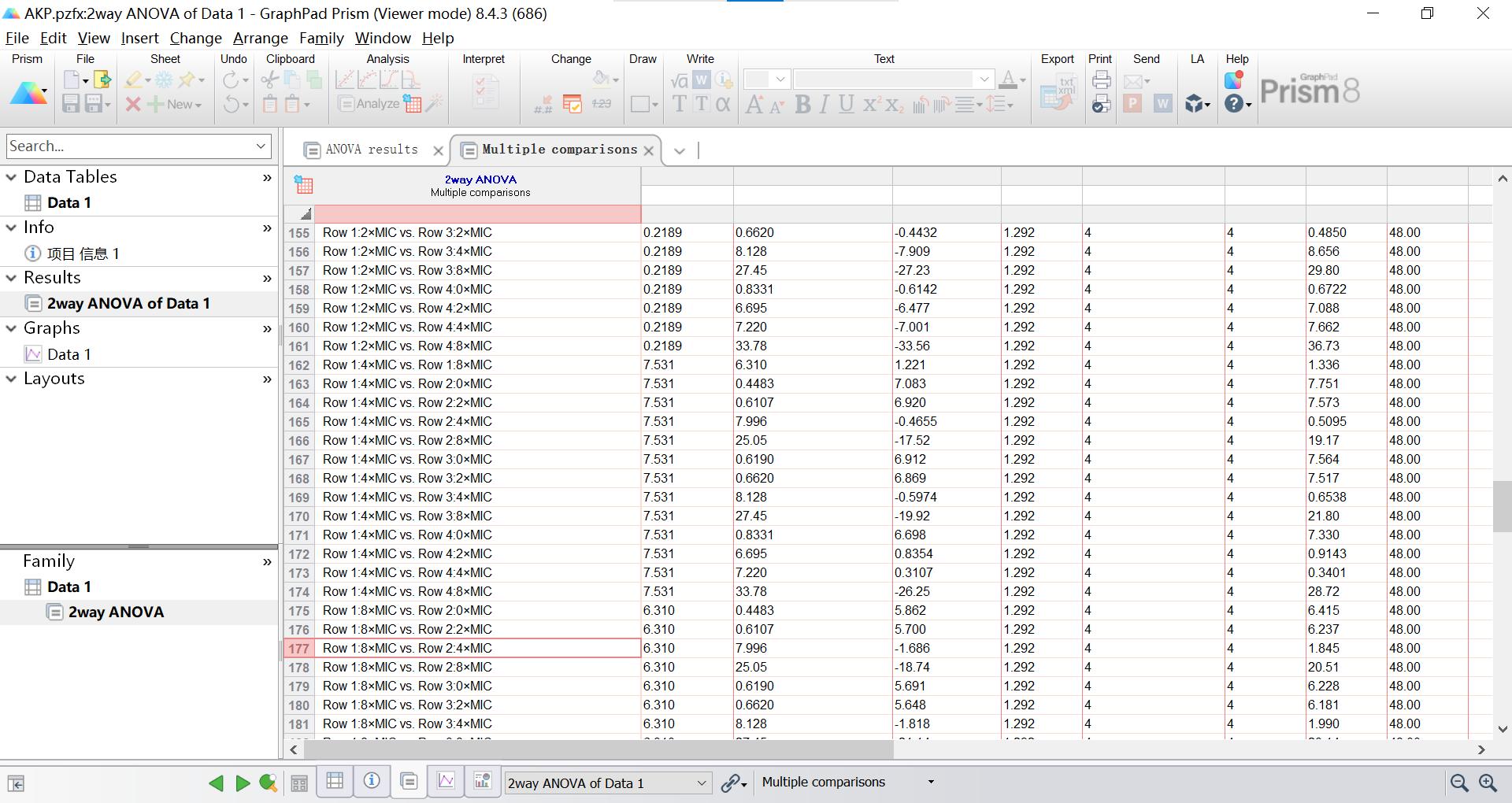

Supplement: Supplementary file 1 [file Data_Sheet_1.ZIP › Figure4-AKP Activity/screenshots of GraphPad Prism 8.0/Multiple comparisons1-7.jpg]

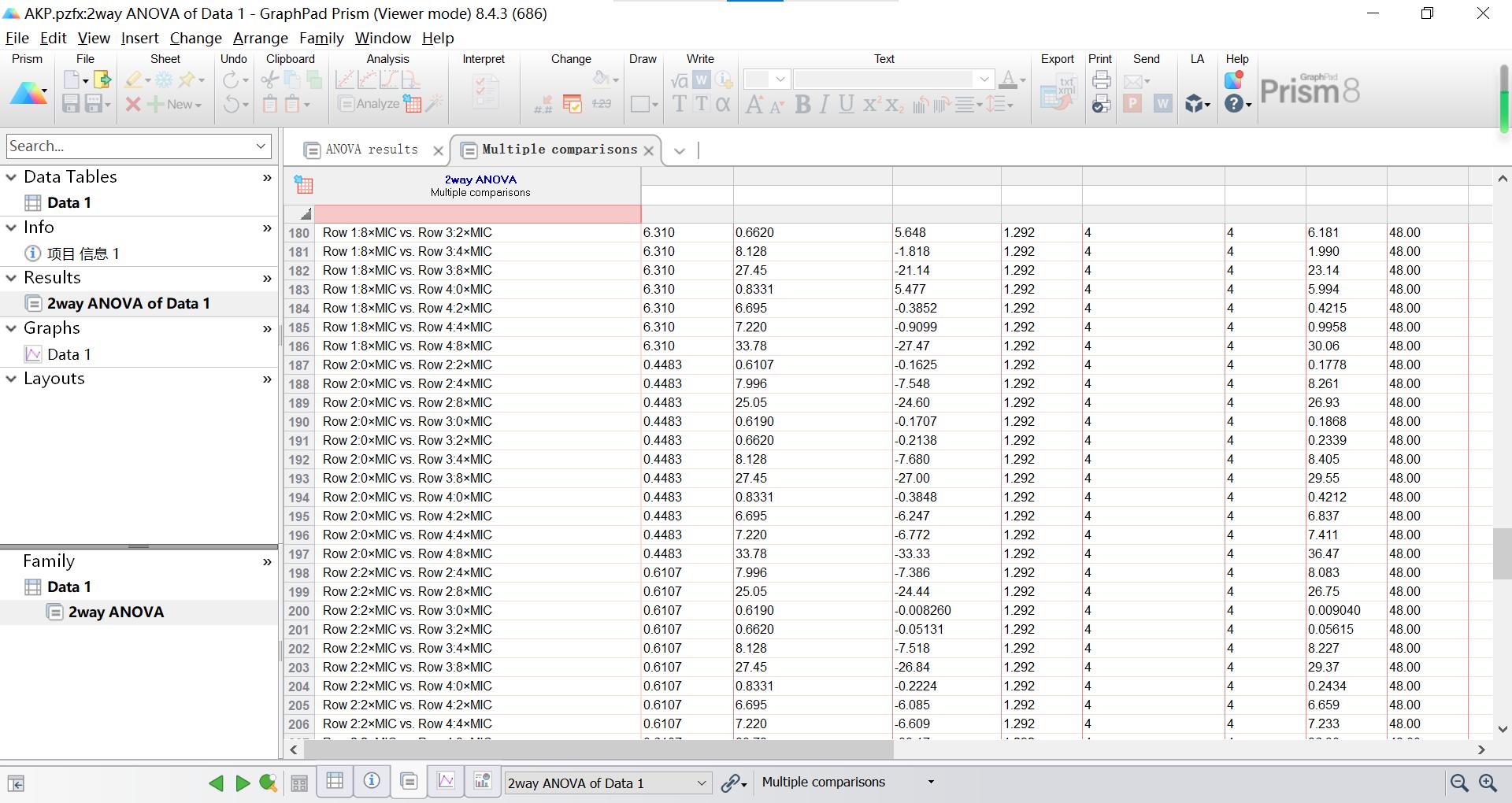

Supplement: Supplementary file 1 [file Data_Sheet_1.ZIP › Figure4-AKP Activity/screenshots of GraphPad Prism 8.0/Multiple comparisons1-8.jpg]

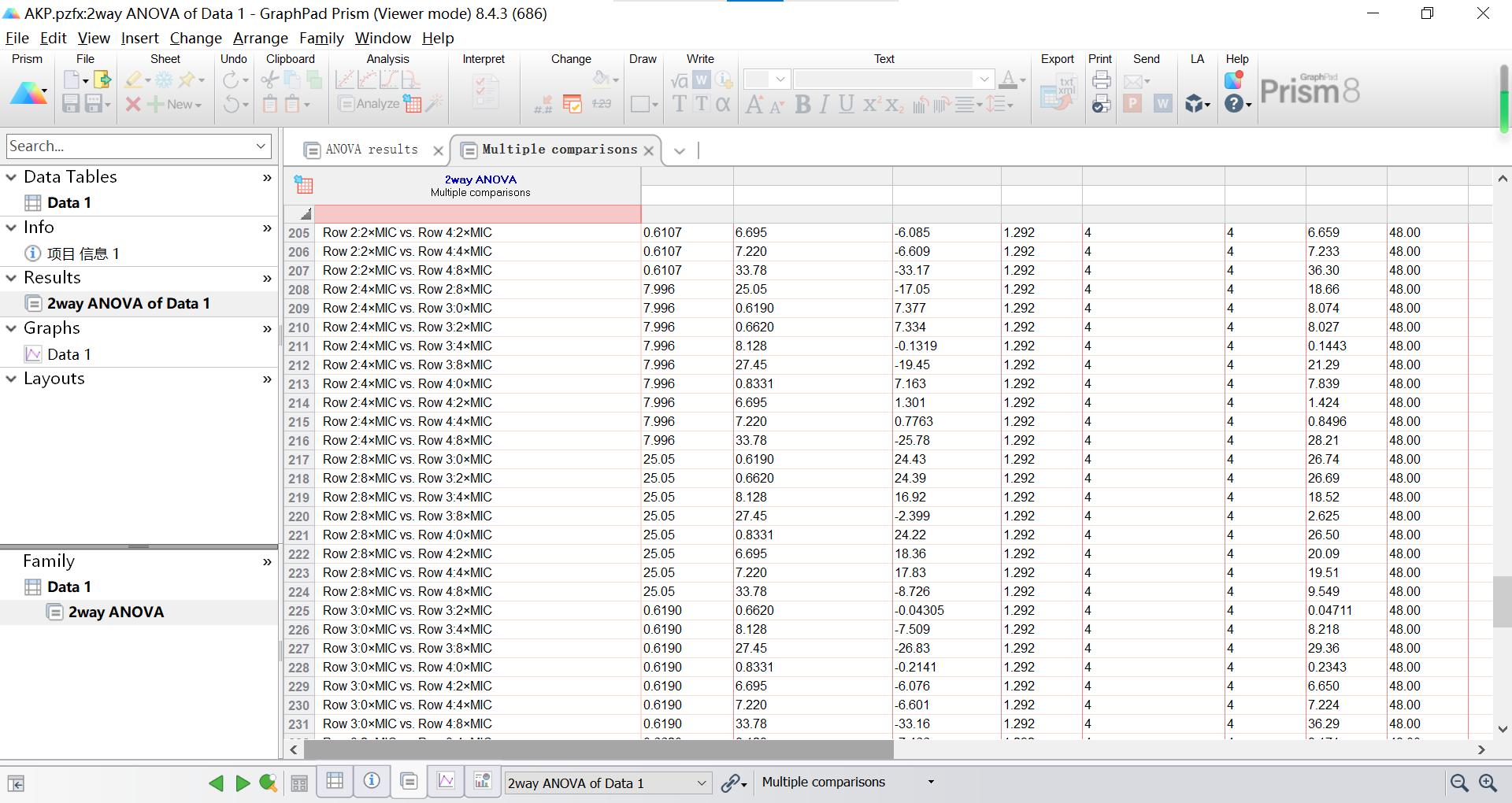

Supplement: Supplementary file 1 [file Data_Sheet_1.ZIP › Figure4-AKP Activity/screenshots of GraphPad Prism 8.0/Multiple comparisons1-9.jpg]

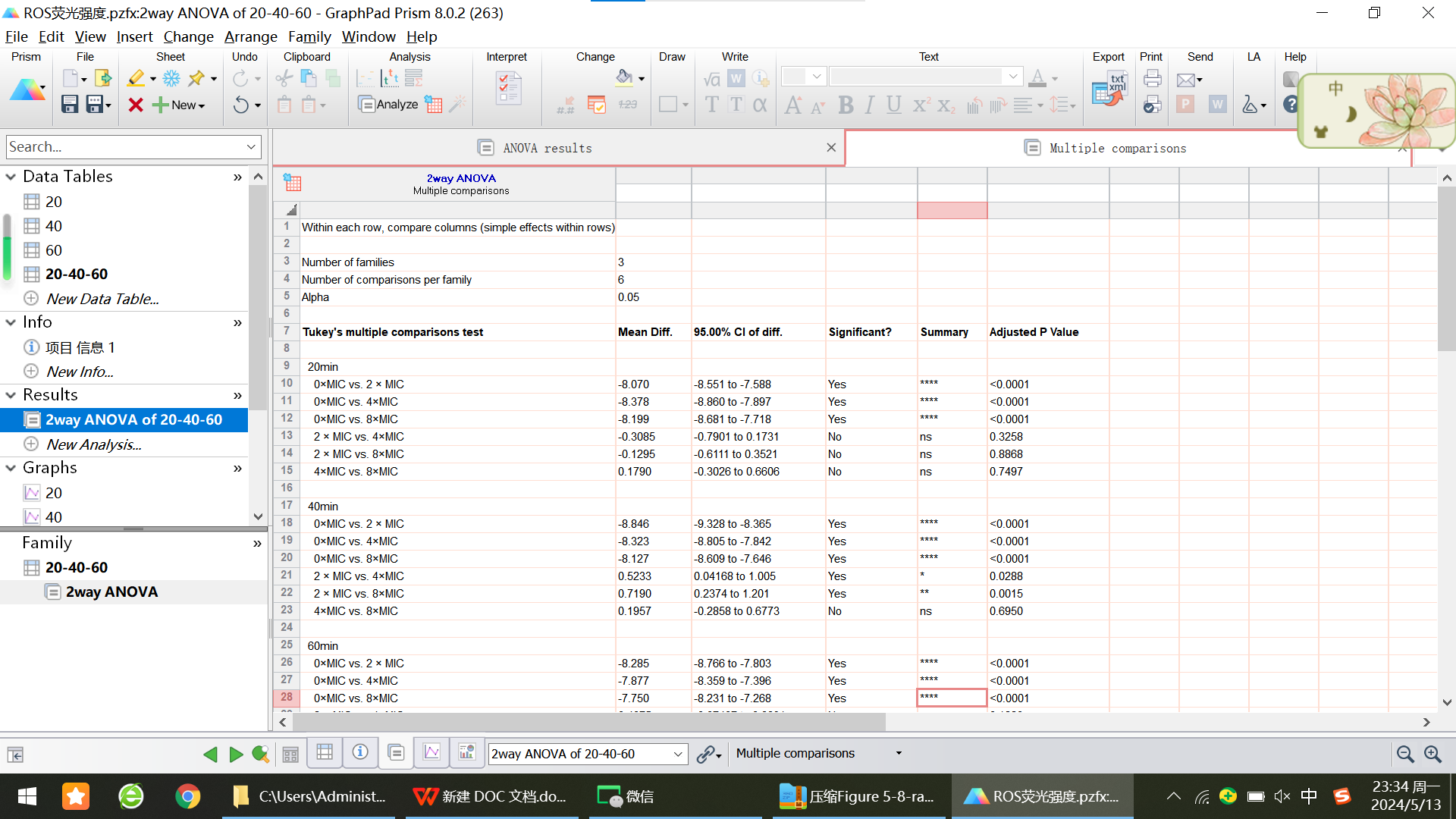

Supplement: Supplementary file 2 [file Data_Sheet_2.ZIP › Figure7-ROS/screenshots of GraphPad Prism 8.0/ROS fluorescence intensity Data/2 way ANOVN-1.png]

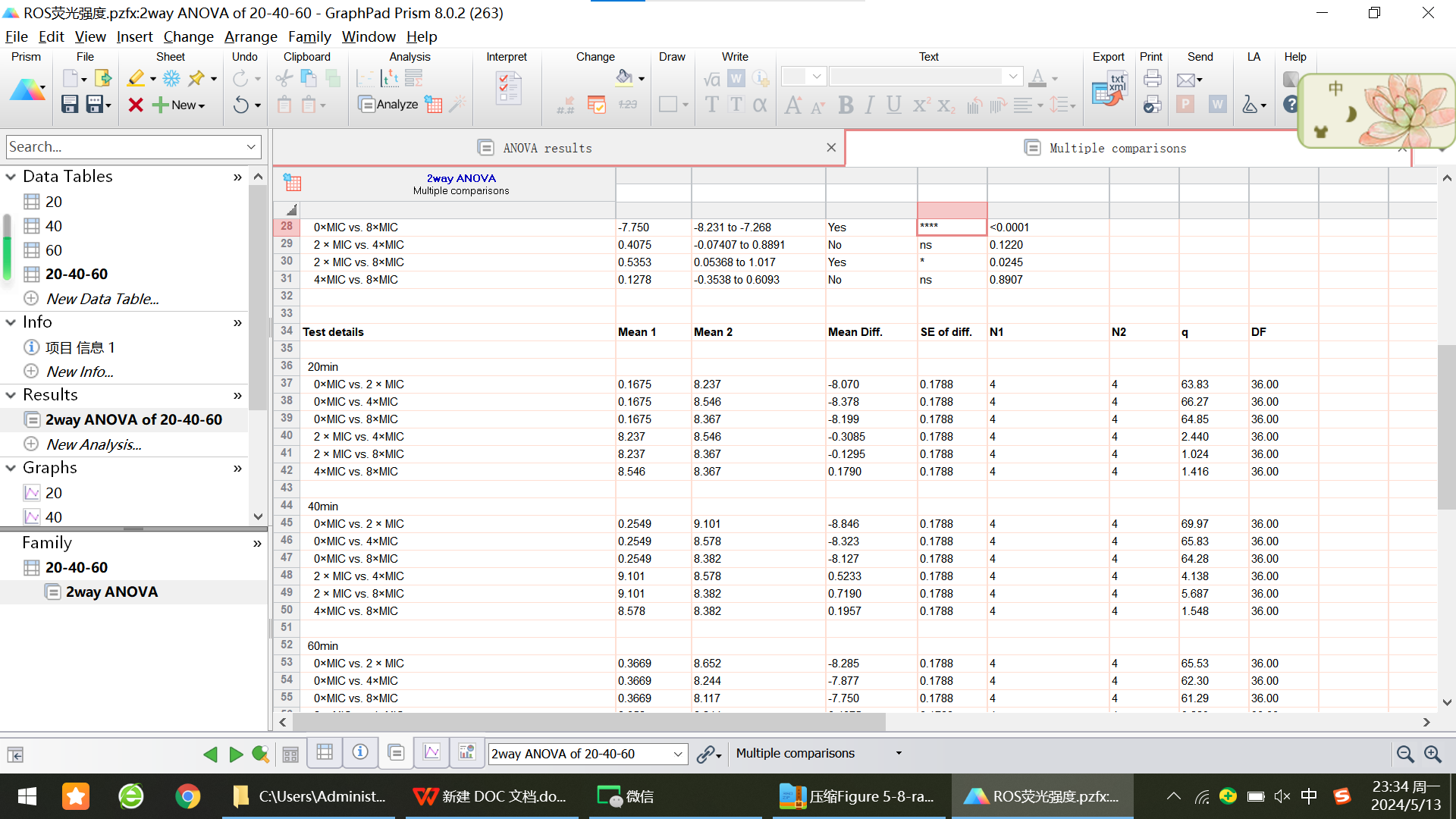

Supplement: Supplementary file 2 [file Data_Sheet_2.ZIP › Figure7-ROS/screenshots of GraphPad Prism 8.0/ROS fluorescence intensity Data/2 way ANOVN-2.png]

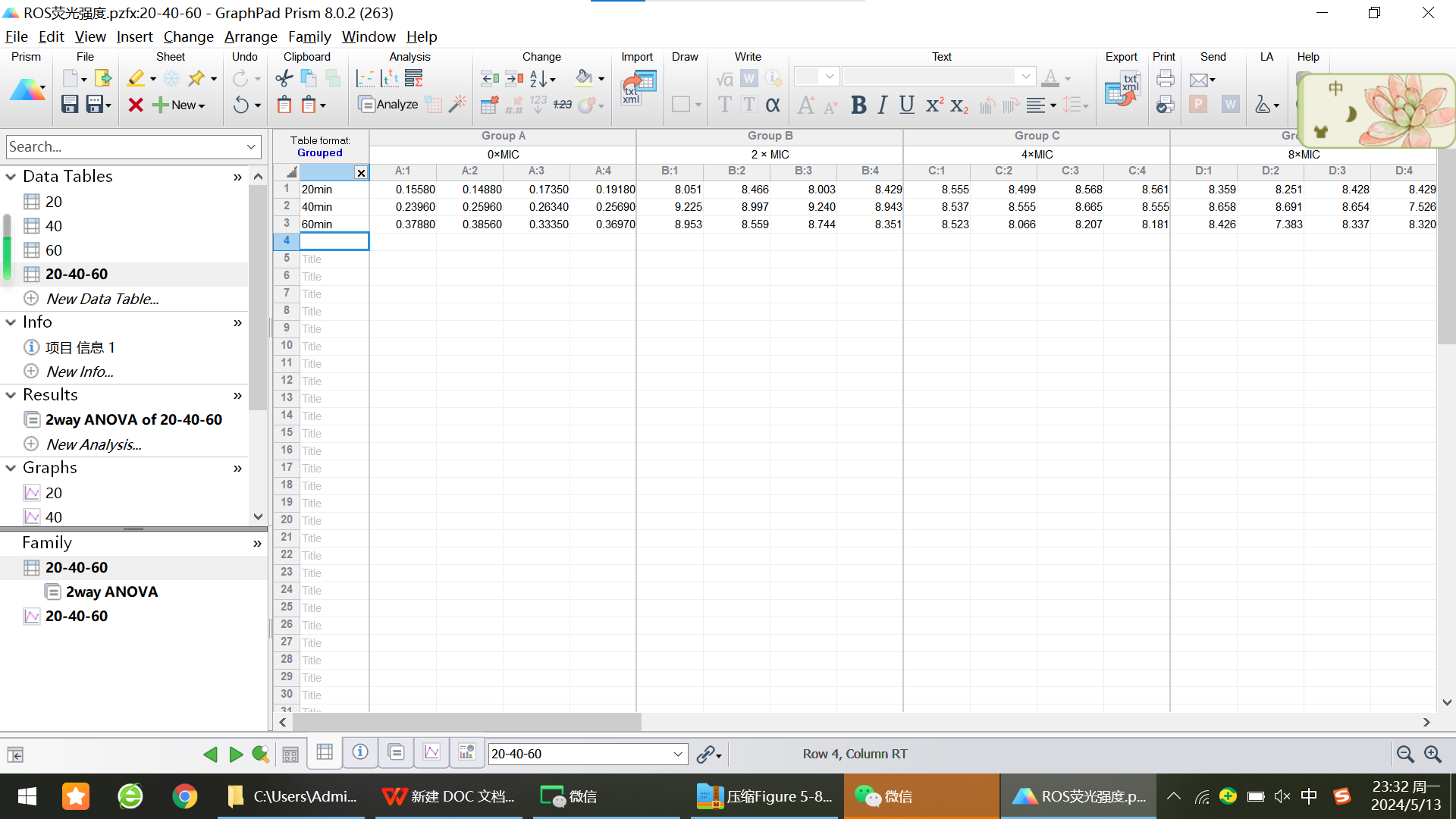

Supplement: Supplementary file 2 [file Data_Sheet_2.ZIP › Figure7-ROS/screenshots of GraphPad Prism 8.0/ROS fluorescence intensity Data/ROS.png]

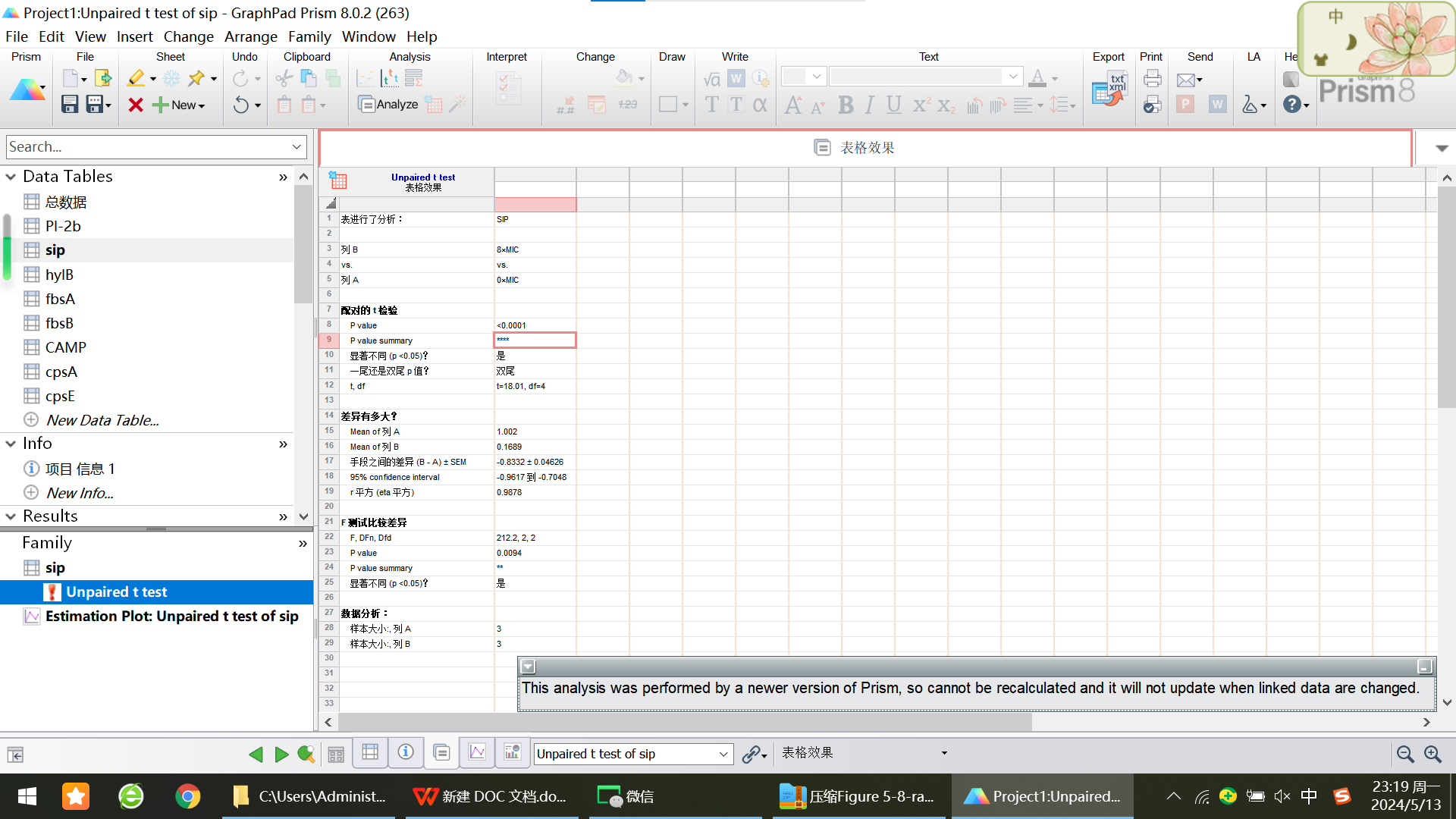

Supplement: Supplementary file 2 [file Data_Sheet_2.ZIP › Figure8-qRT-PCR/screenshots of GraphPad Prism 8.0/qp/sip/t test.png]

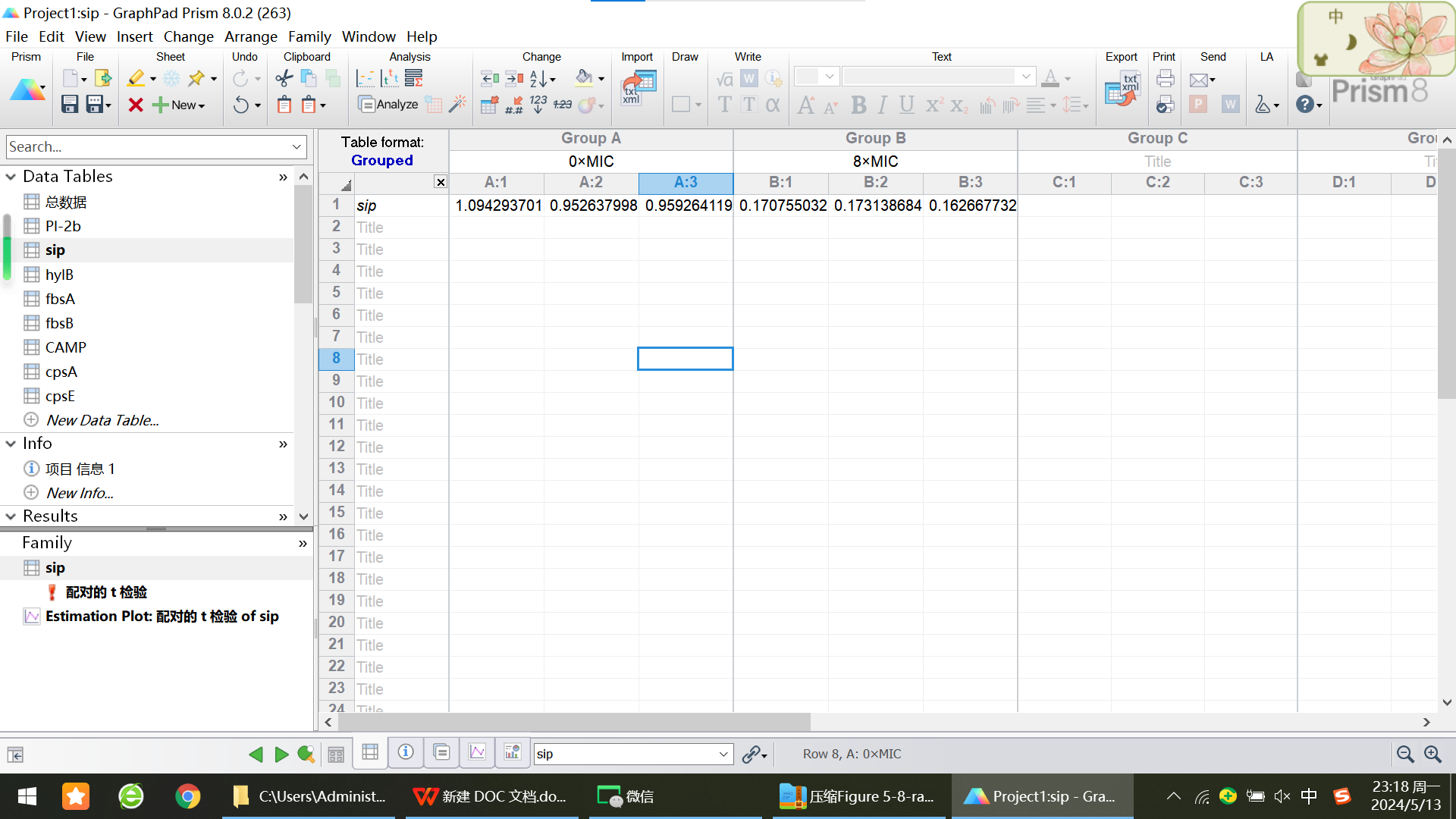

Supplement: Supplementary file 2 [file Data_Sheet_2.ZIP › Figure8-qRT-PCR/screenshots of GraphPad Prism 8.0/qp/sip/sip.png]

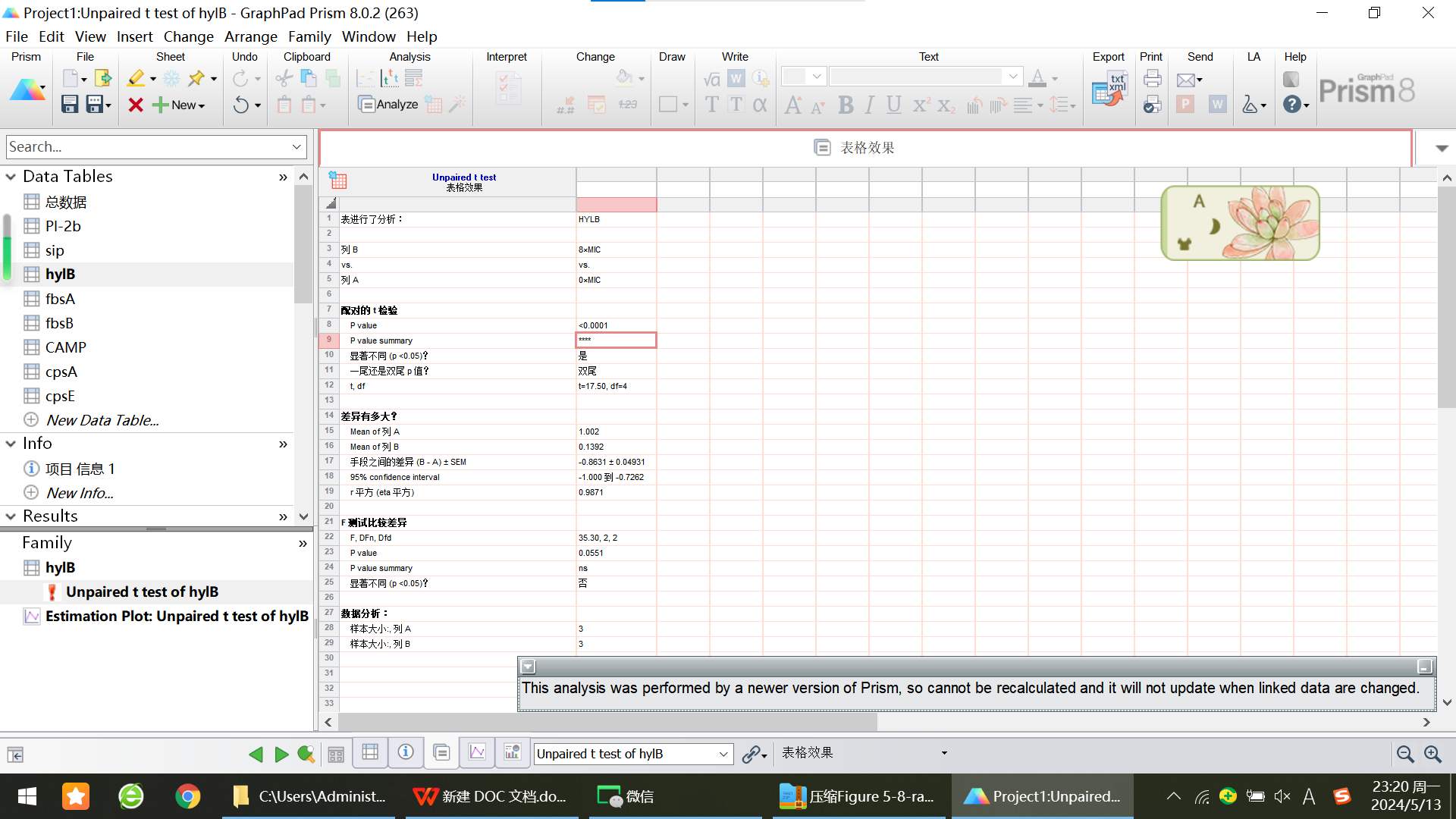

Supplement: Supplementary file 2 [file Data_Sheet_2.ZIP › Figure8-qRT-PCR/screenshots of GraphPad Prism 8.0/qp/hylB/t test.png]

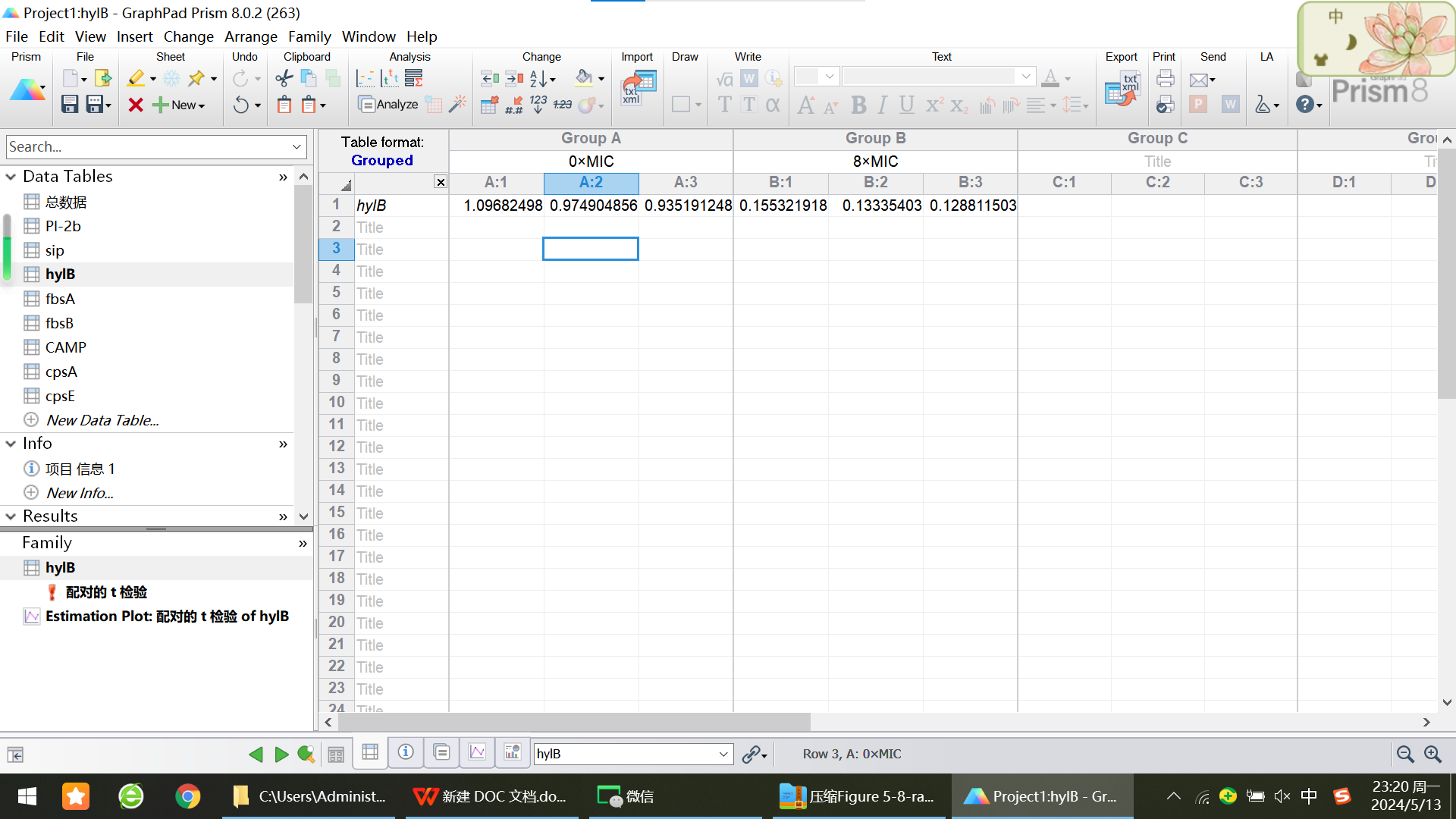

Supplement: Supplementary file 2 [file Data_Sheet_2.ZIP › Figure8-qRT-PCR/screenshots of GraphPad Prism 8.0/qp/hylB/HylB.png]

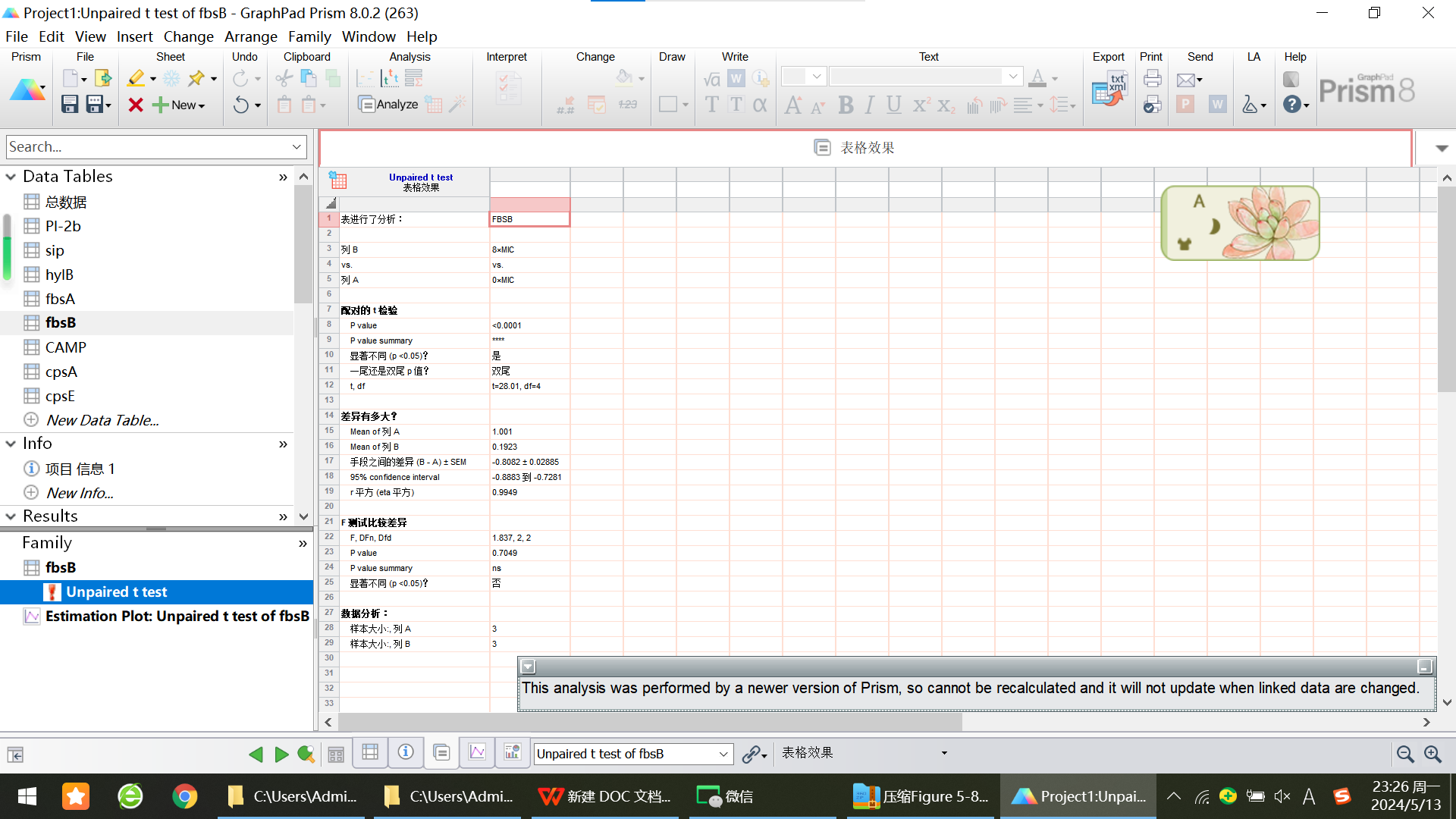

Supplement: Supplementary file 2 [file Data_Sheet_2.ZIP › Figure8-qRT-PCR/screenshots of GraphPad Prism 8.0/qp/fbsB/t test.png]

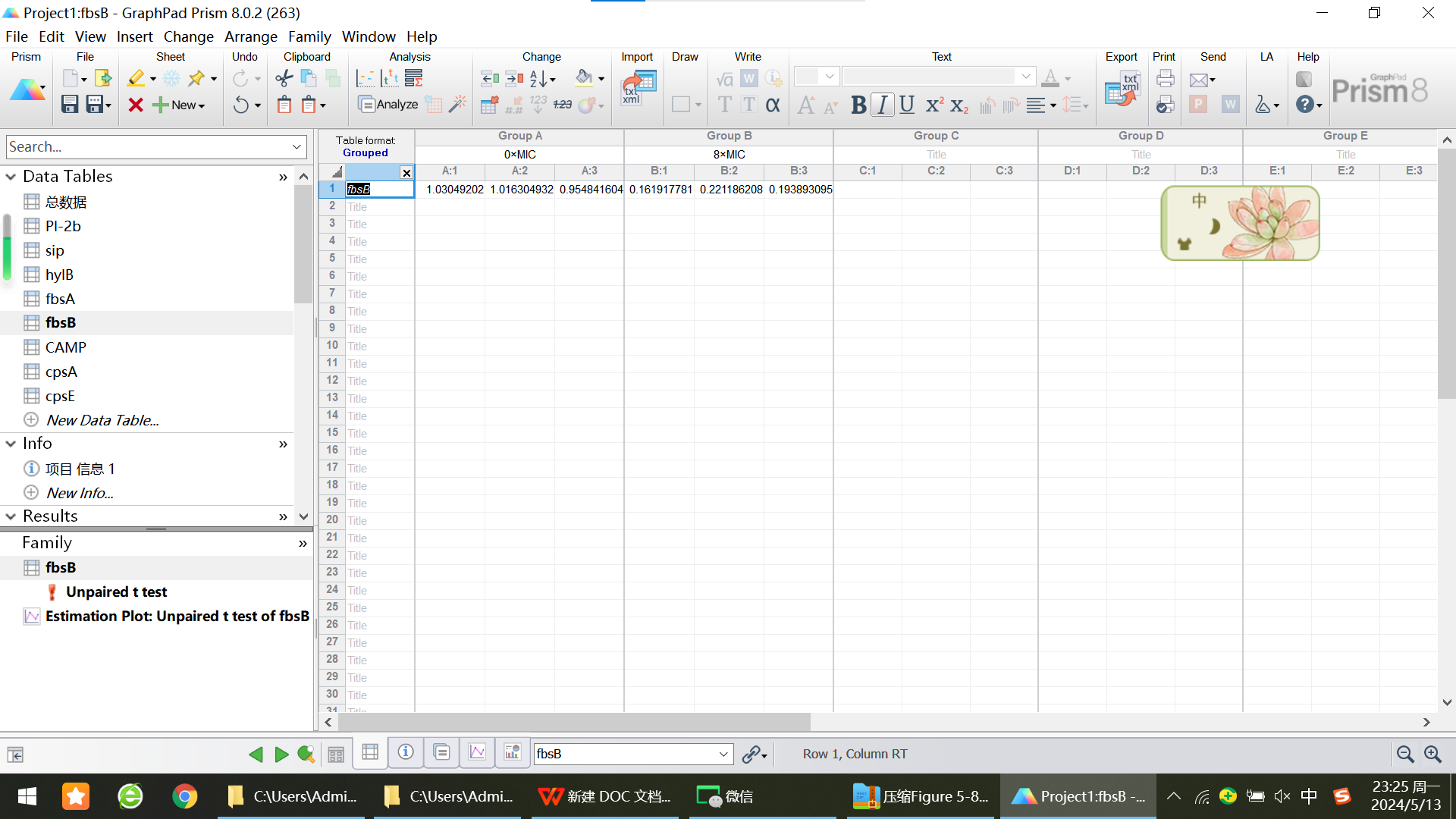

Supplement: Supplementary file 2 [file Data_Sheet_2.ZIP › Figure8-qRT-PCR/screenshots of GraphPad Prism 8.0/qp/fbsB/fbsB.png]

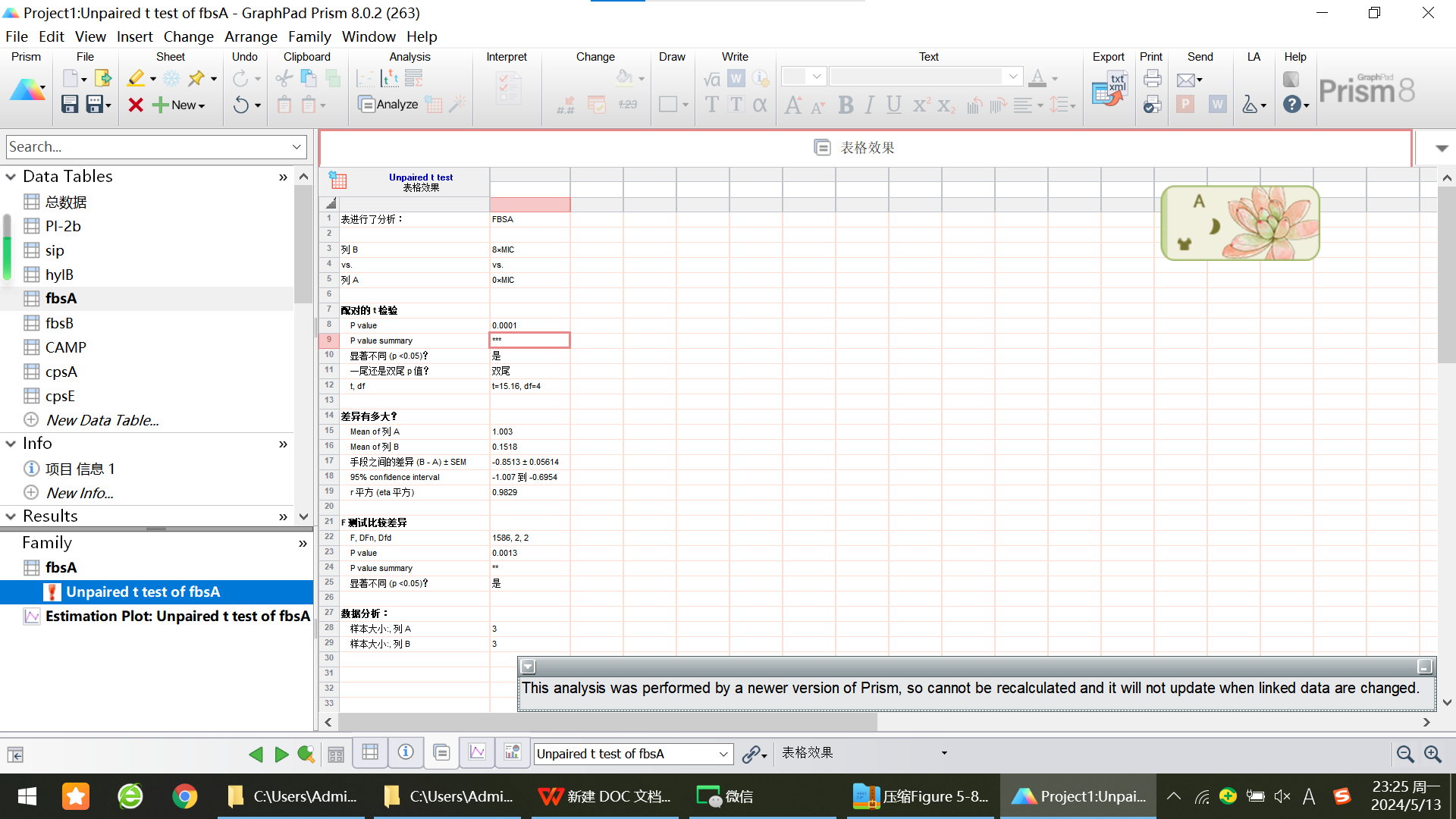

Supplement: Supplementary file 2 [file Data_Sheet_2.ZIP › Figure8-qRT-PCR/screenshots of GraphPad Prism 8.0/qp/fbsA/t test.png]

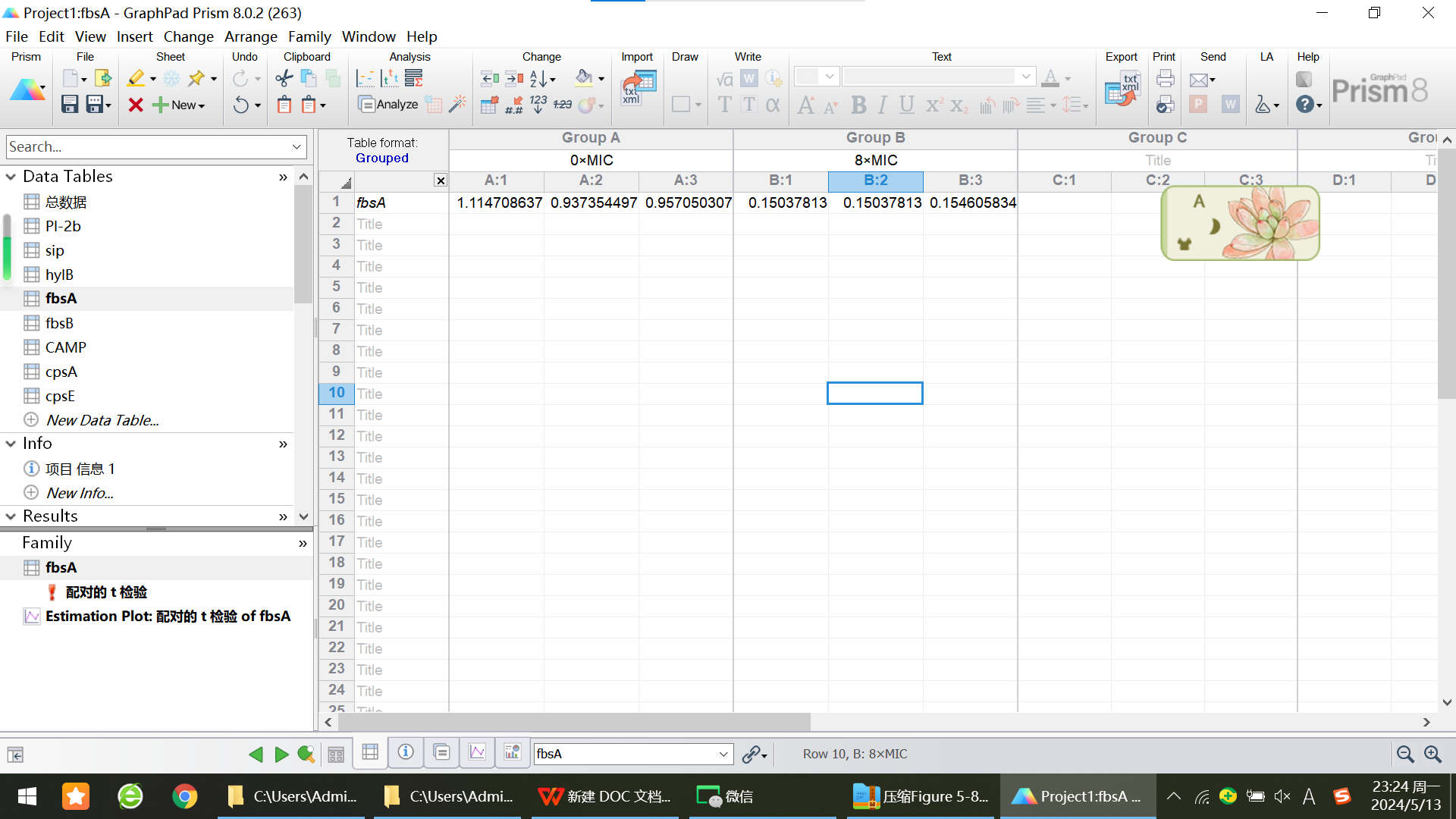

Supplement: Supplementary file 2 [file Data_Sheet_2.ZIP › Figure8-qRT-PCR/screenshots of GraphPad Prism 8.0/qp/fbsA/fbsA.png]

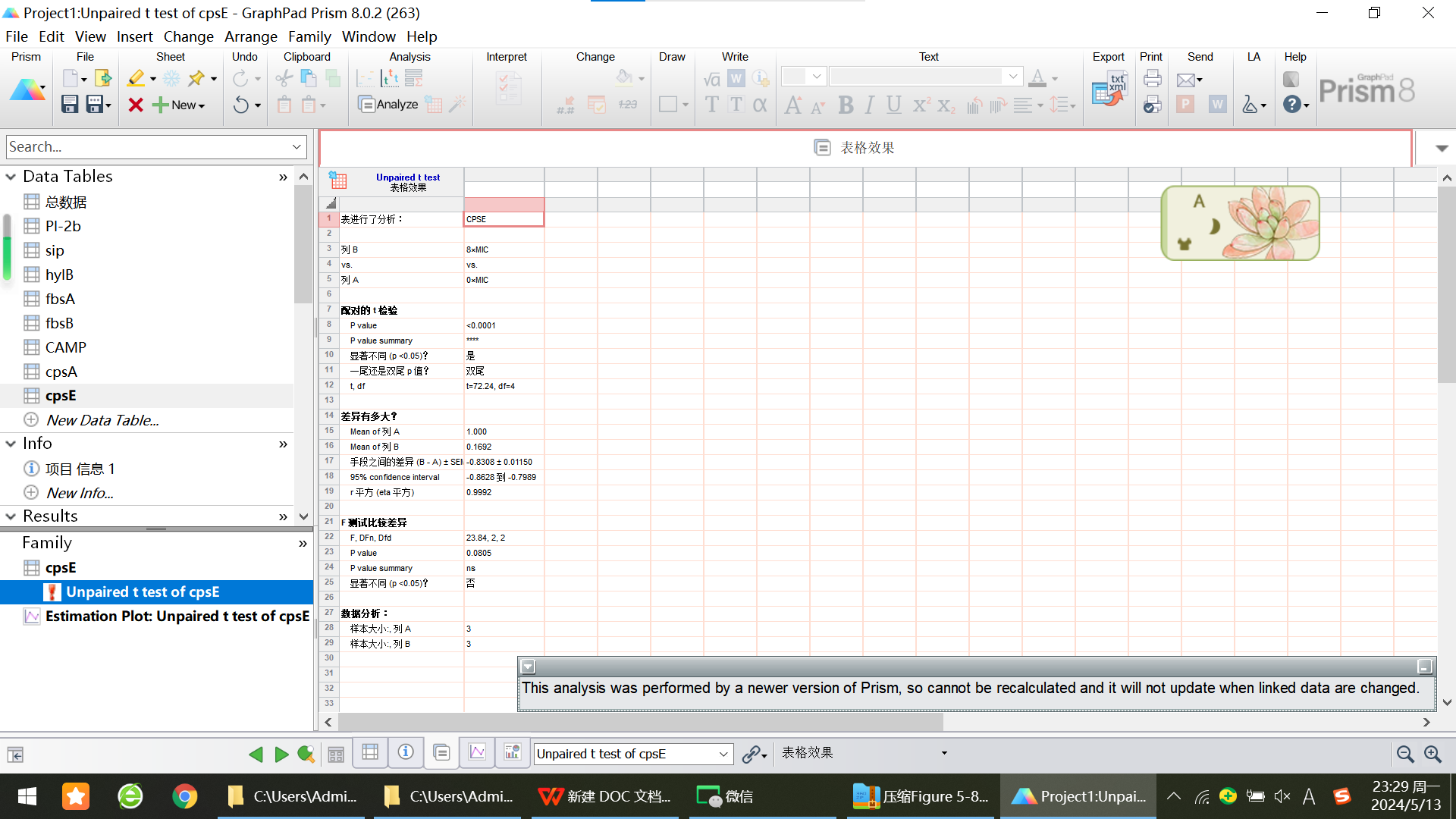

Supplement: Supplementary file 2 [file Data_Sheet_2.ZIP › Figure8-qRT-PCR/screenshots of GraphPad Prism 8.0/qp/cpsE/t test.png]

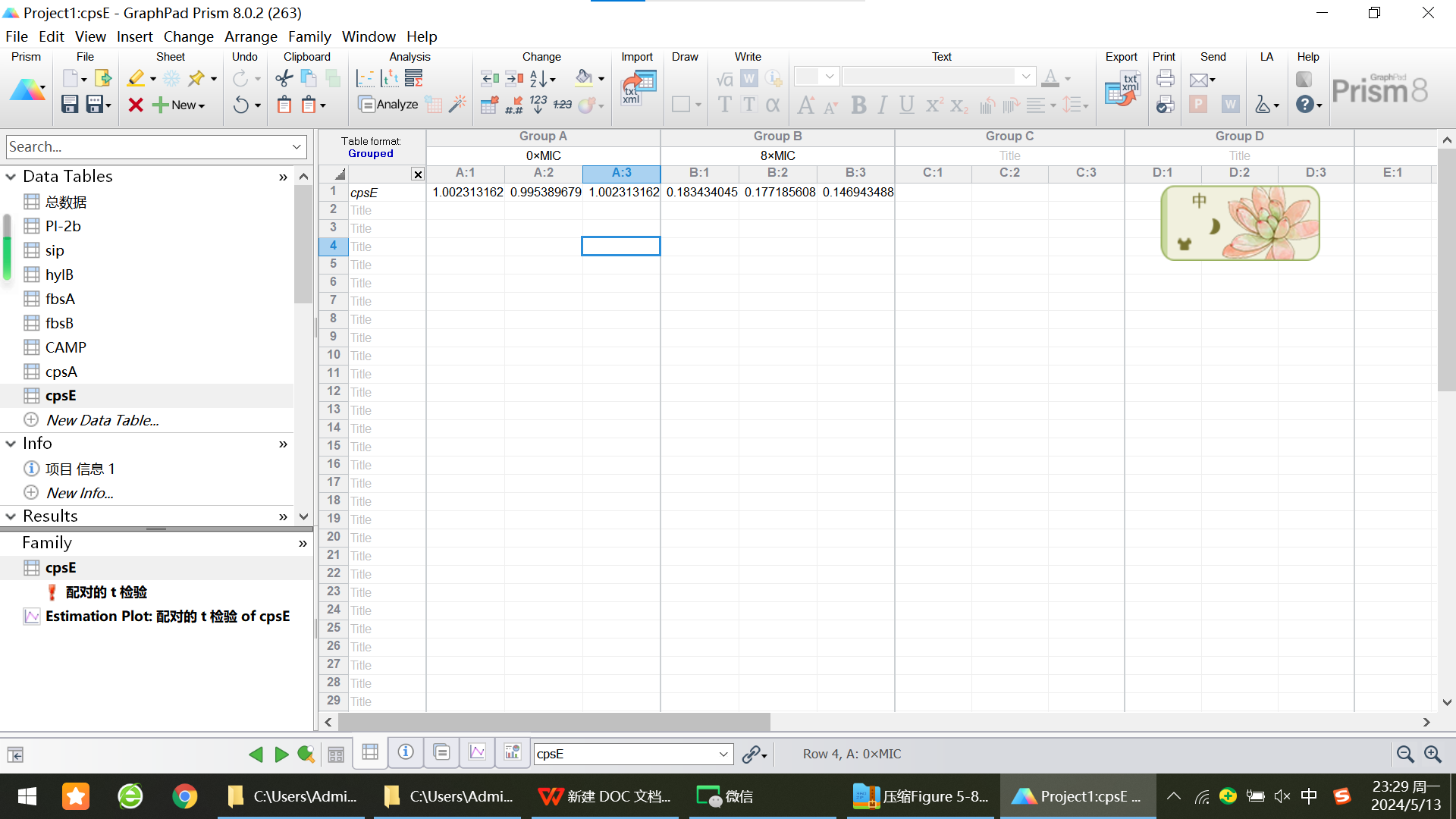

Supplement: Supplementary file 2 [file Data_Sheet_2.ZIP › Figure8-qRT-PCR/screenshots of GraphPad Prism 8.0/qp/cpsE/cspE.png]

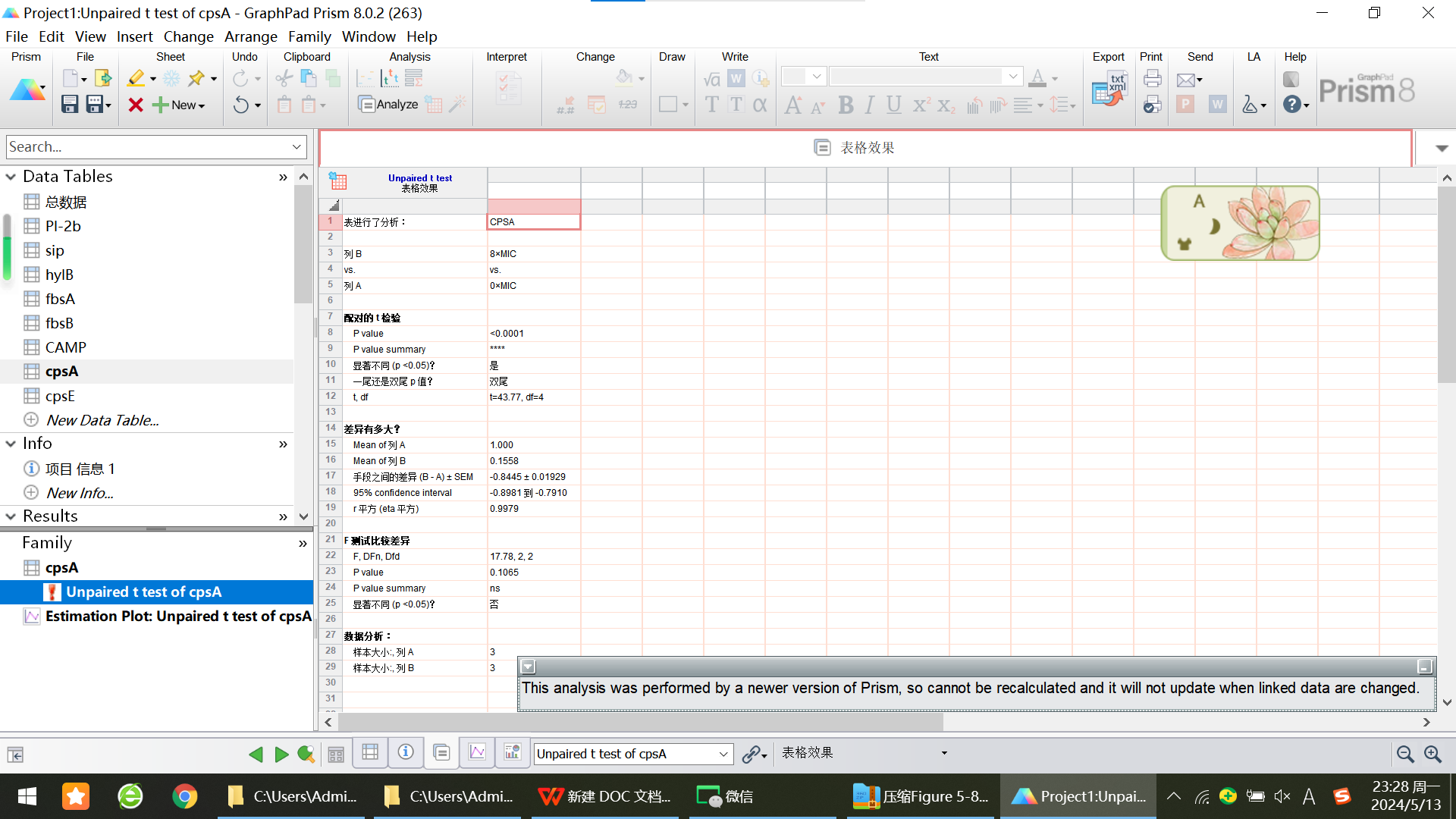

Supplement: Supplementary file 2 [file Data_Sheet_2.ZIP › Figure8-qRT-PCR/screenshots of GraphPad Prism 8.0/qp/cpsA/t test.png]

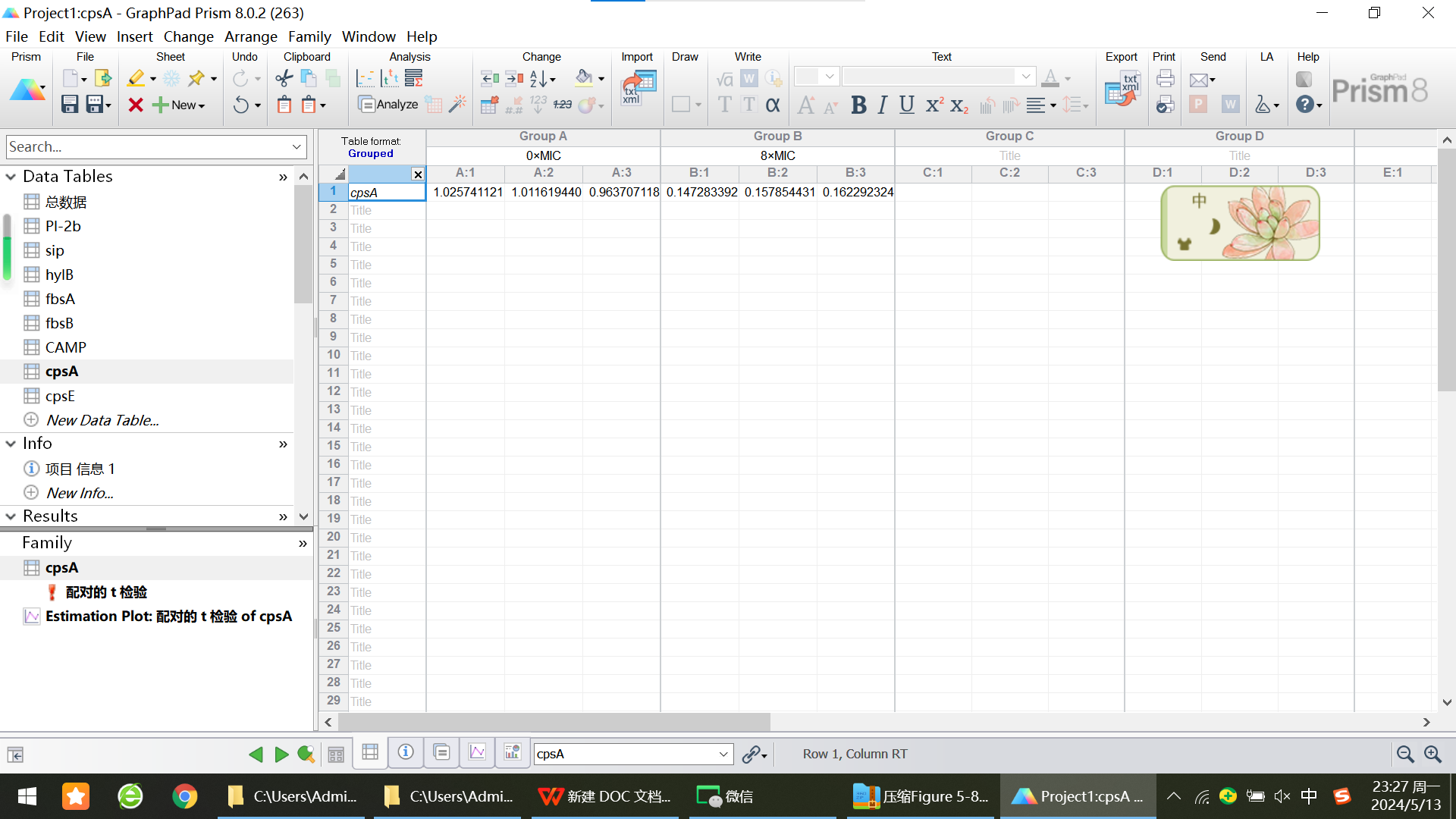

Supplement: Supplementary file 2 [file Data_Sheet_2.ZIP › Figure8-qRT-PCR/screenshots of GraphPad Prism 8.0/qp/cpsA/cpsA.png]

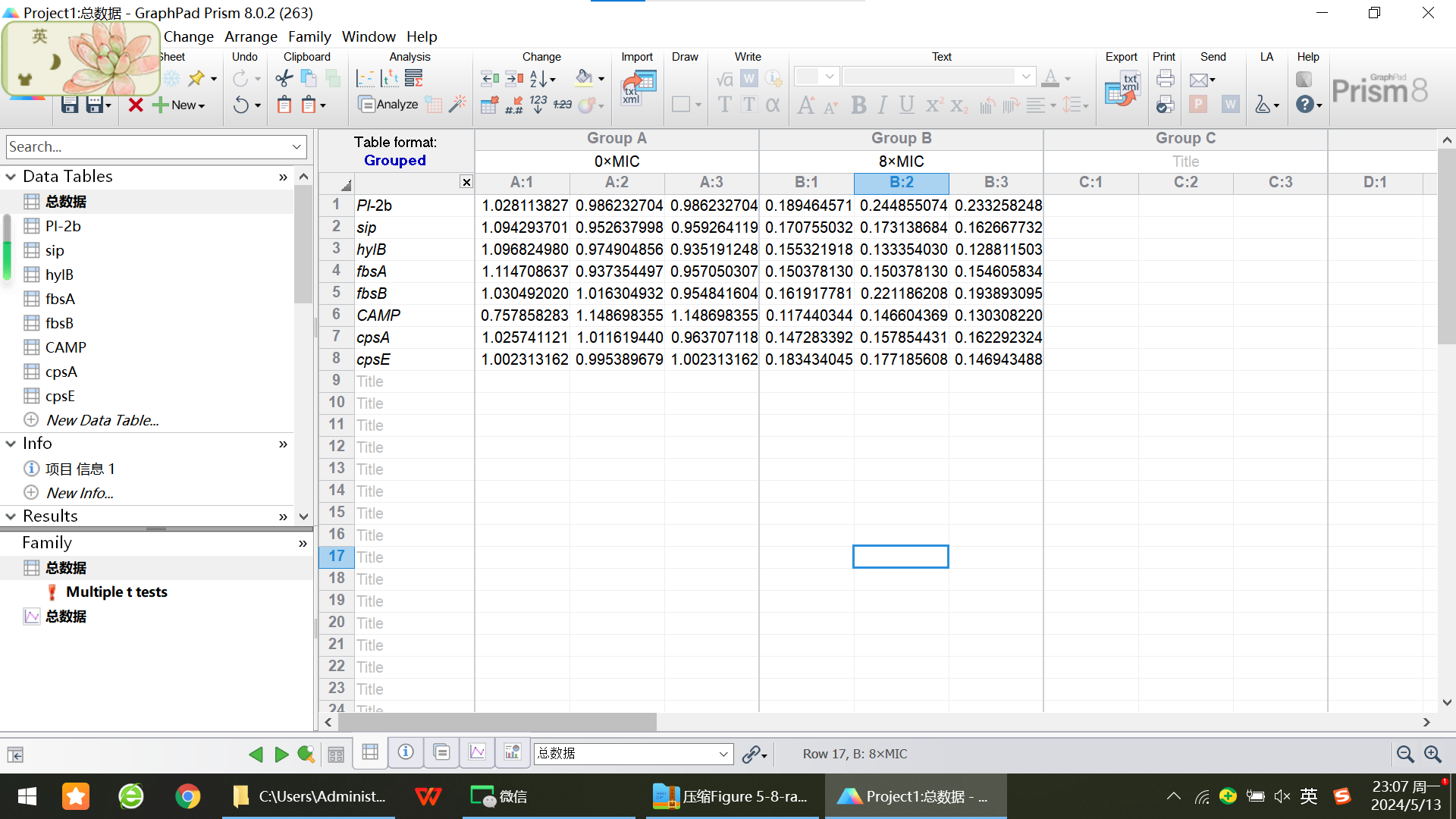

Supplement: Supplementary file 2 [file Data_Sheet_2.ZIP › Figure8-qRT-PCR/screenshots of GraphPad Prism 8.0/qp/all genes/raw data.png]

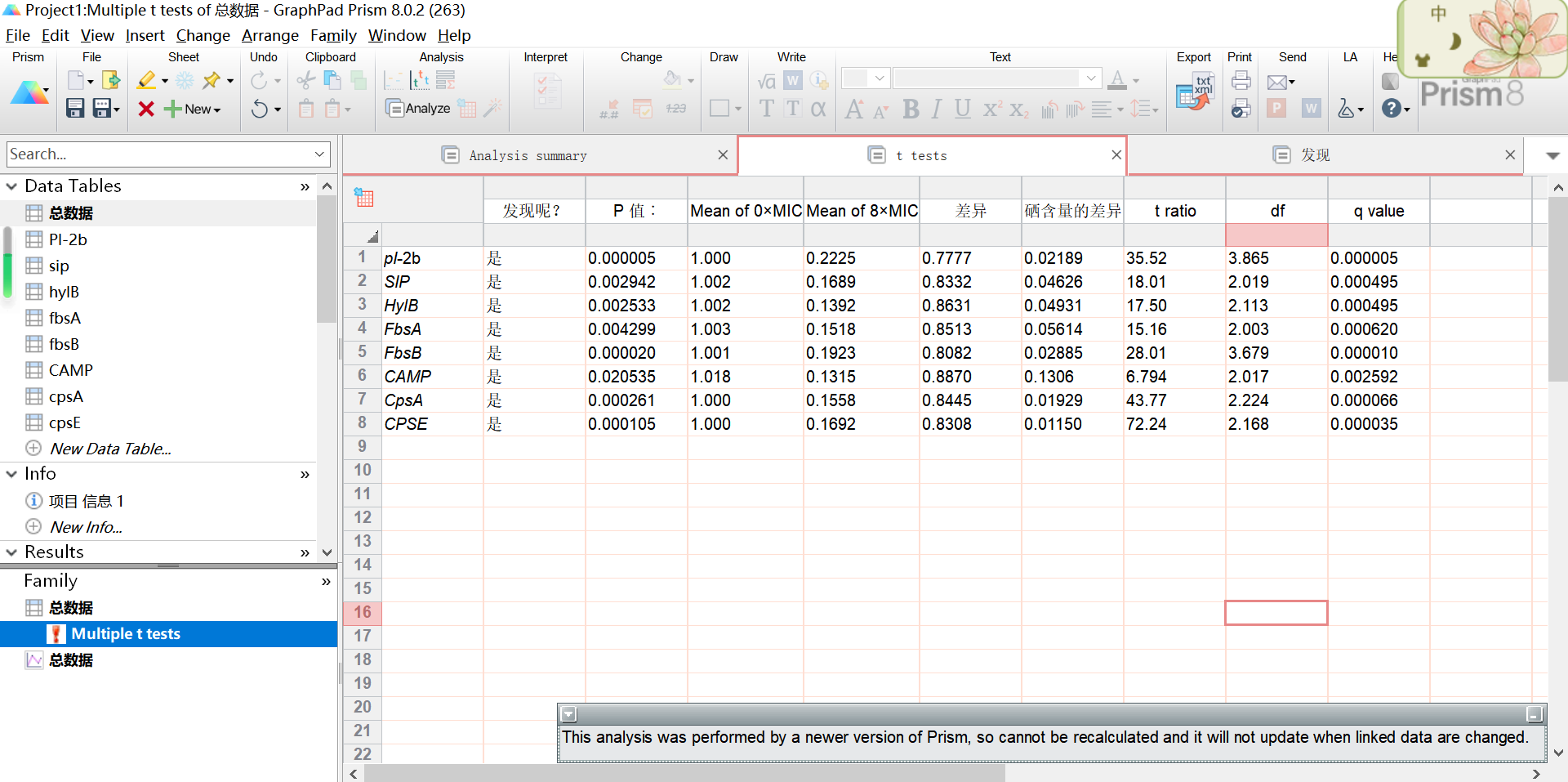

Supplement: Supplementary file 2 [file Data_Sheet_2.ZIP › Figure8-qRT-PCR/screenshots of GraphPad Prism 8.0/qp/all genes/Multiple t test.png]

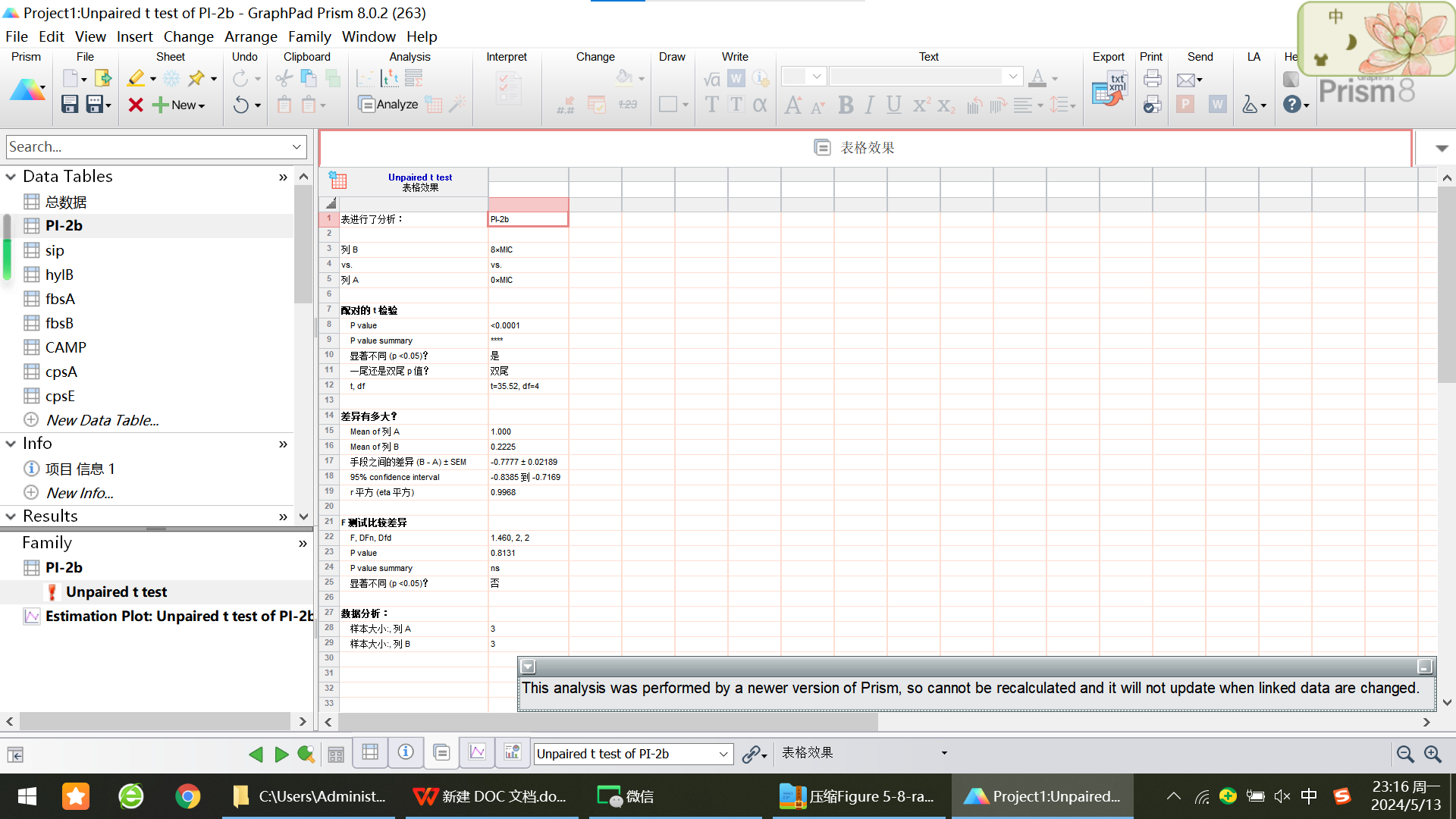

Supplement: Supplementary file 2 [file Data_Sheet_2.ZIP › Figure8-qRT-PCR/screenshots of GraphPad Prism 8.0/qp/PI-2b/t test.png]

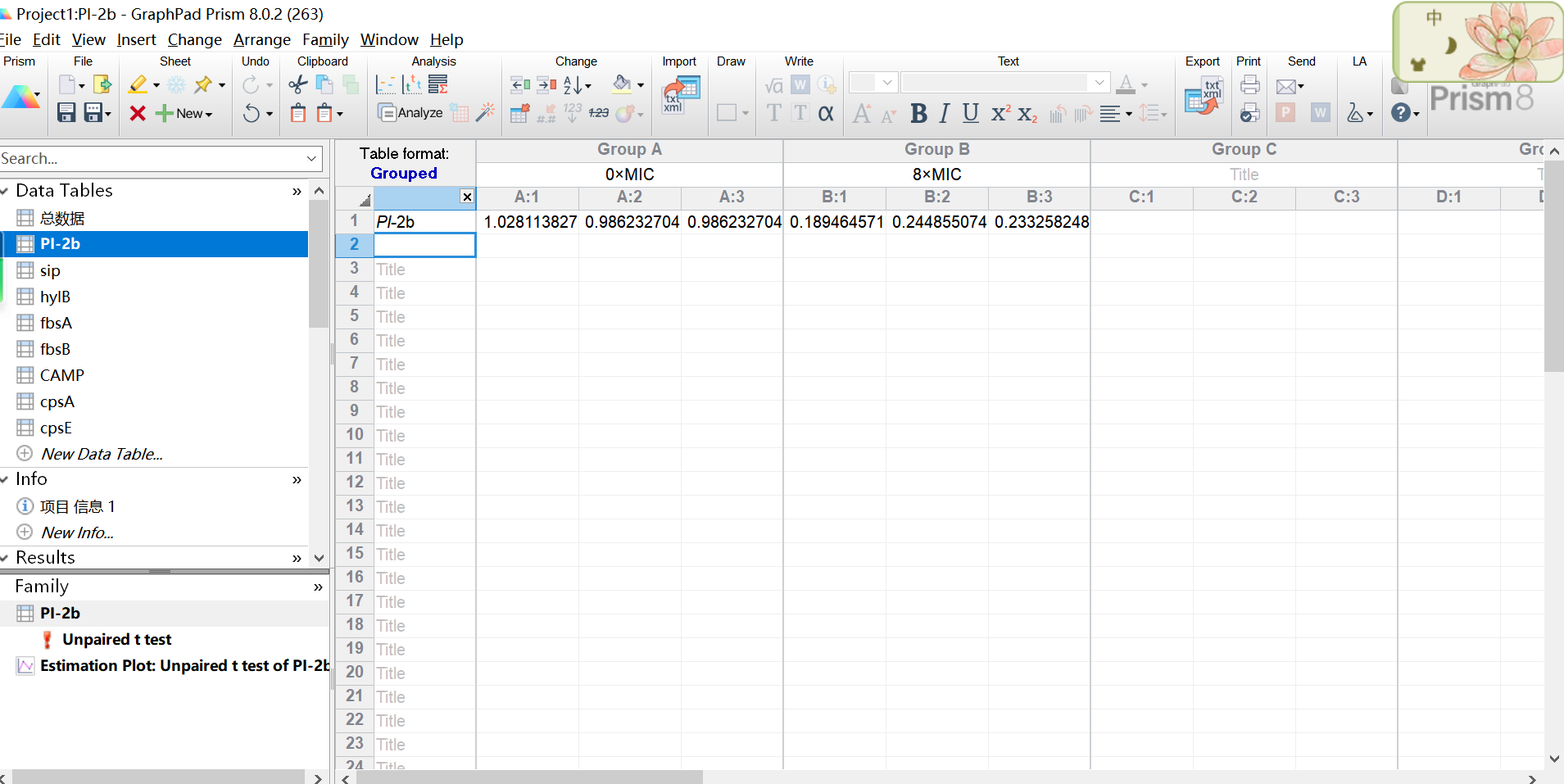

Supplement: Supplementary file 2 [file Data_Sheet_2.ZIP › Figure8-qRT-PCR/screenshots of GraphPad Prism 8.0/qp/PI-2b/PI-2b.png]

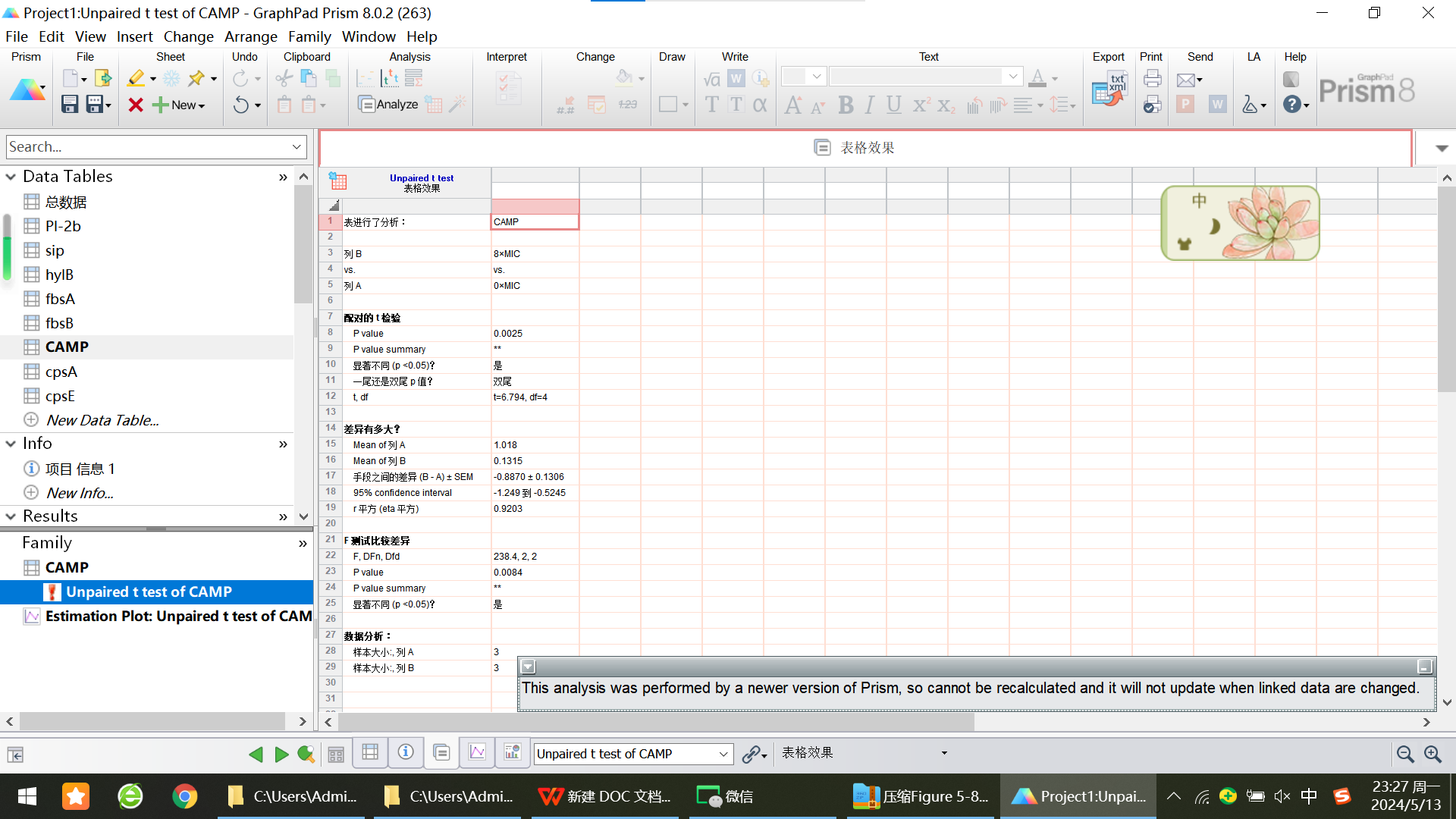

Supplement: Supplementary file 2 [file Data_Sheet_2.ZIP › Figure8-qRT-PCR/screenshots of GraphPad Prism 8.0/qp/CAMP/t test.png]

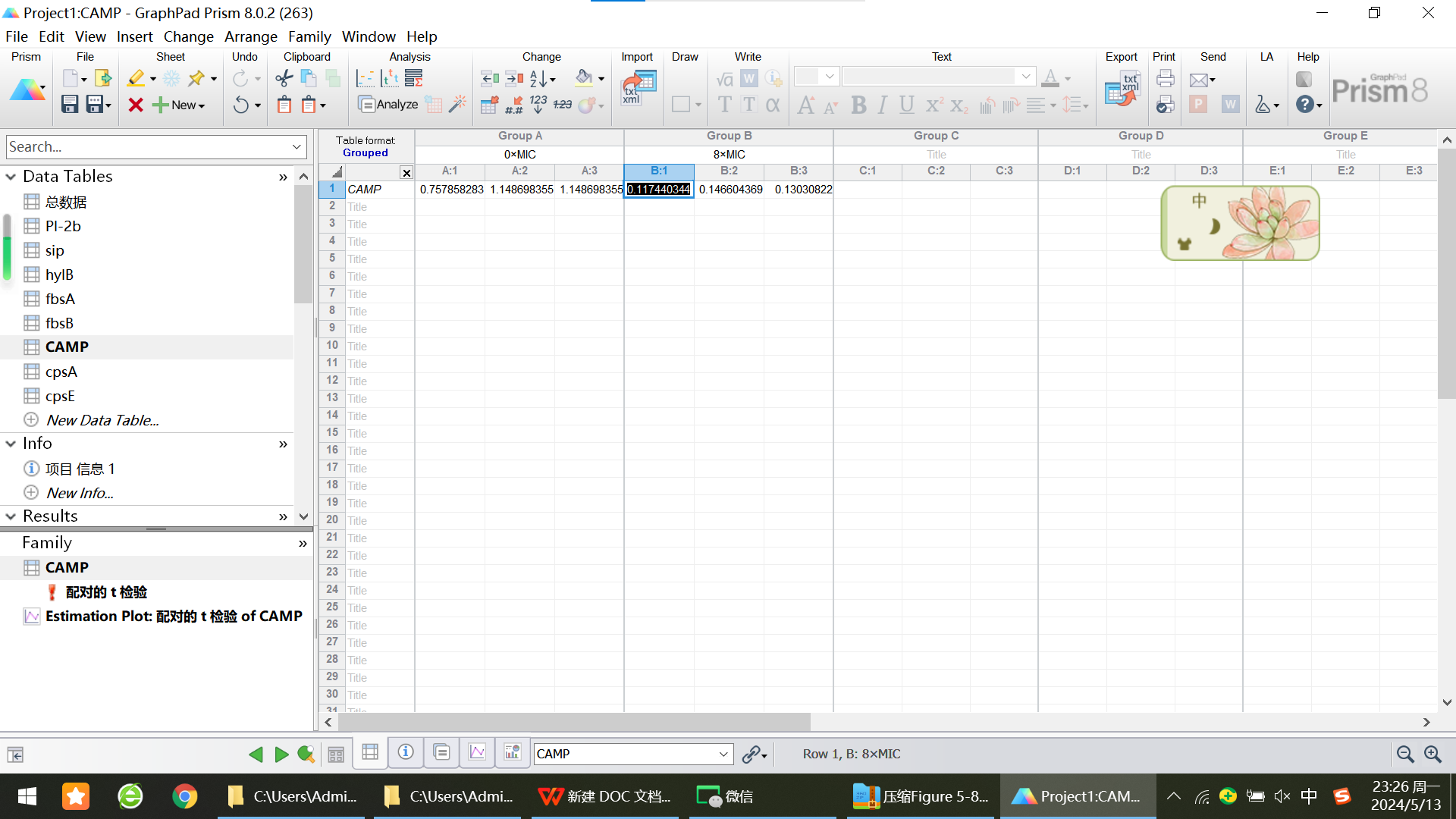

Supplement: Supplementary file 2 [file Data_Sheet_2.ZIP › Figure8-qRT-PCR/screenshots of GraphPad Prism 8.0/qp/CAMP/CAMP.png]
